# CO Adsorbates Induced Framework-Associated Low-Valence Co^δ+^ Sites in Co-ZSM‑5 for Ethane Dehydrogenation

**DOI:** 10.1021/jacs.5c09290

**Published:** 2025-09-05

**Authors:** Shaojia Song, Minjie Zhao, Irene Barba-Nieto, Marcos Fernández-García, Xinyu Chen, Yumeng Fo, Riguang Zhang, Zhen Zhao, Patricia Concepción, Jian Liu, Weiyu Song, Chunming Xu

**Affiliations:** † State Key Laboratory of Heavy Oil Processing at Karamay, 74537 China University of Petroleum (Beijing) at Karamay, Karamay, Xinjiang 834000, China; ‡ State Key Laboratory of Clean and Efficient Coal Utilization, Taiyuan University of Technology, Taiyuan, Shanxi 030024, China; § Instituto de Tecnología Química, 16379 Universitat Politècnica de València-Consejo Superior de Investigaciones Científicas (UPV-CSIC), Avenida de los Naranjos s/n, 46022 Valencia, Spain; ∥ Chemistry Division, 8099 Brookhaven National Laboratory, Upton, New York 11973, United States; ⊥ Instituto de Catálisis y Petroleoquímica, Consejo Superior de Investigaciones Científicas (ICP-CSIC), C/Marie Curie 2, 28049 Madrid, Spain; # State Key Laboratory of Heavy Oil Processing, China University of Petroleum, Chang Ping, Beijing 102249, China

## Abstract

The dynamic structural evolution of heterogeneous catalysts is a ubiquitous phenomenon that has attracted a lot of interest. Catalyst reconstruction can occur after appropriate pretreatment, resulting in more efficient active catalysts, which is an attractive but challenging issue. Here, we reveal a CO activation strategy that controls the microenvironment of the Co sites in the high-silica Co-ZSM-5 catalyst (denoted as 0.50Co-Z5(340)), resulting in three times higher initial conversion and superior regeneration durability in the ethane dehydrogenation reaction compared to the same catalyst without CO pretreatment. *In situ* spectroscopy and metadynamics simulations reveal that the Co^2+^ sites in 0.50Co-Z5(340) dislodge from the framework and move toward the nearby Brønsted acid sites, forming framework-associated low-valence Co^δ+^ species. Mechanistic studies indicate that the Co^δ+^ species catalyze ethane C–H bond cleavage *via* an oxidative addition mechanism, and ethylene is produced simultaneously with H* coupling (direct pathway). The promoted C–H bond activation and facile ethylene desorption explain the superior ethane dehydrogenation performance of the herein CO preactivated 0.50Co-Z5(340) catalyst.

## Graphical abstract

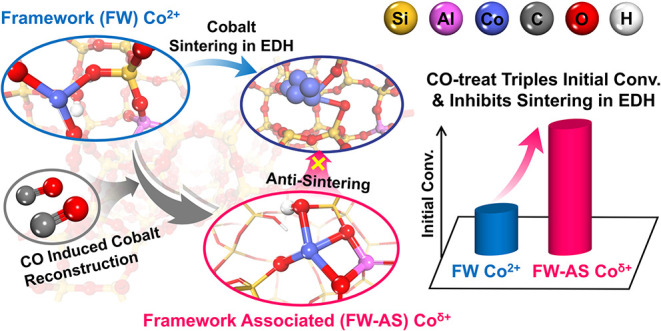

## 1 Introduction

Controlling the structure of active sites at the atomic scale and understanding their nature under reaction conditions are crucial in designing efficient catalysts. Diverse approaches have been developed to construct and stabilize atomically dispersed metal sites in the form of single atoms or subnanometric clusters. A promising strategy involves the use of zeolites, which utilize the confinement effect of micropores and the covalent bonding effect of the zeolite framework.^1^ Despite the protective role of zeolites, dynamic structural evolution is commonly observed for atomically dispersed metal sites during catalysis.^2–6^ This changes the initially designed active sites and complicates the establishment of the structure–performance relationship. Generally speaking, this dynamic evolution can be understood as the adsorbate-induced rearrangement of the active sites.^7^ In this context, the development of appropriate pretreatment or cofeeding strategies can offer new methodologies for tailoring active sites and, consequently, controlling catalytic activity.^8–11^ However, achieving this goal remains challenging and requires a more in-depth understanding of the dynamic response of metal species to changes in the reaction environment, highlighting the necessity of *in situ* spectroscopic, kinetic, and theoretical studies.^12,13^

Alkane dehydrogenation is a promising reaction to produce important light olefins.^14,15^ The ability of atomically dispersed metal site catalysts to facilitate alkane dehydrogenation while preventing side reactions that are favored on larger metal ensembles has attracted widespread attention.^3,16–18^ Moreover, the development of non-Pt alternatives is of both fundamental and industrial importance, with Co-based catalysts emerging as promising candidates.^16,19–28^ In these catalysts, the alkane dehydrogenation performance is influenced by the dynamic evolution of Co sites, which explains the induction periods and pretreatment-dependent activity observed in previous studies,^19–22^ making the identification of functional active sites more complex. According to previous studies, a variety of cobalt species, such as isolated Co sites,^16,20,23–25,28^ small CoO_*x*,_ ^26,27^ and metallic Co clusters,^19^ exhibit varying activities. Especially, the catalytic behavior of isolated Co sites may differ due to distinct electronic properties or coordination environments.^20,23^ This motivates further investigation into modulating isolated Co sites for enhanced activity. In this regard, it has been reported that the framework Al species and Brønsted acid sites (BAS) in aluminosilicate zeolites can modulate or cooperate with metal sites to create new types of active centers with unique reactivity.^29–32^ Inspired by the unique reactivity of framework metal species found in silicate zeolites,^16,23,24^ the simultaneous incorporation of metal and Al centers into the zeolite framework offers a new approach to control the metal structures and the metal–BAS interactions,^33,34^ which may be useful in alkane dehydrogenation but are rarely reported to the best of our knowledge. In this direction, the nature of metal sites, metal–BAS proximity, and their potential dynamic evolution under alkane dehydrogenation conditions remain unanswered questions.

Here we used a high-silica 0.50Co-Z5(340) zeolite, containing framework Co^2+^ with next-nearest-neighbor (NNN) BAS, to characterize the CO-induced dynamic cobalt evolution and its impact on ethane dehydrogenation (EDH). *In situ* spectroscopic characterizations and ab initio molecular dynamics (AIMD) metadynamics (MTD) simulations demonstrate that CO pretreatment creates framework-associated low-valence Co^δ+^ sites by facilitating the migration of framework Co^2+^ species toward NNN-BAS (denoted as (CO)­0.50Co-Z5(340)). The CO-induced Co^δ+^ sites remain stable throughout the EDH reaction and offer superior regeneration durability compared to the same catalyst without CO pretreatment, which produces small Co clusters under working conditions. Moreover, comparative studies on catalytic mechanisms were conducted using a combination of *in situ* spectroscopy, isotopic temperature-programmed surface reaction (TPSR) experiments, and MTD simulations. The results illustrate the superiority of CO-induced Co^δ+^ sites in promoting C–H bond activation while inhibiting overdehydrogenation side reactions, compared to the referenced model catalysts containing framework and exchange Co^2+^ sites.

## 2 Results and Discussion

### 2.1 Synthesis and Characterization of Catalysts

Co-MFI catalysts were synthesized *via* the Co-EDTA-assisted hydrothermal method, using Co-EDTA as the cobalt source to prevent cobalt precipitation in the alkaline reaction media (Figure S1). Catalysts with different cobalt contents and SiO_2_/Al_2_O_3_ ratios were prepared by adjusting the amount of Co and Al sources in the synthesis gel (see details in Section 4). The obtained catalysts are denoted as *x*Co-Z5­(*y*), where *x* and *y* represent the actual cobalt content and SiO_2_/Al_2_O_3_ ratios, respectively, according to the inductively coupled plasma-optical emission spectroscopy (ICP-OES) analysis. The representative 0.50Co-Z5(340), 0.46Co-Z5(110) and Al-free 1.11Co-S1 catalysts all show MFI topology, comparable surface areas, and pore volumes (Figure S2A,B and Table S1). Transmission electron microscopy (TEM) and elemental mapping images suggest the absence of CoO_*x*_ nanoparticles, with Co species homogeneously dispersed within the zeolite crystals (Figure S3). Ultraviolet–visible diffuse reflectance (UV–vis DR) spectra (Figure S2C) indicate that 1.11Co-S1 and 0.50Co-Z5(340) have adsorption bands at 500, 570, and 657 nm, respectively, characteristic of isolated tetrahedral Co^2+^ species within the framework.^24,35^ Raman spectra indicate Si–O–Co bands (1088 and 1224 cm^–1^) in these two samples (Figure S2D).^28^ These characteristic bands were absent or less intense in 0.46Co-Z5(110), suggesting the absence of framework Co^2+^ sites.

In order to investigate the electronic and local structures of the Co sites in the initial catalysts, X-ray adsorption spectroscopy (XAS) experiments were conducted at the Co K-edge. The pre-edge structure, with its centroid located at 7710 eV and attributed to the 1s-3d electronic transition, closely resembled the CoO reference (Figure 1A). Therefore, the shape and intensity are characteristic of a low-spin Co^2+^ oxidation state.^35^ In such an initial state, the higher intensity of the white line (exemplified by the intensity value at 7724.5 eV) with respect to the CoO oxide reference points out the increased degree of the covalent character of the Co­(3d)–O­(2p) interaction in the initial state of the samples with respect to the mentioned reference, which is characteristic of isolated Co^2+^ species.^24^ The EXAFS signals of the three initial, fresh samples were fitted using Co–O and Co–O–Si contributions (Figure S4 and Table S2).^22,24^ Taking 0.50Co-Z5(340) as a representative example, the optimum fitting was obtained using 2.3 (2.8) and O (Si) coordination numbers. There was no need for additional shells to justify the local environment around the Co atoms up to 3.5 Å. The attempts to use Co or Al instead of Si fail to satisfactorily predict the second shell (Figure S5). The XAS results indicate the isolated, coordinatively unsaturated Co^2+^ sites for the three fresh samples, which may be located in the framework or exchange sites of the MFI-type zeolite structures.

**Figure 1:**
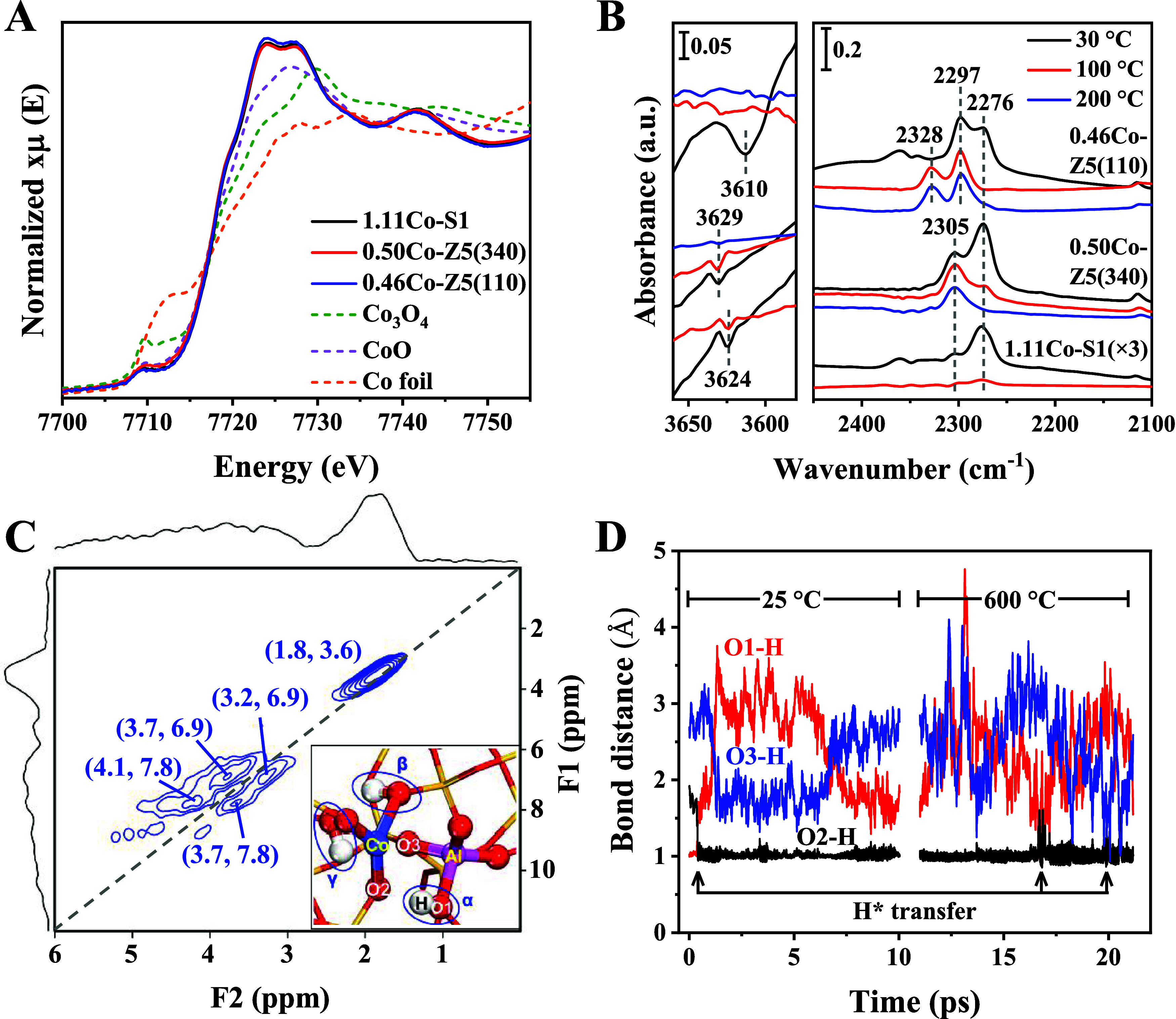
Characterization of as-prepared catalysts. (A) Co K-edge XANES; (B) transmission FTIR spectra with CD_3_CN; (C) ^1^H DQ MAS NMR, inset shows the supposed framework Co^2+^ with the NNN-BAS model for 0.50Co-Z5(340); purple, red, yellow, pink, and white balls represent Co, O, Si, Al, and H atoms, respectively; and (D) AIMD simulations based on the structure in the inset of panel (C) at varying temperatures.

To further examine the nature of the Co sites, CD_3_CN-FTIR experiments were conducted. The characteristic bands at 2276 cm^–1^, appearing for all catalysts, are attributed to CD_3_CN coordinated to silanol (Si–OH) groups.^28^ For 1.11Co-S1 and 0.50Co-Z5(340) catalysts, the characteristic bands at 2305 cm^–1^ are assigned to the chemisorbed CD_3_CN on framework Co^2+^ sites.^20,24,36^ Meanwhile, 1.11Co-S1 and 0.50Co-Z5(340) show negative bands at 3624/3629 cm^–1^ due to CD_3_CN coordinated to Si–OH–Co hydroxyl groups. The 2305 cm^–1^ band is absent in 0.46Co-Z5(110), but it rather exhibits predominant 2328 and 2297 cm^–1^ bands due to CD_3_CN interacting with the exchange Co^2+^ (ref 37) and BAS sites. A similar conclusion is also supported by the pyridine-FTIR results (Figure S6). The spectra reveal characteristic bands at 1610 cm^–1^ for 1.11Co-S1 and 0.50Co-Z5(340), attributed to pyridine interacting with isolated framework Co^2+^ species.^20,24^ The distinct characteristic band at 1624 cm^–1^ in 0.46Co-Z5(110) is assigned to pyridine interacting with the exchange Co^2+^ sites. Therefore, the characterizations suggest that 1.11Co-S1 and 0.50Co-Z5(340) mainly contain framework Co^2+^, while the exchange Co^2+^ sites dominate in the 0.46Co-Z5(110) catalyst. This is because of the low Co/Al ratio (about 0.25) in 0.46Co-Z5(110), as shown in Table S1, which favors the formation of exchange Co^2+^ sites typically stabilized by paired-Al sites.

The *in situ* OH-FTIR spectra in Figure S7 indicate unique temperature-dependent Si–OH–Al bands for 0.50Co-Z5(340). Only Si–OH–Co bands (3621 cm^–1^, ref 36) were observed at 25 °C, while Si–OH–Al bands (3600 cm^–1^) emerged at 600 °C. This suggests a dynamic proton transfer, which we envision is associated with the Co-BAS proximity in 0.50Co-Z5(340). The Co-BAS proximity was directly confirmed by ^1^H DQ MAS NMR experiments (Figure 1C). The analysis of the ^1^H MAS NMR spectra assigned the resonances at around 1.8, 3.7, and 4.1 ppm to the Si–OH, Si–OH–Co, and Si–OH–Al species, respectively (Figure S8). As a result, the autocorrelation peak at (1.8, 3.6) ppm along the diagonal axis (gray dashed) was ascribed to the interaction of Si–OH groups. Notably, the pair of off-diagonal peaks at (3.7, 7.8) and (4.1, 7.8) ppm indicates a spatial proximity between the Si–OH–Co and Si–OH–Al groups (α and β in the inset of Figure 1C), whereas another pair of off-diagonal peaks at (3.7, 6.9) and (3.2, 6.9) ppm reflects the correlations between adjacent Si–OH–Co groups with different chemical environments (β and γ in the inset of Figure 1C). Combined with these results, a configuration, including framework Co^2+^ with NNN-BAS, that is Co^2+^–O–Si–O–Al^3+^ linkage, was inferred for 0.50Co-Z5(340), as illustrated in the inset of Figure 1C. The AIMD simulations based on this model manifest the above-mentioned temperature-dependent Si–OH–Al bands in the OH-FTIR experiments of 0.50Co-Z5(340). Specifically, the bond distance evolution in Figure 1D suggests that the proton of BAS would spontaneously migrate to the O2 site within 0.5 ps at 25 °C. This rationalizes the weak Brønsted acidity of 0.50Co-Z5(340), which shows negligible BAS signals in the OH-FTIR, CD_3_CN-FTIR, and ^1^H NMR spectra under ambient conditions. When the temperature was increased to 600 °C, the protons would be dynamically transferred between the O1, O2, and O3 sites (17 and 20 ps), which produced dynamic BAS species as observed in the OH-FTIR results. For comparison, the models with Co^2+^–(O–Si)_2_–O–Al^3+^ (next–next-nearest-neighbor) or Co^2+^–(O–Si)_3_–O–Al^3+^ (no nearby BAS) linkages are less stable or show no proton transfer (Figures S9 and S10). Therefore, the combined spectroscopic and theoretical results reveal a framework Co^2+^-NNN-BAS structure in the initial 0.50Co-Z5(340) catalyst.

### 2.2 CO Treatment Effects on Ethane Dehydrogenation

Figure 2A shows the EDH performance of the series of Co-MFI samples, and the detailed activity data are provided in Figure 2B and Figure S11. 1.11Co-S1 exhibits an ethane conversion of 7.7% with an ethylene selectivity of 97.6%. For the series of *x*Co-Z5­(*y*) catalysts, the ethane conversion increased upon Al introduction, but this was at the expense of ethylene selectivity. For example, 0.50Co-Z5(340) and 0.46Co-Z5(110) obtained conversions of 14.5 and 20.1% with ethylene selectivities of 92.6 and 74.1%, respectively. The major byproducts of 0.46Co-Z5(110) are benzene and toluene (denoted as BT), and the selectivity reaches 12.7%. The massive aromatic byproducts on 0.46Co-Z5(110) are also confirmed by the larger benzene formation signals in the C_2_H_6_-TPSR experiments (Figure S12). Compared to 0.50Co-Z5(340), which has a similar SiO_2_/Al_2_O_3_ ratio, the 0.38Co-Z5(330) catalyst shows that the increasing Co loading from 0.38 to 0.50 wt % enhances the ethane conversion and ethylene yield. Further increasing the Co loadings to 2 wt % (*i.e.*, the 1.93Co-Z5(410) catalyst) contributes less to the ethylene yield, which was associated with the formation of certain aromatic byproducts. The impregnated Co/Z5-imp, with larger CoO_*x*_ nanoparticles (Figure S13), shows an ethane conversion of 23.5% with an ethylene selectivity of only 53.1%, while the selectivity of aromatics reaches 24.1%.

**Figure 2:**
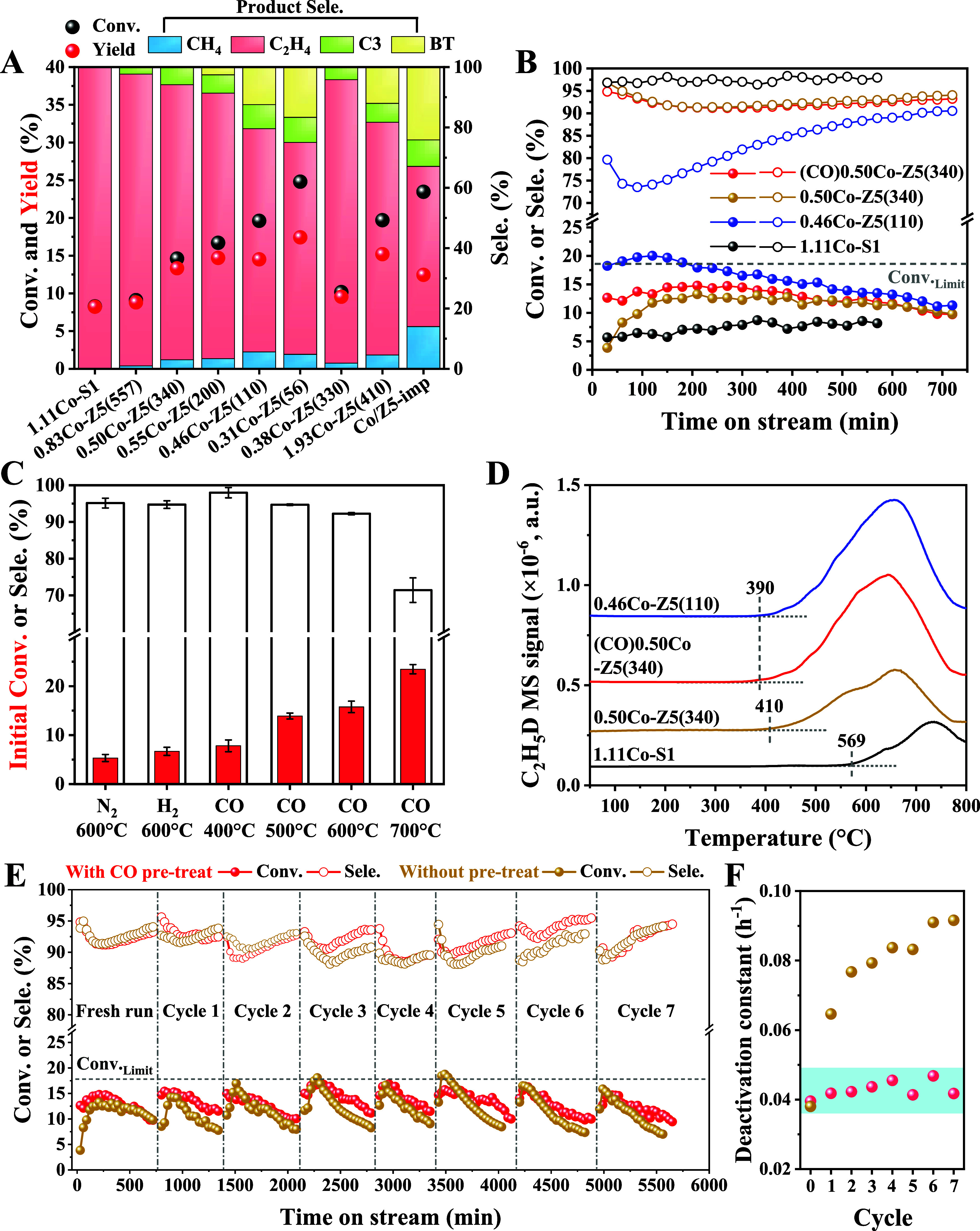
Catalytic performance in the EDH reaction. (A) Ethane conversion and product distribution for various Co-MFI catalysts. Reaction conditions: 0.2 g catalyst, 600 °C, WHSV = 4.73 h^–1^, 80 vol % C_2_H_6_; (B) ethane conversion and ethylene selectivity as a function of time-on-stream; (C) initial ethane conversion and ethylene selectivity of 0.50Co-Z5(340) after different pretreatments; (D) C_2_H_5_D formation signals in D_2_-C_2_H_6_ isotope scrambling TPSR experiments; (E) ethane conversion and ethylene selectivity of 0.50Co-Z5(340) and (CO)­0.50Co-Z5(340) during successive regenerations, and (F) deactivation rate constant in each cycle of panel (E).

It is worth noting that 0.50Co-Z5(340) exhibits an induction period characterized by increasing the ethane conversion from 3.9 to 13.2% during the initial 0–3 h reaction (Figure 2B). We inferred that the activity enhancement was induced by the dynamic Co site evolution driven by Co–C interactions.^38^ Following this conjecture, CO pretreatment (10 vol % CO/N_2_ at 600 °C for 30 min) was explored, successfully eliminating the induction periods without sacrificing ethylene selectivity (Figure 2B,C). Induction periods and the benefit of CO pretreatment are also observed for 0.55Co-Z5(200) and 0.38Co-Z5(330) samples with SiO_2_/Al_2_O_3_ ratio of 200–350. The intrinsic activity of C–H bond activation was evaluated by D_2_-C_2_H_6_ scrambling TPSR experiments (Figure 2D). As an indicator of C–H bond activation capacity,^3,39^ the C_2_H_5_D formation signals follow an order of (CO)­0.50Co-Z5(340) ≈ 0.46Co-Z5(110) > 0.50Co-Z5(340) > 1.11Co-S1. These results suggest that the Co–Al synergy (in all Co-Z5) and CO-induced cobalt evolution (in (CO)­0.50Co-Z5(340)) contribute to C–H bond activation and thereby improve the EDH activity.

0.50Co-Z5­(340) was selected as a representative catalyst to evaluate the stability in successive reaction–regeneration cycles (Figure 2E). This study was conducted on both CO pretreated (CO)­0.50Co-Z5(340) and nonactivated 0.50Co-Z5(340) catalysts. Spent catalysts were regenerated by air calcination (40 mL/min) at 500 °C for 4 h. Similar to the fresh run, regenerated (CO)­0.50Co-Z5(340) underwent CO pretreatment prior to the EDH reaction. As a result, the initial ethane conversion reaches 14.6% after the seventh intermittent regeneration for (CO)­0.50Co-Z5(340), which recovers the fresh run value of 14.8%. The deactivation rate was maintained at about 0.042 h^–1^ for (CO)­0.50Co-Z5(340) during 8 reaction cycles. In contrast, regenerated 0.50Co-Z5(340) (*i.e.*, without CO pretreatment) shows progressively faster deactivation compared to the fresh run, with the deactivation rate increasing to 0.076 h^–1^ for Cycle 2 and 0.092 h^–1^ for Cycle 7 (Figure 2F). This faster deactivation is attributed to more coke deposits according to thermogravimetric analysis, which reveal coke deposits of 2.0% and 7.4% for spent (CO)­0.50Co-Z5(340) and 0.50Co-Z5(340), respectively, after Cycle 7 (Figure S14). In this sense, the results suggest that CO pretreatment not only contributes to the elimination of induction periods in (CO)­0.50Co-Z5(340), but also benefits the regeneration durability. The catalytic activity of (CO)­0.50Co-Z5(340) was further evaluated under varying feed compositions and reaction temperatures. As shown in Figure S15, decreased ethane pressure or increased reaction temperature contribute to higher ethane conversion. Among the explored conditions, the highest ethylene productivity of 2.87 mol_C_2_H_4__·g_metal_^–1^·h^–1^ was obtained using an 80 vol % ethane feed at 600 °C (Table S3). Under comparable reaction conditions (ethane pressure and temperature), (CO)­0.50Co-Z5(340) demonstrates promising performance in terms of ethylene productivity and deactivation rate constant, compared to the reported state-of-the-art Co-based and other non-Pt catalysts.

### 2.3 Study of Cobalt Evolution upon Different Treatments

Collectively, the above results demonstrate the promotive effect of CO pretreatment in eliminating induction periods and improving regeneration durability for (CO)­0.50Co-Z5(340), compared to the nonactivated one. We then combined *in situ* spectroscopic characterizations and MTD simulations to explore CO-induced cobalt evolution and distinguish the working active sites in these two catalysts. The CO-TPR (TPSR), *in situ* Co 2p XPS experiments, TEM techniques, and MTD simulations exclude the reverse water gas shift reaction between the Si–OH–Co species and CO (*e.g.*, Si–OH–Co + CO → Si–Co + CO_2_ + 0.5H_2_), and confirm the retention of the cobalt oxidation state without metallic nanoparticle formation in (CO)­0.50Co-Z5(340) (Figures S16–S18). The OH-FTIR spectra in Figure 3A indicate that the CO treatment weakens the 3620 and 3600 cm^–1^ bands due to Si–OH–Co and Si–OH–Al species in 0.50Co-Z5(340), indicative of framework Co^2+^ evolution associated with the BAS species. In contrast, the OH-FTIR bands remained unchanged for 1.11Co-S1 and 0.46Co-Z5(110) (Figure S19). The transformation of framework Co^2+^ in (CO)­0.50Co-Z5(340) was also corroborated by CD_3_CN and pyridine-FTIR studies, which indicated an attenuation of the characteristic 2305 cm^–1^ and 1610 cm^–1^ bands (due to CD_3_CN and pyridine coordinated to framework Co^2+^)^20,24,36^ compared to 0.50Co-Z5(340) (Figures S6 and S20).

**Figure 3:**
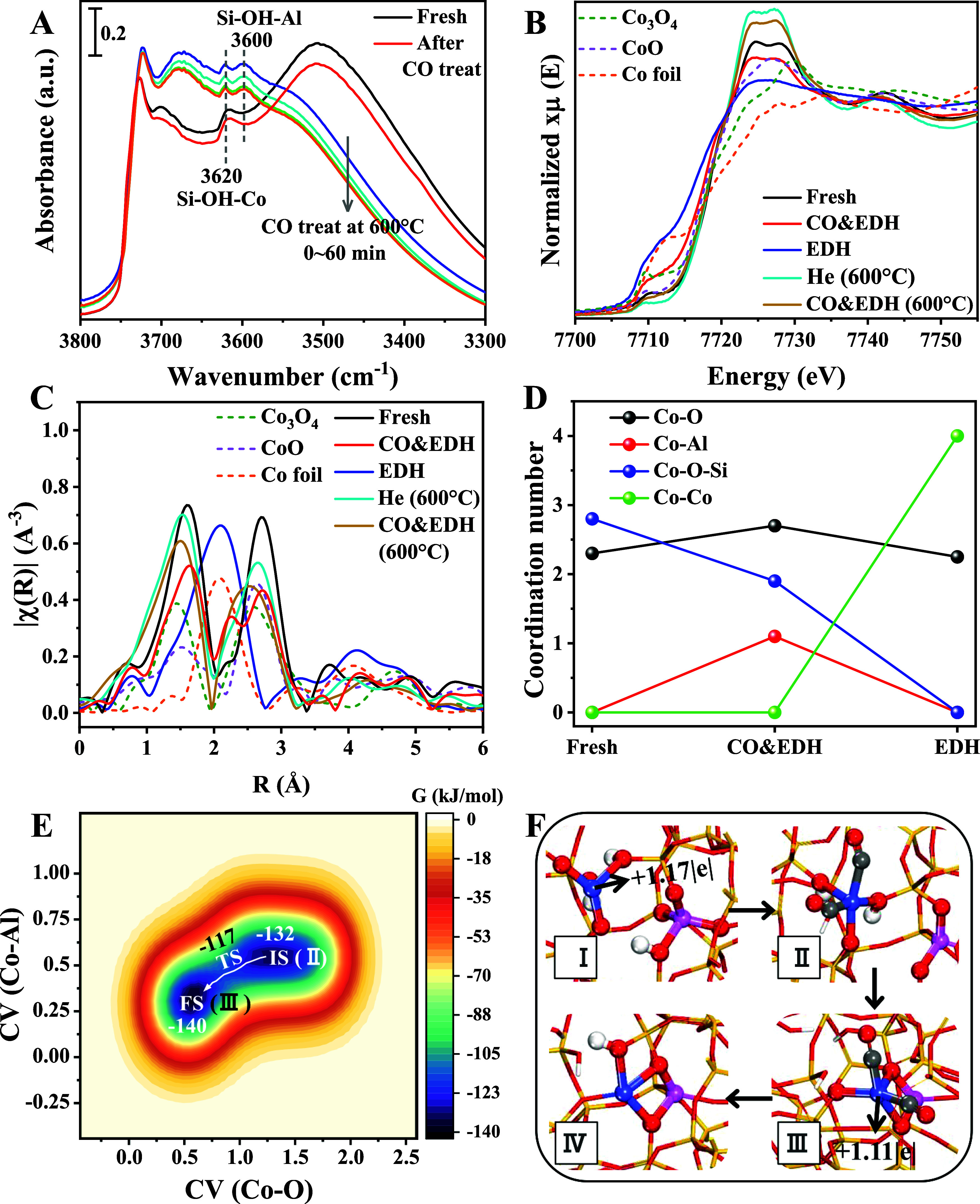
*In situ* measurements for investigating dynamic Co site evolution upon CO pretreatment and the EDH reaction. (A) Transmission FTIR spectra within the hydroxyl region during CO treatment; (B) *in situ* Co K-edge XANES spectra of 0.50Co-Z5(340) during different treatments; (C) *k* ^3^-weigthed FT-EXAFS spectra; (D) coordination number of selected contributions for 0.50Co-Z5(340) after different treatments; (E) free energy surface of MTD simulations of Co site evolution in 0.50Co-Z5(340) with CO adsorption at 600 °C; and (F) schematic diagram of Co site evolution in 0.50Co-Z5(340) upon CO treatment. Purple, red, yellow, pink, and white balls represent Co, Si, Al, and H atoms, respectively.

The local structures of the active sites during the EDH reaction were further determined by *in situ* Co K-edge XAS experiments. Here, CO&EDH, and EDH spectra were collected at 25 °C and represent the catalysts after CO pretreatment followed by EDH, and direct EDH treatment, respectively. The CO&EDH (600 °C) spectrum corresponds to the catalyst in the EDH reaction at 600 °C after CO activation (Figures S21 and S22). The *in situ* XANES spectra in Figure 3B show that the Co absorption edge position of the CO&EDH sample shifts toward lower energy compared to that of the fresh one, which suggests the formation of partially reduced Co sites (Co^δ+^, 0 < δ < 2) in (CO)­0.50Co-Z5(340). The analysis of EXAFS after the CO&EDH treatment requires the presence of an additional shell with respect to the initial sample, with a local environment of Co–O and Co–O–Si. Figure 3C and Table 1 show the presence of a Co–Al shell consisting of a single Co–Al coordination at about 2.73 Å (with phase shift correction), indicating the stabilization of framework-associated Co^δ+^ sites by nearby Al sites (*i.e.*, BAS sites). The presence of an additional Co–Al shell was demonstrated by an F-test,^40^ which showed that the 3-shell fitting improves the variance of the fitting by a probability higher than 99.9%, compared with the two-shell (Co–O and Co–O–Si) model. The Al single neighbor (to Co) was also observed in the EXAFS taken under CO and EDH at 600 °C (Figure S23 and Table 1). Note that the temperature effect on the main structural and electronic features of the local environment can be ruled out considering the similar absorption edge and coordination environment of the He-treated sample at 600 °C compared to the fresh sample (Figure 3B and Table 1). Therefore, the single Al-stabilized Co^δ+^ species are identified as the working active sites in the (CO)­0.50Co-Z5(340) catalyst. In contrast to the CO and EDH samples, metallic Co species are dominant in the direct EDH sample, as revealed by the absorption edge closer to the Co foil in the XANES and the pronounced Co–Co scattering peaks in the EXAFS spectra (Figure 3C and Figure S22). The fitting of the XANES spectrum using the initial sample as the Co^2+^ reference and Co foil as the Co(0) reference suggests that the Co^2+^/Co­(0) species account for 40/60% of the metal content. The EXAFS curve fitting was able to obtain a single Co–O contribution located at 2.01 Å (with phase shift correction), characteristic of the Co^2+^ species, as indicated by the results obtained from the previous cases. The remaining contributions are associated with the metallic Co(0) species. The correction, by the fraction of metallic Co obtained from XANES, renders a first-shell Co–Co coordination number of about 4 (Figure 3D and Table 1), indicating the presence of small (less than 15 atoms) Co clusters.

**Table 1:** EXAFS Fitting Parameters of 0.50Co-Z5(340) under Different Treatments

catalysts	shell	CN^a^	*R*/Å^b^	Δσ^2^/Å^2^ ^c^	*E*_0_/eV^d^	fitting window
fresh	Co–O	2.3	2.07	0.005	2.1	*k*: 2.57–11.42 Å^–1^ *R*: 0.94–3.10 Å
	Co–Si	2.8	3.29	0.0002	–2.8	
He (600 ^o^C)	Co–O	2.3	2.01	0.006	–2.1	*k*: 2.55–9.31 Å^–1^ *R*: 1.09–3.17Å
	Co–Si	2.8	3.26	0.004	–1.6	
CO&EDH	Co–O	1.7	2.05	0.002	4.3	*k*: 2.59–10.30 Å^–1^ *R*: 1.01–3.18 Å
	Co–Si	2.0	3.27	0.0004	0.1	
	Co–Al	1.0	2.73	0.0003	–5.0	
CO&EDH (600°C)	Co–O	2.7	2.01	0.008	0.3	*k*: 2.61–10.33 Å^–1^ *R*: 1.01–3.19 Å
	Co–Si	1.9	3.26	0.0017	–1.3	
	Co–Al	1.1	2.77	0.0014	1.8	
EDH	Co–O	0.9	2.01	0.0025	4.8	*k*: 2.51–10.95 Å^–1^ *R*: 1.14–4.50 Å
	Co–Co	2.4	2.51	0.006	2.6	
	Co–Co	0.8	3.51	0.006	2.6	
	Co–Co	1.8	4.35	0.006	2.6	
	Co–Co	2.3	4.88	0.006	2.6	

a CN, coordination number.

b *R*, bonding distance.

c Δσ^2^, Debye–Waller.

d *E*_0_, inner potential shift; standard error for the above parameters is *R*; 0.01 Å; CN, 9.2%; Δσ^2^, 10.5%; *E*_0_ 0.4 eV; see fitting curves in Figure S23.

The molecular Co site evolution upon CO interactions was further examined by MTD simulations. The framework Co^2+^ with an NNN-BAS structure with two adsorbing CO molecules was used as the initial model (*structures* I and II in Figure 3F) because the Co­(CO)_2_ configuration is energetically more favorable than the Co­(CO) or Co­(CO)_3_ cases (Figure S24). The CO adsorbates weaken the interaction between cobalt and oxygen atoms of the zeolite framework, as revealed by the decreased integral crystal orbital Hamilton population (COHP) values (Figure S25), which contribute to the mobile Co­(CO)_2_ species, as indicated by the trajectories in the MTD simulations (Supporting Movie 1). The collective variables (CVs) in the MTD were set as (1) the coordination number of Co and adjacent O atoms (CV (C–O)) and (2) the distance between Co and Al atoms (CV­(Co–Al)). As a result, the potential energy surface parameterized by the chosen CVs is shown in Figure 3E. The decreased CV­(Co–O) and CV­(Co–Al) suggest the migration of framework Co^2+^ sites toward NNN-BAS upon CO treatment, as illustrated by *structure* III in Figure 3F. This structural evolution can readily occur at 600 °C with a negative Gibbs free energy (Δ*G*) of −8 kJ/mol and a Δ*G* barrier of 15 kJ/mol. The CO desorption energy (Δ*G*) on the newly formed Co^δ+^ sites is only 13.9 kJ/mol, and the CO-TPD temperature is 95 °C (Figures S26 and S27), which rules out the possibility of CO* poisoning. The Bader charge of the Co site in the resultant final *structure* IV is +1.11|e|, which is slightly smaller than that of the initial *structure* I of 1.17 |e|. As *a* result, MTD simulations revealed that the framework Co^2+^ sites would dislodge from the framework toward the NNN-BAS, resulting in low-valence Co^δ+^ sites in (CO)­0.50Co-Z5(340). In contrast, the framework Co^2+^ sites remained stable throughout the MTD simulations in several control models, including the 0.50Co-Z5(340) model without CO* adsorbates, the 1.11Co–S1 model, and the model containing framework Co^2+^ with a next-next-nearest-neighbor BAS and CO* adsorbates (Figure S28). The control simulations illustrate the crucial role of NNN-BAS and CO* adsorbates in producing Co^δ+^ sites in (CO)­0.50Co-Z5(340). Additionally, the CO-induced cobalt dynamic transformation toward Co^δ+^ sites appears position-independent because the formation of Co^δ+^ is also observed on an alternative T12-FW Co^2+^ with the T7-NNN-BAS model (Figure S29). This Co^δ+^ site is metastable in an inert or EDH atmosphere but can revert to the framework site upon O_2_ exposure at 25 °C (Figure S30), which is consistent with the reappearance of induction periods in the activity evaluation for air-exposed (CO)­0.50Co-Z5(340) (Figure S31). As a result, we referred to the *in situ* formed Co^δ+^ species as framework-associated sites hereafter, considering that they were partially dislodged from the framework position and had the ability to reversibly transform to framework Co^2+^ sites.^41,42^ Therefore, the combined XAS and MTD results demonstrate that the NNN-BAS stabilized framework-associated Co^δ+^ sites serve as working sites in the (CO)­0.50Co-Z5(340) catalyzed EDH reaction, while the direct 0.50Co-Z5(340) catalyst evolves small Co metallic clusters in coexistence with residual framework Co^2+^ species (constituting 40% cobalt content). The superiority of the Co^δ+^ sites in terms of their antisintering ability was also supported by UV–vis, Raman spectra, and H_2_-TPR profiles of the regenerated catalysts (Figure S32), which can be rationalized by the stronger interaction between Co and AlO_4_ tetrahedron compared to SiO_4_, as reflected by the COHP analysis (Figure S25). Additionally, the sintering of cobalt species when directly subjecting 0.50Co-Z5(340) to the EDH reaction can be attributed to the C_2_H_4_*-induced migration of the cobalt species, as revealed by MTD simulations (Figure S33).

### 2.4 Mechanism Study

Next, we focused on the catalytic EDH kinetics and mechanisms of the *in situ* formed Co^δ+^ sites. 1.11Co-S1 and 0.46Co-Z5(110), with framework and exchange Co^2+^ sites, were investigated as reference catalysts. Figure S34 shows similar C_2_H_6_-TPD temperatures for the investigated catalysts, suggesting that ethane adsorption was not the major cause of the distinct catalytic activity. Figure S35 shows the H-D kinetic isotope effects (*k*_H_/*k*_D_), measured by replacing C_2_H_6_–H_2_/C_2_H_6_-D_2_ reactants, which are close to 1 for all catalysts, suggesting that the H–H/D dissociation/coupling is not the rate-determining step (RDS).^43^ In this sense, the C–H bond activation should be the RDS, and its mechanism was focused. As illustrated in Figure 4A, alkane C–H bond activation can proceed *via* oxidative addition and σ-bond metathesis mechanisms, which can be distinguished by monitoring the binding sites of detached hydrides from C–H bond cleavage.^44^ Therefore, the characteristic Si–OH–Co bands during the EDH reaction were monitored by FTIR spectroscopy. As a result, 0.46Co-Z5(110) generates increased hydroxyl bands within the 3600–3750 cm^–1^ region with an elongating C_2_H_6_ flow (Figure 4B,C; see full spectra in Figure S36). This suggests the participation of O atoms in abstracting hydrides from ethane C–H bond activation for the 0.46Co-Z5(110) catalyst,^26^ which is indicative of the σ-bond metathesis mechanism. In contrast, the hydroxyl bands are flat for 1.11Co–S1 and (CO)­0.50Co-Z5(340), which is compatible with the oxidative addition mechanism. The mechanisms were further distinguished using deuterium-labeling experiments. First, D_2_ treatment can deuterate Si–OH–Co groups or dissociate on the exchange Co sites to produce Si–OD–Co species based on D_2_-FTIR experiments (Figure S37). Then, D-labeled catalysts were tested in C_2_H_6_-TPSR experiments, as shown in Figure 4D. In particular, the C_2_H_5_D signals emerged earlier than those of H_2_ for 0.46Co-Z5(110). This indicates that the C–H bond activation involves H-D exchange between the OD groups and C_2_H_6_ (ref 24), again revealing a σ-bond metathesis mechanism on 0.46Co-Z5(110). For the other catalysts, the earlier H_2_ formation than C_2_H_5_D/HD suggests that the O/OD species were not involved in C–H bond cleavage, which is consistent with the oxidative addition mechanism. In addition, D-labeled (CO)­0.50Co-Z5(340) shows both C_2_H_5_D and HD formation signals in C_2_H_6_-TPSR experiments, which is different from 1.11Co-S1 and 0.46Co-Z5(110) with predominant HD and C_2_H_5_D signals (Figure S38). Together with the structural characterization, the coexistence of C_2_H_5_D and HD signals is a unique catalytic characteristic of the Co^δ+^ sites in (CO)­0.50Co-Z5(340).

**Figure 4:**
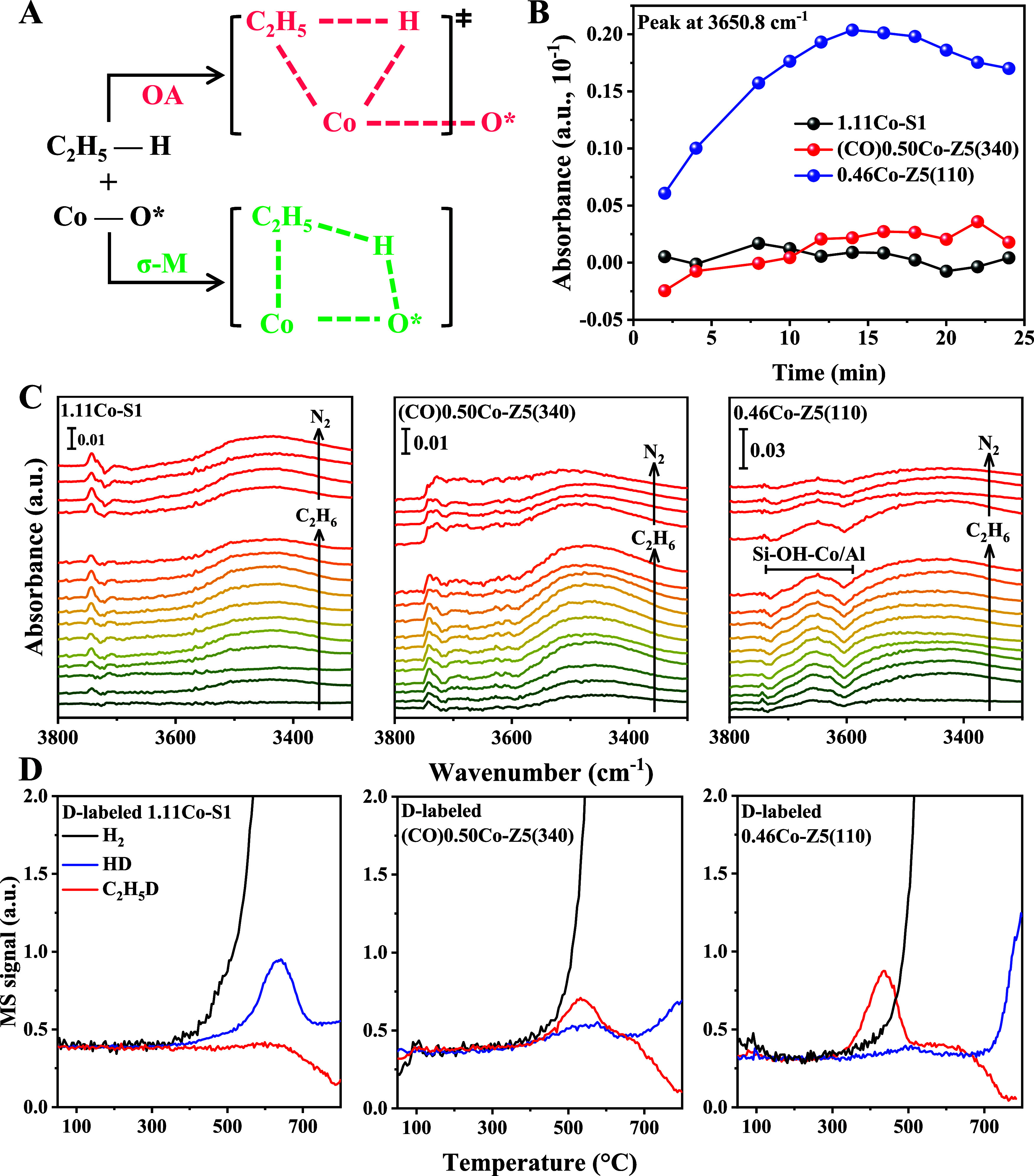
Experimental investigation of the catalytic mechanism. (A) Schematic diagrams of oxidative addition (OA) and σ-bond metathesis (σ-M) mechanisms; (B) evolution of the band intensity at 3651 cm^–1^ *versus* reaction time; (C) time-dependent transmission FTIR spectra with C_2_H_6_ flow at 400 °C; and (D) C_2_H_6_-TPSR profiles of various D-labeled catalysts.

The molecular EDH mechanism was further explored *via* MTD simulations. Based on the structural characterizations, framework-associated Co^δ+^, framework Co^2+^, and exchange Co^2+^ structures were constructed to model the active sites in (CO)­0.50Co-Z5(340), 1.11Co-S1, and 0.46Co-Z5(110) catalysts, respectively. CVs for investigating ethane activation were set as the coordination number of C–H (in adsorbed C_2_H_6_*, CV­(C–H)) and Co–C bonds (Co sites and nearby C atom in adsorbed C_2_H_6_*, CV (C–Co)).^45^ No specific reaction sites (Co site or Co–O pairs, corresponding to oxidative addition or σ-bond metathesis mechanisms) were imposed in this set of CVs. Therefore, MTD simulations should allow the localization of the most favorable mechanisms for C–H bond activation, which avoids the limitations of preset reaction pathways in the static transition state search approach. The free energy surface of representative Co^δ+^ is depicted in Figure 5A, and the key intermediate models are illustrated in Figure 5C. The ethane C–H bond activation on Co^δ+^ sites shows a three-center [C_2_H_5_*–Co–H*] transition state (TS1). Meanwhile, the evolution of the Bader charge on the Co sites [Bader­(Co)] and the integral density of state analysis suggest electron depletion on the Co sites during ethane activation (Figures 5D and Figure S39). Therefore, the results suggest that the Co^δ+^ sites activate the C–H bond *via* an oxidative addition mechanism,^46^ which is compatible with the *in situ* XANES spectra, C_2_H_6_-FTIR, and deuterium-labeling experiments. For comparison, the preset σ-bond metathesis ethane C–H bond cleavage, based on the CI-NEB method, shows decreased Bader­(Co) values (orange symbols in Figure 5D) and the four-center [C_2_H_5_*–Co–O–H*] transition state (TS, Figure S40), which is inconsistent with the above experimental observations. The CVs for monitoring ethylene formation from ethyl intermediates (C_2_H_5_*) were set as the coordination numbers of the terminal C–H (CV (C–H)) and Co–H (in the terminal CH_3_ of C_2_H_5_*, CV (Co–H)). As shown in Figure 5A,C, Co sites can abstract H* from the terminal CH_3_ group in C_2_H_5_* to directly produce ethylene and H_2_ products through a [C_2_H_5_*–Co–H*­(H*)] transition state (TS2). This direct pathway can promote the kinetics of ethylene production and H* removal, enhancing the EDH activity.

**Figure 5:**
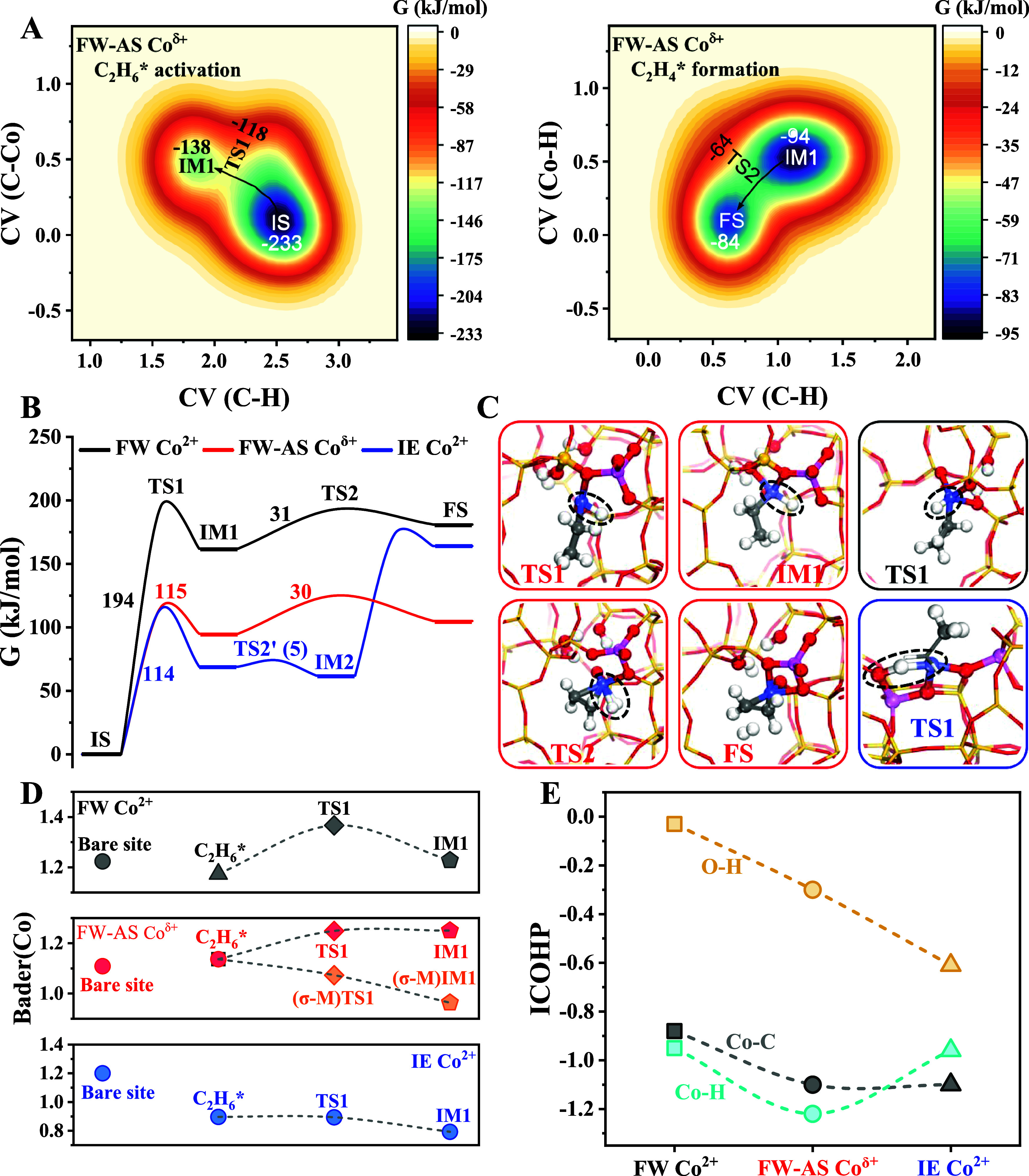
Theoretical catalytic mechanism study. (A) Free energy surface of ethane activation and ethylene formation on framework-associated Co^δ+^; (B) reaction energy profiles on various Co sites; (C) structural models of key intermediates or transition states based on MTD simulations, the red (black/blue) boxes and texts indicate structures on framework-associated Co^δ+^ (framework Co^2+^/exchange Co^2+^) sites, purple, red, yellow, pink, and white balls represent Co, O, Si, Al and H atoms, respectively; (D) evolution of the Bader­(Co) charge along with ethane activation coordinates in MTD simulations, where Bader­(Co) represents the positive charges and is defined as the difference of calculated Bader charge and the valence electrons (9) of cobalt; and (E) ICOHP values of interactions between Co/O atoms and C_2_H_5_*/H* species.

MTD simulations with similar CVs were conducted on the framework and exchange Co^2+^ sites. As a result, the obtained energy profiles and key intermediates are summarized in Figure 5B,C, and the detailed free energy surface and intermediate structures are shown in Figure S41. The reaction mechanism and pathway on framework Co^2+^ sites are similar to those of Co^δ+^, but ethane activation requires a higher barrier, which accounts for the inferior EDH activity of 1.11Co-S1. As for the exchange Co^2+^ sites, ethane C–H bond activation follows a σ-bond metathesis mechanism, as indicated by the four-center [C_2_H_5_*–Co–H*–O] transition state (Figure 5C and Figure S41, S42), as well as the slight decrease in the Bader­(Co) values (Figure 5D). Ethylene formation proceeds *via* an indirect pathway that involves a β-H elimination step for C_2_H_4_* formation (TS2′) and a subsequent H* coupling step with a [C_2_H_5_*–Co–H*···H*–O] transition state (TS3). The H* coupling step on the exchange Co^2+^ sites requires a Δ*G* barrier as high as 111 kJ/mol. Such sluggish H_2_* formation kinetics enhance H* coverage, which is consistent with the markedly increased hydroxyl bands in the above-described C_2_H_6_-FTIR spectra, as well as the more negative hydrogen reaction orders for 0.46Co-Z5(110) (Figure S43).

The reaction energy profiles in Figure 5B indicate that ethane activation is the most energy-demanding step and serves as the RDS. Therefore, COHP analysis was employed to evaluate the interactions between the catalyst and C_2_H_5_*/H* intermediates at the TS1 state (Figure S44). Figure 5E shows the integral COHP results, which quantitatively describe the bond strengths of the Co–C_2_H_5_*, Co–H*, and O–H* interactions. The larger integral COHP values of the Co–C interactions indicate that framework-associated Co^δ+^ and exchange Co^2+^ sites can better stabilize C_2_H_5_* intermediates compared to framework Co^2+^. In addition, COHP analysis also confirmed that the Co^δ+^ sites have the strongest binding strength with the H* species. Therefore, the enhanced Co–C and Co–H interactions help stabilize key TS1 coordination on the Co^δ+^ sites toward a decreased Δ*G* barrier of ethane C–H bond activation as the RDS.

On the other hand, ethylene selectivity was evaluated by comparing the C_2_H_4_* desorption energy (Δ*G*_des_) and overdehydrogenation barrier (Δ*G*_overdehy_). C_2_H_4_* is easy to further dehydrogenate on the exchange Co^2+^ sites with a Δ*G*_overdehy_. of only 17 kJ/mol, while Δ*G*_des_ is 105 kJ/mol, which is much larger than those of the framework-associated Co^δ+^ and framework Co^2+^ sites of 36 and 70 kJ/mol (Figure S45). The results indicate limited ethylene desorption on the exchange Co^2+^ sites, which is consistent with the *in situ* C_2_H_6_-FTIR and C_2_H_4_-TPD experiments (Figures S34B and S36). This rationalizes the inferior ethylene selectivity and considerable aromatic byproducts of 0.46Co-Z5(110). As for framework Co^2+^ and framework-associated Co^δ+^ sites, C_2_H_4_* prefers to desorb (Δ*G*_des_ = 70 and 36 kJ/mol) over further dehydrogenation (Δ*G*_overdehy_ = 112 and 82 kJ/mol), which thereby contributes to the high ethylene selectivity of 1.11Co-S1 and (CO)­0.50Co-Z5(340) catalysts.

Based on the above results, we can conclude that our described CO-induced framework-associated Co^δ+^ sites show superior intrinsic activity compared to framework Co^2+^ sites and comparable intrinsic activity to exchange Co^2+^ sites. Meanwhile, the *in situ* formed Co^δ+^ sites do not require a low SiO_2_/Al_2_O_3_ ratio, and the Co^δ+^ sites enable favorable ethylene desorption instead of overdehydrogenation on exchange Co^2+^ sites, which contributes to ethylene selectivity. In this context, the Co^δ+^ sites in (CO)­0.50Co-Z5(340) are promising for alkane dehydrogenation reactions.

## 3 Conclusions

In summary, this work demonstrates the dynamic evolution of Co sites induced by CO treatment in the high-silica 0.50Co-Z5(340) catalyst, which enhances the EDH activity and regeneration durability. The CO pretreatment induces the migration of Co sites from the framework to the nearby BAS sites, generating unique framework-associated Co^δ+^ sites in the (CO)­0.50Co-Z5(340) catalyst. The Co^δ+^ sites lower the activation barriers for ethane C–H bond cleavage and promote efficient ethylene desorption, preventing overdehydrogenation. This results in a higher ethylene production efficiency compared to catalysts containing framework Co^2+^ sites (1.11Co-S1) and exchange Co^2+^ sites (0.46Co-Z5(110)). Moreover, the Co^δ+^ sites remain stable in the EDH reaction during successive regeneration–reaction cycles, which show superior regeneration durability compared to the counterpart without CO pretreatment. Comparative studies have revealed that Co^δ+^ and framework Co^2+^ sites catalyze ethane C–H bond cleavage *via* an oxidative addition mechanism, and ethylene production follows a direct two-step pathway. In contrast, the exchange Co^2+^ sites follow an indirect three-step pathway involving a σ-bond metathesis mechanism for C–H bond activation. This study provides valuable insights into the nature of the active sites and the catalytic mechanisms underlying the EDH reaction. Additionally, the dynamic Co^δ+^ sites may have potential applications in other Co-catalyzed reactions.

## 4 Experimental Section

### 4.1 Catalyst Synthesis

For the synthesis of 0.50Co-Z5(340) and 0.46Co-Z5(110), 13 g of 25 wt % TPAOH aqueous solution and 0.16 g of ethylenediaminetetraacetic acid disodium cobalt salt hydrate (Co-EDTA-2Na) were added to 15 g of deionized water. After the mixture was stirred for 1 h, 0.03 or 0.08 g of aluminum isopropoxide was added to the above mixture, which corresponded to 0.50Co-Z5(340) and 0.46Co-Z5(110). After the mixture was stirred for 1 h, 8.32 g of TEOS was added dropwise to the solution and stirred for 4 h. The obtained mixture was transferred into a Teflon-lined stainless-steel autoclave and heated to 170 °C for 3 days. The resulting sample was collected and washed with deionized water and ethanol 3 times, dried at 80 °C for 12 h, and subsequently calcined in a muffle furnace at 550 °C for 6 h at a heating rate of 2 °C·min^–1^. The obtained Na-form samples were transformed to H-form *via* ion-exchange in 0.5 M NH_4_Cl aqueous solution (1g catalyst per 20 mL) for 12 h at 30 °C. The actual Co/Al molar ratios were determined to be 0.86 and 0.25 for the 0.50Co-Z5(340) and 0.46Co-Z5(110) samples, respectively, according to ICP-OES analysis. A series of *x*Co-Z5­(*y*) catalysts was also synthesized by regulating the addition of Co­(NO_3_)_2_·6H_2_O, EDTA-2Na, and aluminum isopropoxide in the synthesis gel, where *x* and *y* denote the actual Co loading (*x* wt %) and SiO_2_/Al_2_O_3_ ratio, respectively. Al-free 1.11Co-S1 or parent ZSM-5 (Z5) support was synthesized using the same procedure as Co-Z5, except that aluminum isopropoxide or Co-EDTA-2Na were not added in synthesis gel. Co/Z5-imp was synthesized *via* the impregnation method, and the nominal Co loading was 1.0 wt %.

### 4.2 Catalyst Characterization

Catalyst crystallinity was examined by powder X-ray diffraction analysis (Bruker D8 ADVANCE) with Cu Kα radiation (λ = 1.56 Å). UV–vis DR spectra were recorded on a SHIMADZU UV-3600 spectrophotometer. Raman spectra were collected using a Renishaw inVia instrument with an excitation laser of 325 nm. The N_2_ physisorption isotherms of the catalysts (degassed in vacuum at 120 °C for 2 h) were measured at −196 °C using an Anton Paar Autosorb iQ instrument. XPS spectra were recorded on a Thermo ESCALAB 250Xi instrument with monochromatic Al Kα radiation. TEM and elemental mapping images were obtained on an FEI Tecnai G2 F20 microscope. ^1^H magic-angle spinning NMR spectra of the dehydrated catalysts (vacuum, 350 °C for 12 h) were recorded on a Bruker Avance III 800 spectrometer. All *in situ* FTIR experiments were conducted on a Nicolet iS50 spectrometer equipped with an MCT detector and transmission cell. All of the spectra were recorded at 32 scans with a resolution of 4 cm^–1^. Temperature-programmed surface reaction (TPSR) experiments, including D_2_-C_2_H_6_ isotope scrambling TPSR and C_2_H_6_-TPSR over deuterium-labeled catalysts, were performed in a fixed-bed reactor equipped with a Hiden ExQ mass spectrometry (MS) detector. CO, CO_2_, C_2_H_4_, C_2_H_5_D, H_2_, D_2_, HD, benzene, and toluene were detected by MS signals with *m*/*z* = 28, 44, 28, 31, 2, 4, 3, 78, and 91, respectively. More details about the FTIR and TPSR experiments can be found in Supporting Information. *In situ* XAS spectroscopy was performed in the fluorescence mode at the CLÆSS 22 beamline of the ALBA synchrotron. The XAS data were obtained by merging and calibrating multiple scans: five scans for each of the three initial fresh samples, four scans for 0.50Co-Z5(340) during He treatment at 600 °C, ten scans for 0.50Co-Z5(340) during CO&EDH treatment at 600 °C, and five scans for 0.50Co-Z5(340) after CO&EDH or direct EDH treatment followed by cooling to 25 °C. Details regarding the XAS spectra collection and EXAFS fitting are described in Supporting Information.

### 4.3 Catalyst Testing

The catalytic performance was measured at 600 °C in a fixed-bed reactor (6 mm inner diameter, 400 mm length). 0.2 g of the catalyst (40–60 mesh) was loaded into the reactor. The catalysts were heated to 600 °C at a ramp rate of 10 °C/min in N_2_. The reaction was started by switching N_2_ to the reaction mixture of (C_2_H_6_/N_2_ = 4:6 mL/min). The weight hourly space velocity (WHSV) of ethane for the activity evaluation was 2.4 h^–1^. The products were analyzed by an online gas chromatograph (GC-9890 B) equipped with an FID detector (HP-PLOT Q column) for analyzing CH_4_, C_2_H_4_, C_3_H_6_, C_3_H_8_, and aromatics (benzene, toluene) and a TCD detector for analyzing CO and CO_2_.

Ethane conversion and product selectivity were calculated as follows

$$\mathrm{Conv}.=\frac{\sum n_{x}C_{x}-2\times C_{C_{2}H_{4}}}{\sum n_{x}C_{x}}$$

$$\mathrm{Sele}.=\frac{n_{x}\times C_{x}}{\sum n_{x}C_{x}-2\times C_{C_{2}H_{4}}}$$

where *x* is the product in the outlet gas flow (CO, CO_2_, CH_4_, C_2_H_4_, C_2_H_6_, C_3_H_6_, C_3_H_8_, C_4_H_10_, C_6_H_6_, and C_7_H_8_), *n*_*x*_ is the number of carbon atoms in product *x*, and *C*_*x*_ is the relative amount of product *x* determined by the corresponding calibration factors.

The carbon balances were calculated using the following equation

$$carbon\;balances=\frac{\sum n_{x}C_{x}}{2\times \left({{[C}_{C_{2}H_{6}}]}_{\mathrm{in}}-{{[C}_{C_{2}H_{6}}]}_{\mathrm{out}}\right)}\times 100\%$$

where [*C*_C_2_H_6__]_in_ and [*C*_C_2_H_6__]_out_ represent the relative amounts of ethane in the inlet and outlet, respectively. As a result, the carbon balances in the experiments were found to be better than 95%.

The deactivation rate constant (*K*_d_, h^–1^) was calculated using the following equation^14^

$$K_{d}=\left(\ln\left[\frac{\left(1-{\mathrm{Conv}.}_{\mathrm{final}}\right)}{{\mathrm{Conv}.}_{\mathrm{final}}}\right]-\ln\left[\frac{\left(1-{\mathrm{Conv}.}_{\mathrm{initial}}\right)}{{\mathrm{Conv}.}_{\mathrm{initial}}}\right]\right)/t$$

where Conv._final_ and Conv._initial_ represent the ethane conversions at the final and initial reactions, respectively, and *t* is the reaction time.

### 4.4 Theoretical Calculation Method

All AIMD simulations were conducted within the CP2K software package (CP2K 2023.2),^47^ while the AIMD trajectories were visualized using VMD 1.9.4.^48^ The PBE exchange-correlation functional, DZVP basis set, and GTH pseudopotentials were used in the DFT calculations, and Grimme’s D3 method was considered for dispersion forces. A kinetic cutoff energy of 400 Ry and SCF convergence criteria of 10^–5^ au were used for all calculations. The calculations were performed under the NVT ensemble, and a temperature of 600 °C was maintained using Nose–Hoover thermostats. The time step of the AIMD in the equations of motion was set at 0.5 fs. The established models were pre-equilibrated until the energy and temperature remained basically unchanged to generate the initial configurations. Then, MTD simulations were employed to map the free energy surface that relied on historical events along specific CVs, which helped gain insights into the active site structures and catalytic mechanisms. All MTD simulations were implemented using the PLUMED program, and the Gaussian potential hills were applied to the system along selected CVs, which accelerated sampling along the reaction coordinate and helped to landscape the reaction mechanism. The hills take the following form

$$V\left(t,s\left(t\right)\right)=w\sum_{n}e^{-\left(1/2\right)(s\left(t\right)-s{\left(n\cdot t_{G}/\delta_{G}\right)}^{2}}$$

Here, *w* and δ_G_ represent the height and width of the applied Gaussian hills, which were set as 2 kJ/mol and 0.2 au in the MTD simulations, respectively, and *t*_G_ = 25 fs as the time interval between two consecutive Gaussian hills. In addition, quadratic walls were used to restrict the CVs to the regions of the free energy surface of interest. The coordination numbers or distances between the desired atoms were set as CVs for the MTD simulations, and the details are described in Supporting Information. In addition, to ensure the accuracy of the reaction pathways explored using MTD, we employed static transition state searches to define and compare multiple distinct paths for ethane activation. The detailed methods for static transition state searches and the electronic analysis of representative MTD trajectories are also described in Supporting Information.

A Si_96_O_192_ moiety was used to model the MFI crystal. The 1.11Co-S1 structure was constructed by replacing one T1-Si atom with a Co atom. Two protons were introduced to maintain the skeleton electron neutrality after Co^2+^ substitution. 0.50Co-Z5(340) was modeled by introducing a NNN-BAS at the T4 site considering three aspects: (1) the framework Co^2+^ with the nearby BAS sites was identified by experimental characterization; (2) Lowenstein’s rule restricts the M–O–Al configuration, but the NNN configuration with M–O–Si–O–Al linkage is allowed; and (3) the T4 site is the most energy favorable for the BAS moiety among the three NNN candidacies (T3, T4, T6) for the framework Co^2+^ at the T1 site. Additionally, the 0.46Co-Z5(110) structure was constructed by placing one Co atom in a sinusoidal channel at the exchange position, which was stabilized by T_1_–T_10_ Al pairs.

## Supplementary Material





## Notes

Detailed characterization and experimental data (PDF)
Dynamic trajectories of CO-induced cobalt evolution based on metadynamics simulations (MP4)

## References

[1] Liu L., Corma A. (2021). Confining isolated atoms and clusters in crystalline porous materials
for catalysis. Nat. Rev. Mater..

[2] Hong H., Xu Z., Mei B., Hu W., Fornasiero P., Wang C., Wang T., Yue Y., Li T., Yang C., Cui Q., Zhu H., Bao X. (2025). A self-regenerating
Pt/Ge-MFI zeolite for propane dehydrogenation with high endurance. Science.

[3] Zeng L., Cheng K., Sun F., Fan Q., Li L., Zhang Q., Wei Y., Zhou W., Kang J., Zhang Q., Chen M., Liu Q., Zhang L., Huang J., Cheng J., Jiang Z., Fu G., Wang Y. (2024). Stable anchoring of single rhodium atoms by indium
in zeolite alkane
dehydrogenation catalysts. Science.

[4] Moliner M., Gabay J., Kliewer C., Carr R., Guzman J., Casty G., Serna P., Corma A. (2016). Reversible transformation
of Pt nanoparticles into single atoms inside high-silica chabazite
zeolite. J. Am. Chem. Soc..

[5] Liu L., Zakharov D., Arenal R., Concepción P., Stach E., Corma A. (2018). Evolution and stabilization
of subnanometric
metal species in confined space by *in situ* TEM. Nat. Commun..

[6] Zhao M., Li C., Gómez D., Gonell F., Diaconescu V., Simonelli L., Haro M., Calvino J., Meira D., Concepción P., Corma A. (2023). Low-temperature hydroformylation
of ethylene by phosphorous stabilized Rh sites in a one-pot synthesized
Rh-(O)-P-MFI zeolite. Nat. Commun..

[7] Newton M. A. (2008). Dynamic
adsorbate/reaction induced structural change of supported metal nanoparticles:
Heterogeneous catalysis and beyond. Chem. Soc.
Rev..

[8] Paolucci C., Khurana I., Parekh A., Li S., Shih A., Li H., Iorio J., Albrarracin-Caballero J., Yezerets A., Miller J., Delgass W., Ribeiro F., Schneider W., Gounder R. (2017). Dynamic multinuclear sites formed
by mobilized copper
ions in NO_
*x*
_ selective catalytic reduction. Science.

[9] Serna P., Gates B. (2011). Zeolite-supported rhodium
complexes and clusters: Switching catalytic
selectivity by controlling structures of essentially molecular species. J. Am. Chem. Soc..

[10] Liu L., Lopez-Haro M., Lopes C., Rojas-Buzo S., Concepcion P., Manzorro R., Simonelli L., Sattler A., Serna P., Calvino J., Corma A. (2020). Structural
modulation and direct measurement of subnanometric bimetallic PtSn
clusters confined in zeolites. Nat. Catal..

[11] Sun G., Zhao Z., Li L., Pei C., Chang X., Chen S., Zhang T., Tian K., Sun S., Zheng L., Gong J. (2024). Metastable gallium hydride mediates
propane dehydrogenation on H_2_ co-feeding. Nat. Chem..

[12] Shamzhy M., Opanasenko M., Concepción P., Martínez A. (2019). New trends
in tailoring active sites in zeolite-based catalysts. Chem. Soc. Rev..

[13] Meitzner G., Iglesia E., Baumgartner J., Huang E. (1993). The chemical state
of gallium in working alkane dehydrocyclodimerization catalysts. *In situ* gallium K-edge X-Ray absorption spectroscopy. J. Catal..

[14] Chen S., Chang X., Sun G., Zhang T., Xu Y., Wang Y., Pei C., Gong J. (2021). Propane dehydrogenation:
Catalyst development, new Chemistry, and emerging technologies. Chem. Soc. Rev..

[15] Motagamwala A. H., Almallahi R., Wortman J., Igenegbai V., Linic S. (2021). Stable and selective catalysts for propane dehydrogenation operating
at thermodynamic limit. Science.

[16] Zhou H., Li H., Wang L., Chu S., Liu L., Liu L., Qi J., Ren Z., Cai A., Hui Y., Qin Y., Song L., Qin X., Shi J., Hou J., Ding Y., Ma J., Xu S., Tao X., Li L., Yang Q., Hu B., Liu X., Chen L., Xiao J., Xiao F. (2025). Cobaltosilicate zeolite
beyond platinum
catalysts for propane dehydrogenation. Nat.
Catal..

[17] Chen S., Pei C., Sun G., Zhao Z., Gong J. (2020). Nanostructured catalysts
toward efficient propane dehydrogenation. Acc.
Mater. Res..

[18] Kirlin P. S., Gates B. (1987). Activation of the C–C bond provides a molecular basis for
structure sensitivity in metal catalysis. Nature.

[19] Chen C., Zhang S., Wang Z., Yuan Z. (2020). Ultrasmall Co confined
in the silanols of dealuminated beta zeolite: A highly active and
selective catalyst for direct dehydrogenation of propane to propylene. J. Catal..

[20] Yu K., Srinivas S., Wang C., Chen W., Ma L., Ehrlich S., Marinkovic N., Kumar P., Stach E., Caratzoulas S. (2022). High-temperature pretreatment effect on Co/SiO_2_ active sites and ethane dehydrogenation. ACS Catal..

[21] Hu B., Getsoian A., Schweitzer N., Das U., Kim H., Niklas J., Poluektov O., Curtiss L., Stair P., Miller J., Hock A. (2015). Selective
propane dehydrogenation
with single-site Co­(II) on SiO_2_ by a non-redox mechanism. J. Catal..

[22] Xu Y., Hu W., Li Y., Su H., Liang W., Liu B., Gong J., Liu Z., Liu X. (2023). Manipulating the cobalt
species states to break the conversion-selectivity trade-off relationship
for stable ethane dehydrogenation over ligand-free-synthesized Co@MFI
catalysts. ACS Catal..

[23] Qu Z., He G., Zhang T., Fan Y., Guo Y., Hu M., Xu J., Ma Y., Zhang J., Fan W., Sun Q., Mei D., Yu J. (2024). Tricoordinated single-atom cobalt in zeolite boosting
propane dehydrogenation. J. Am. Chem. Soc..

[24] Hu Z.-P., Qin G., Han J., Zhang W., Wang N., Zheng Y., Jiang Q., Ji T., Yuan Z., Xiao J., Wei Y., Liu Z. (2022). Atomic insight
into the local structure and microenvironment
of isolated Co-motifs in MFI zeolite frameworks for propane dehydrogenation. J. Am. Chem. Soc..

[25] Huang Z., He D., Deng W., Jin G., Li K., Luo Y. (2023). Illustrating
new understanding of adsorbed water on silica for inducing tetrahedral
Cobalt­(II) for propane dehydrogenation. Nat.
Commun..

[26] Liu L., Li H., Zhou H., Chu S., Liu L., Feng Z., Qin X., Qi J., Hou J., Wu Q., Li H., Liu X., Chen L., Xiao J., Wang L., Xiao F. (2023). Rivet of cobalt
in siliceous zeolite for catalytic ethane dehydrogenation. Chem.

[27] Lee S., Halder A., Ferguson G., Seifert S., Winans R., Teschner D., Schlögl R., Papaefthimiou V., Greeley J., Curtiss L., Vajda S. (2019). Subnanometer cobalt
oxide clusters as selective low temperature oxidative dehydrogenation
catalysts. Nat. Commun..

[28] Liu Q., Yao Y., Li J., Wang J., Chen L., Li W., Guo Y., Yao S., Yang Y., Wang X. (2025). Stable cobalt-zeolite
propane-dehydrogenation catalysts enabled by reaction-driven reconstruction. Angew. Chem., Int. Ed..

[29] Schreiber M. W., Plaisance C., Baumgärtl M., Reuter K., Jentys A., Bermejo-Deval R., Lercher J. (2018). Lewis-Brønsted acid pairs in
Ga/H-ZSM-5 to catalyze dehydrogenation of light alkanes. J. Am. Chem. Soc..

[30] Yuan Y., Huang E., Hwang S., Liu P., Chen J. (2024). Confining
platinum clusters in indium-modified ZSM-5 zeolite to promote propane
dehydrogenation. Nat. Commun..

[31] Maeno Z., Yasumura S., Wu X., Huang M., Liu C., Toyao T., Shimizu K. (2020). Isolated indium hydrides in CHA zeolites:
Speciation and catalysis for nonoxidative dehydrogenation of ethane. J. Am. Chem. Soc..

[32] Phadke N. M., Mansoor E., Bondil M., Head-Gordon M., Bell A. (2019). Mechanism and kinetics of propane dehydrogenation and cracking over
Ga/H-MFI prepared via vapor-phase exchange of H-MFI with GaCl_3_. J. Am. Chem. Soc..

[33] Lin L., Sheveleva A., Silva I., Parlett C., Tang Z., Liu Y., Fan M., Han X., Carter J., Tuna F., McInnes E., Cheng Y., Daemen L., Rudić S., Ramirez-Cuesta A., Tang C., Yang S. (2020). Quantitative production
of butenes from biomass-derived γ-valerolactone catalysed by
hetero-atomic MFI zeolite. Nat. Mater..

[34] Jiao F., Bai B., Li G., Pan X., Ye Y., Qu S., Xu C., Xiao J., Jia Z., Liu W., Peng T., Ding Y., Liu C., Li J., Bao X. (2023). Disentangling
the activity-selectivity trade-off in catalytic conversion of syngas
to light olefins. Science.

[35] Estes D. P., Siddiqi G., Allouche F., Kovtunov K., Safonova O., Trigub A., Koptyug I., Copéret C. (2016). C–H
activation on Co, O sites: Isolated surface sites versus molecular
analogs. J. Am. Chem. Soc..

[36] Han D., Liu M., Huang C., Sun X., Guan L., He B., Mei Y., Zu Y. (2023). Uniformly
stable hydroxylated cobalt­(II) silicate species
embedded within silicalite-1 zeolite for boosting propane dehydrogenation. Microporous Mesoporous Mater..

[37] Essid S., Ayari F., Bulánek R., Vaculík J., Mhamdi M., Delahay G., Ghorbel A. (2018). Over-and low-exchanged
Co/BEA catalysts: General characterization and catalytic behaviour
in ethane ammoxidation. Catal. Today.

[38] Rahmati M., Safdari M., Fletcher T., Argyle M., Bartholomew C. (2020). Chemical and
thermal sintering of supported metals with emphasis on cobalt catalysts
during Fischer–Tropsch synthesis. Chem.
Rev..

[39] Sun G., Zhao Z., Mu R., Zha S., Li L., Chen S., Zang K., Luo J., Li Z., Purdy S., Kropf A., Miller J., Zeng L., Gong J. (2018). Breaking the scaling relationship
via thermally stable Pt/Cu single
atom alloys for catalytic dehydrogenation. Nat.
Commun..

[40] Chantler, C. ; Boscherini, F. ; Bunker, B. International Tables for Crystallography, Vol. I: X-ray Absorption Spectroscopy and Related Techniques.; John Wiley & Sons, 2024.

[41] Ravi M., Sushkevich V., Bokhoven J. (2021). On the location of Lewis acidic aluminum
in zeolite mordenite and the role of framework-associated aluminum
in mediating the switch between Brønsted and Lewis acidity. Chem. Sci..

[42] Ravi M., Sushkevich V., Bokhoven J. (2020). Towards a better understanding of
Lewis acidic aluminium in zeolites. Nat. Mater..

[43] Gómez-Gallego M., Sierra M. (2011). Kinetic isotope
effects in the study of organometallic
reaction mechanisms. Chem. Rev..

[44] Xie Z., Chen J. (2023). Bimetallic-derived
catalytic structures for CO_2_-assisted
ethane activation. Acc. Chem. Res..

[45] Fo Y., Song S., Yang K., Ji X., Yang L., Huang L., Chen X., Wu X., Liu J., Zhao Z., Song W. (2024). Ab initio molecular dynamics simulation
reveals the influence of entropy effect on Co@BEA zeolite-catalyzed
dehydrogenation of ethane. Chin. J. Catal..

[46] van
Santen R. A., Neurock M., Shetty S. (2010). Reactivity theory of
transition-metal surfaces: A Brønsted-Evans-Polanyi linear activation
energy-free-energy analysis. Chem. Rev..

[47] Kühne T. D., Iannuzzi M., Del Ben M., Rybkin V., Seewald P., Stein F., Laino T., Khaliullin R., Schütt O., Schiffmann F., Golze D., Wilhelm J., Chulkov S., Bani-Hashemian M., Weber V., Borštnik U., Taillefumier M., Jakobovits A., Lazzaro A., Pabst H., Müller T., Schade R., Guidon M., Andermatt S., Holmberg N., Schenter G., Hehn A., Bussy A., Belleflamme F., Tabacchi G., Glöß A., Lass M., Bethune I., Mundy C., Plessl C., Watkins M., VandeVondele J., Krack M., Hutter J. (2020). CP2K: An electronic
structure and molecular dynamics software package - quickstep: efficient
and accurate electronic structure calculations. J. Chem. Phys..

[48] Humphrey W., Dalke A., Schulten K. (1996). VMD: Visual
molecular dynamics. J. Mol. Graphics.

