# Supplementary material for: CO Adsorbates Induced Framework-Associated Low-Valence Coδ+ Sites in Co-ZSM‑5 for Ethane Dehydrogenation
Source: J Am Chem Soc. 2025 Sep 5;147(37):33666–78. doi: 10.1021/jacs.5c09290 (PMC12447503; doi:10.1021/jacs.5c09290)
Supplement: Supplementary file 1 [file ja5c09290_si_001.pdf]

Supporting Information

CO Adsorbates Induced Framework Associated Low-Valence $Co^{\delta+}$ Sites in Co-ZSM-5 for Ethane Dehydrogenation

Shaojia Song[1,2,‡], *Minjie Zhao*[3,‡], *Irene Barba-Nieto*[4], *Marcos Fernández-García*[5], *Xinyu Chen*[6], *Yumeng Fo*[6], *Riguang Zhang*[2], *Zhen Zhao*[6], *Patricia Concepción*[3,*], *Jian Liu*[1,6,*], *Weiyu Song*[1,6,*], *Chunming Xu*[6].

[1] State Key Laboratory of Heavy Oil Processing at Karamay, China University of Petroleum (Beijing) at Karamay, Karamay, Xinjiang 834000, China.

[2] State Key Laboratory of Clean and Efficient Coal Utilization, Taiyuan University of Technology, Taiyuan, Shanxi 030024, China.

[3] Instituto de Tecnología Química, Universitat Politècnica de València-Consejo Superior de Investigaciones Científicas (UPV-CSIC), Avenida de los Naranjos s/n, 46022 Valencia, Spain.

[4] Chemistry Division, Brookhaven National Laboratory, Upton, New York 11973, United States.

[5] Instituto de Catálisis y Petroleoquímica, Consejo Superior de Investigaciones Científicas (ICP-CSIC), C/Marie Curie 2, 28049-Madrid, Spain.

[6] State Key Laboratory of Heavy Oil Processing, China University of Petroleum, Chang Ping, Beijing 102249, China.

‡ These authors contributed equally to this work.

* Correspondence: pconcepc@itq.upv.es (Patricia Concepción), liujian@cup.edu.cn (Jian Liu), songwy@cup.edu.cn (Weiyu Song).

Supporting catalyst characterization method

H_2-TPR and CO-TPR: H_2-TPR and CO-TPR experiments were performed on the HUASI DAS-7200 instrument. The catalysts were pre-treated in N_2 at 600°C for 1 h. The NH_3 pre-adsorption was performed at 50°C for 30 min. Afterwards, the temperature programmed profiles were recorded from 50 to 850°C with a heating rate of 10°C/min.

D_2-C_2H_6 isotope scrambling TPSR: The catalysts were pre-treated in N_2 at 600°C for 1 h. Then, the reactor was cooled down to 50°C, the flow was switched to a 30 mL/min mixture containing 25 vol% C_2H_6 and 5 vol% D_2 (balanced with N_2). After the selected MS signals including CO, CO_2, C_2H_4, C_2H_5D, H_2, D_2, HD, benzene and toluene reaching steady state, the reactor was raised from 50 to 800°C with a heating rate of 10°C/min, and the above selected MS signals were recorded.

C_2H_6-TPSR over deuterium-labelled catalysts: The catalysts were pre-treated in 30 mL/min 5 vol% D_2 (balanced with N_2) at 600°C for 1 h. Then, the reactor was cooled down to 50°C, the flow was switched to a 30 mL/min 25 vol% C_2H_6/N_2. After the selected MS signals including CO, CO_2, C_2H_4, C_2H_5D, H_2, D_2, HD, benzene and toluene reaching steady state, the reactor was raised from 50 to 800°C with a heating rate of 10°C/min, and the above selected MS signals were recorded.

OH-FTIR: The catalysts were pre-treated in N_2 at 600°C for 1 h. Then, the OH-FTIR spectra at desired temperatures were collected in N_2 atmosphere. A 10 vol% CO/N_2 was used for obtaining the OH-FTIR spectra under CO-treatment.

Pyridine and CD_3CN-FTIR: The catalysts were pre-treated in 20 mL/min N_2 at 600°C for 1 h. Then, the catalysts were cooled to desired temperatures (i.e., 200 and 350°C for pyridine-FTIR; 30, 100 and 200°C for CD_3CN-FTIR) and collected the backgrounds at these temperatures. Afterwards, the catalysts were cooled to 50°C and saturated with pyridine or CD_3CN-FTIR. Subsequently, the catalysts were heated to desired temperatures in 20 mL/min N_2 flow, and maintained for 30 min to desorb gaseous or physically adsorbed pyridine and CD_3CN molecules, and then the corresponding FTIR spectra were recorded.

C_2H_6-FTIR: The catalysts were pre-treated in N_2 at 600°C for 1 h, and then, the IR cell

was cooled to 400°C to collect the background. Afterward, the flow was switched to 20 mL/min 5 vol% C_2H_6/N_2, and the C_2H_6-FTIR spectra (simulating EDH reaction) were recorded.

***In-situ* XAS:** As illustrated in Figure S21, *in-situ* XAS spectra were recorded for 0.50Co-Z5(340) under various treatment. Initially, the catalyst was pre-treated in He at 600°C for 1 h to acquire the spectrum under He at 600°C. The XAS cell was then cooled down to 25°C under He flow, and the gas was switched to 10 vol% CO/He. After CO-treatment at 600°C for 2 h, the cell was purged with He. Subsequently, the flow was switched to 40 vol% C_2H_6/He and maintained at 600°C for 2 h to collect the XAS spectra of CO&EDH treatment at 600°C. Then, the cell was cooled to 25°C under He flow, and the XAS spectra of CO&EDH treatment at 25°C were collected. In a separate experiment, another piece of fresh 0.50Co-Z5(340) was pretreated in He at 600°C for 1 h. The flow was then switched to 40 vol% C_2H_6/He and kept at 600°C for 2h to collect the XAS spectra under direct EDH treatment at 600°C. Subsequently, the XAS cell was cooled to 25°C under He flow, and the XAS spectra of EDH treatment at 25°C were collected.

EXAFS fitting: The data processing was carried out utilizing the IFEFFIT package[1]. According to the Nyquist theorem and using the k and R ranges presented in Table 1 and Table S2, we obtain a (minimum) number of free parameters of 14 for three initial (fresh) catalysts and 0.50Co-Z5(340) upon CO&EDH treatment, and free parameter of 21 for 0.50Co-Z5(340) after direct EDH treatment[2,3]. Thus, the high k and R ranges available for fitting (Table 1 and Table S2) allow to fit up to 3 shells for the fresh and CO&EDH catalysts and 5 shells for the EDH case. Phase and amplitude functions for the Co–O, Co–Si (Co–O–Si), Co–Al (Co–O–Al), and Co–Co were obtained using FEFF[4].

Supporting theoretical calculation methods

In metadynamics (MTD) simulations, the distance and coordination number of selective atoms were set as collective variables. The coordination number is defined as:

$$CN = \sum_{ab} \frac{1 - (r_{ab}/r_0)^{nn}}{1 - (r_{ab}/r_0)^{nd}}$$

Here, r_{ab} represents the distance between atoms *a* and *b*. *nn*, and *nd* are set to 6 and 12, respectively. r_0 corresponds to a reference bond distance, and takes 1.60 Å for the CV(C–H) and CV (H–H) and 2.00 Å for other collective variables. The collective variables for various MTD simulations were given in Table S4.

In addition, quadratic walls are set as WALL_MINUS with position of r_0(Co–C) = 4 Å and Ka = 50 kJ/mol for MTD simulations about C_2H_6* activation, which restrict ethane diffuse away cobalt sites.

The spin-polarized density function theory (DFT) calculations were conducted for static transition state searches (confirming the accuracy of the reaction pathways explored using MTD) and electronic analysis. The DFT calculations were implemented in VASP 5.4.4 package with using the GGA-PBE electron exchange-correlation function. The grimme's D3 method was employed to correct the van der Waals dispersion interactions in all calculations. Brillouin zone sampling was Gamma point. The cutoff energy and force criterion was set as 400 eV and 0.05 eV/Å, respectively. The static transition state searches were conducted using the combined nudged elastic band (CI-NEB)[5] and improved dimer method (IDM)[6], and were confirmed by only one imaginary vibrational frequency along the reaction coordinate. In present study, three distinct reaction paths for ethane C–H cleavage were explored by static transition state searches (Figure S39), and the results confirm the accuracy of the reaction pathways explored using MTD.

On the other hand, Bader charge analysis was employed to evaluate the electronic density on cobalt sites during activating ethane C–H bonds. Crystal orbital Hamilton populations (COHP) analysis quantitatively evaluate the binding strength between active sites (Co or Co–O) and reactions intermediates, which were implemented within LOBSTER program[7].

Supporting Figures

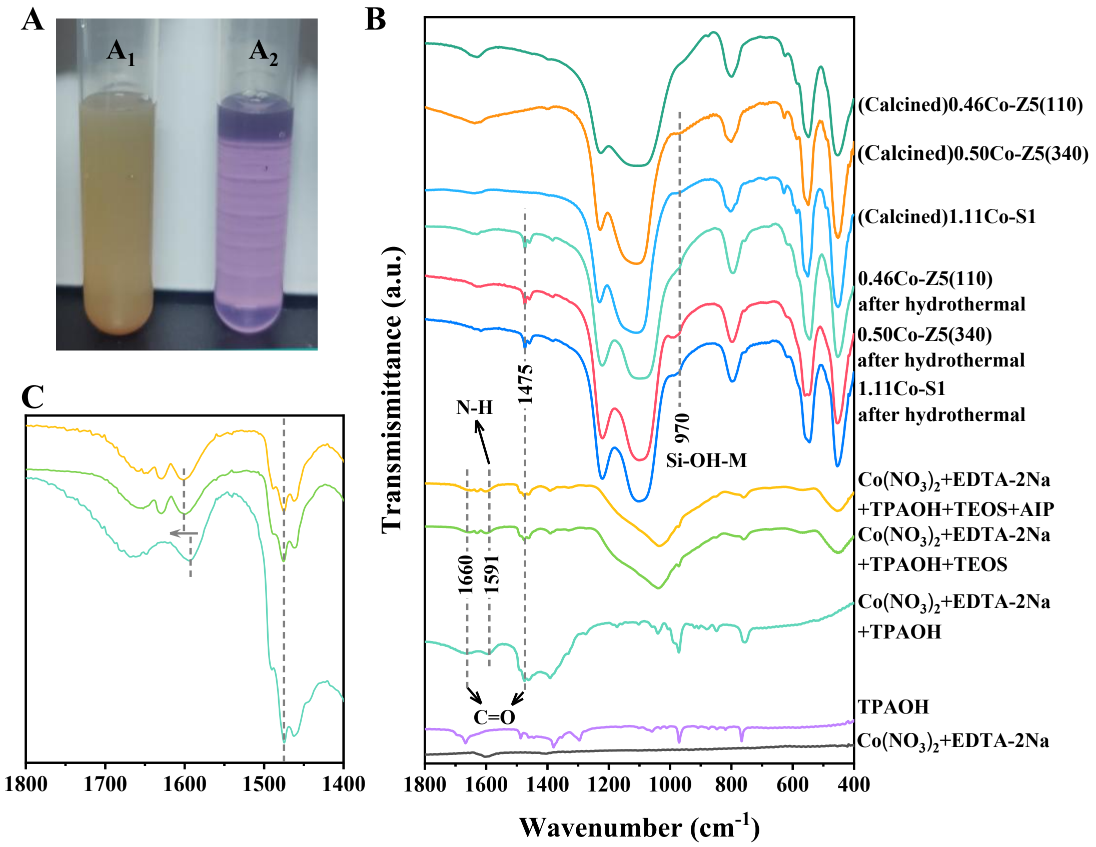

Figure S1. (A) Photo images of hydrothermal precursor of A_1: $Co(NO_3)_2$+TPAOH and A_2: Co-EDTA-2Na+TPAOH; (B) transmission FTIR spectra of synthesis precursors at different stages; (C) enlarged picture of panel B within 1400-1800 cm^{-1} region.

Note: From Figure S1A, one can see the use of Co-EDTA-2Na prevents the precipitation of as cobalt species (A_2), while the use of $Co(NO_3)_2$ as precursor results in obvious precipitation (A_1). In the FTIR spectra of precursor at different stages (Figure S1B,C), the 1591 cm^{-1} bands slightly shift after TEOS addition, which may be attributed to the interaction of Co-EDTA complex and silica precursor.

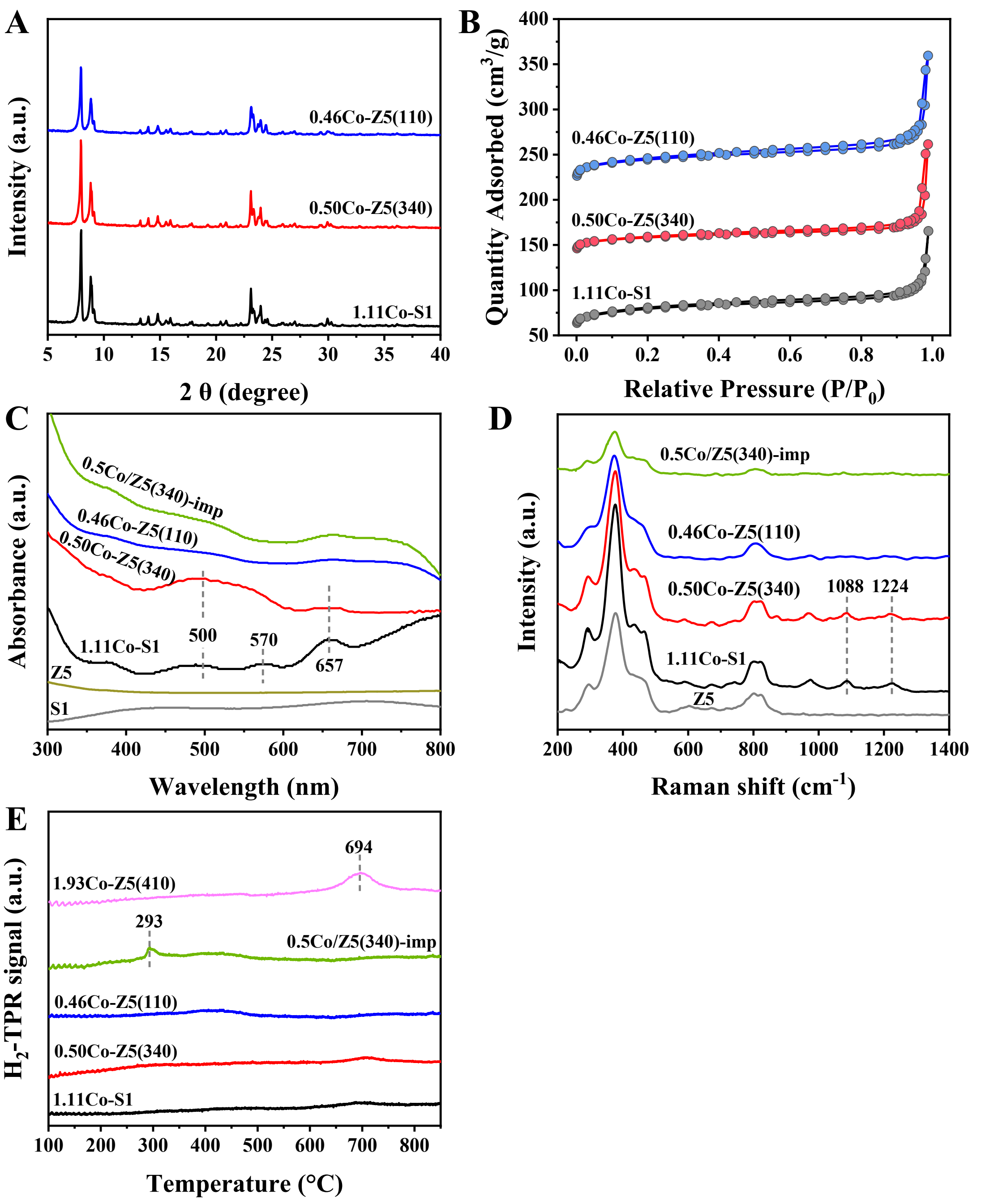

Figure S2. (A) XRD patterns; (B) N_2 adsorption-desorption isotherms; (C) UV-Vis DR and (D) Raman spectra excited by 325 nm laser; (E) H_2-TPR profiles.

Note: As shown in Figure S2E, the low-temperature TPR peak around 295°C was assigned to superficial CoO_x nanoparticles in Co/Z5-imp catalyst. As reference, the H_2-TPR peak around 694°C was attributed to the encapsulated CoO clusters within MFI channels, for 4Co-Z5. In contrast, these TPR peaks are not observed for other catalysts, which suggests the absence of CoO or CoO_x clusters/nanoparticles.

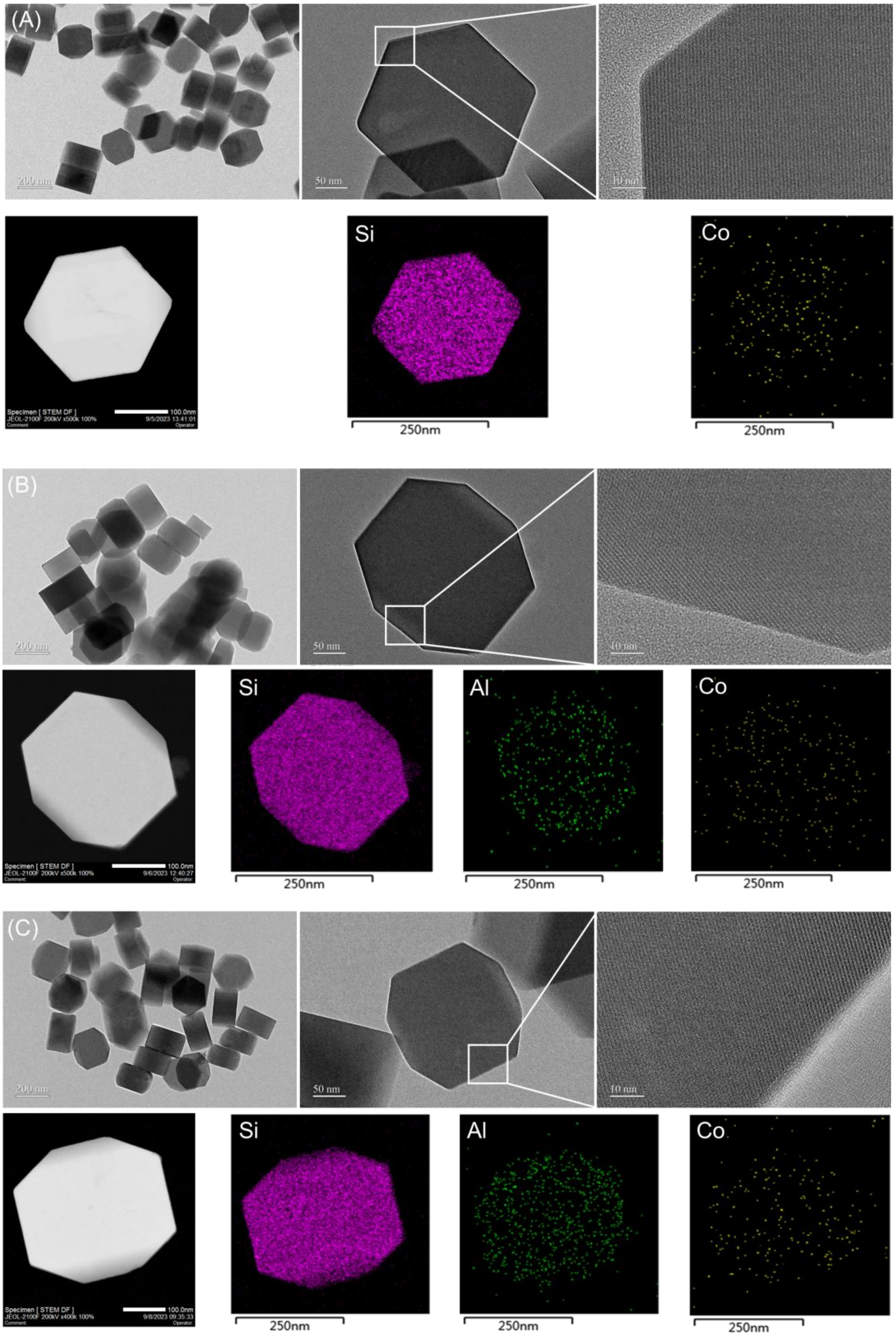

Figure S3. TEM and elemental mapping images of various catalysts. (A) 1.11Co-S1; (B) 0.50Co-Z5(340) and (C) 0.46Co-Z5(110).

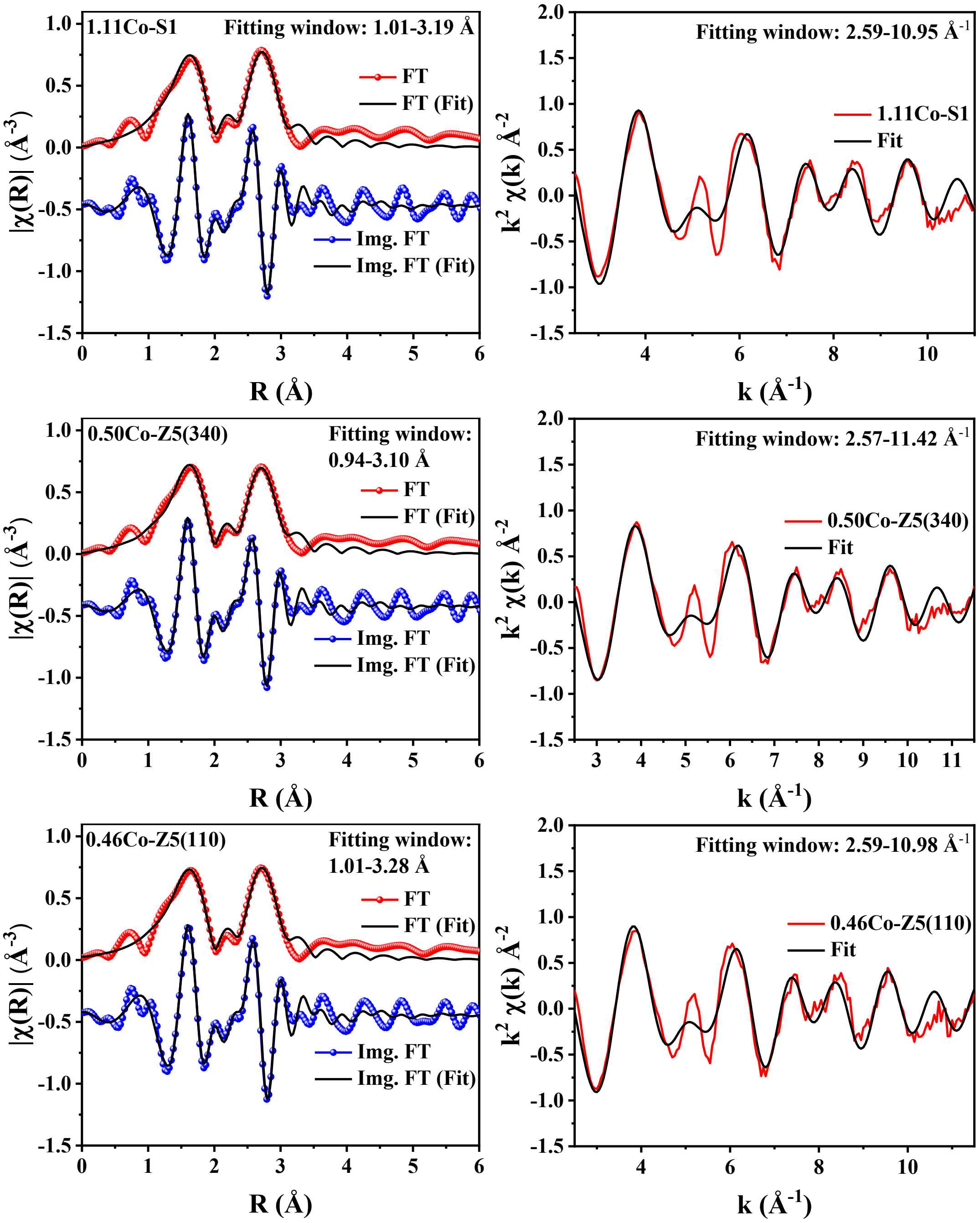

Figure S4. EXAFS fitting results for various fresh samples; see fitting parameters in Table S2.

A

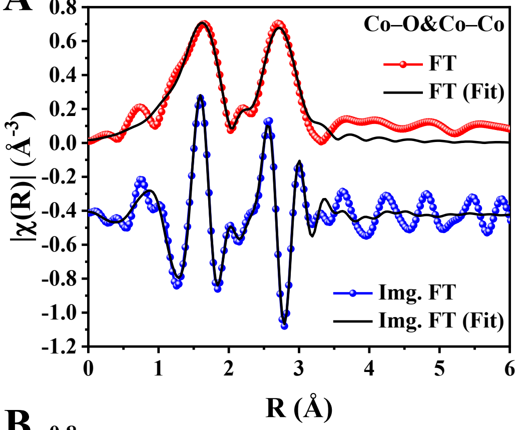

Shell	CN [a]	R/ Å [b]	$\Delta\sigma^2$ / Å^2 [c]	E_0 /eV [d]
Co–O	2.6	2.01	0.0065	2.67
Co–Co	5.33	3.33	0.0095	-23.23

B

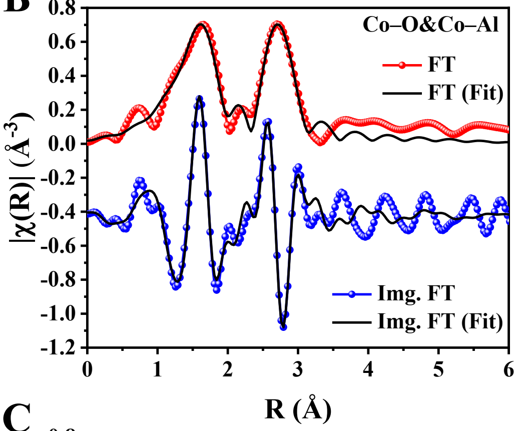

Shell	CN [a]	R/ Å [b]	$\Delta\sigma^2$ / Å^2 [c]	E_0 /eV [d]
Co–O	2.3	2.07	0.0045	2.07
Co–Si	2.8	3.24	0.00013	-2.79
Co–Al	0.14	2.77	0.00096	-17.9

C

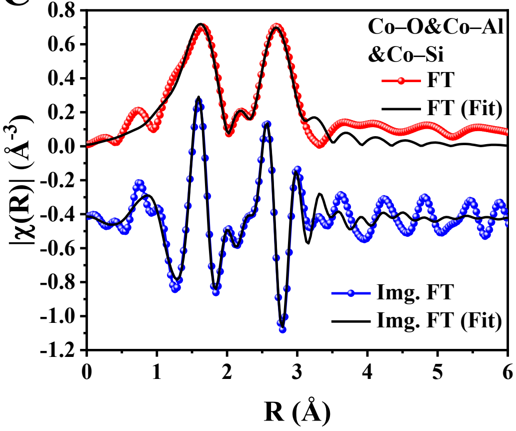

Shell	CN [a]	R/ Å [b]	$\Delta\sigma^2$ / Å^2 [c]	E_0 /eV [d]
Co–O	2.49	2.06	0.008	-0.36
Co–Al	-1.2	2.90	-0.001	-33.15

Figure S5. EXAFS fitting results of fresh 0.50Co-Z5(340) sample with using various second scattering paths; [a] CN, coordination number; [b] R, bonding distance; [c] $\Delta\sigma^2$, Debye-Waller, [d] E_0, inner potential shift; average standard error for above parameters is R, 0.01 Å; CN, 9.2 %; $\Delta\sigma^2$, 10.5 %; E_o 0.4 eV.

Note: the attempts using Co–O & Co–Co, Co–O & Co–Si & Co-Al, and Co–O & Co–Al scattering paths show unreasonable parameters (e.g., E_0<-10; CN(Co–Al) < 0) or meaninless parameters (1st CN(Co-Co)= 5.33 and lack of higher shell(s) signals).

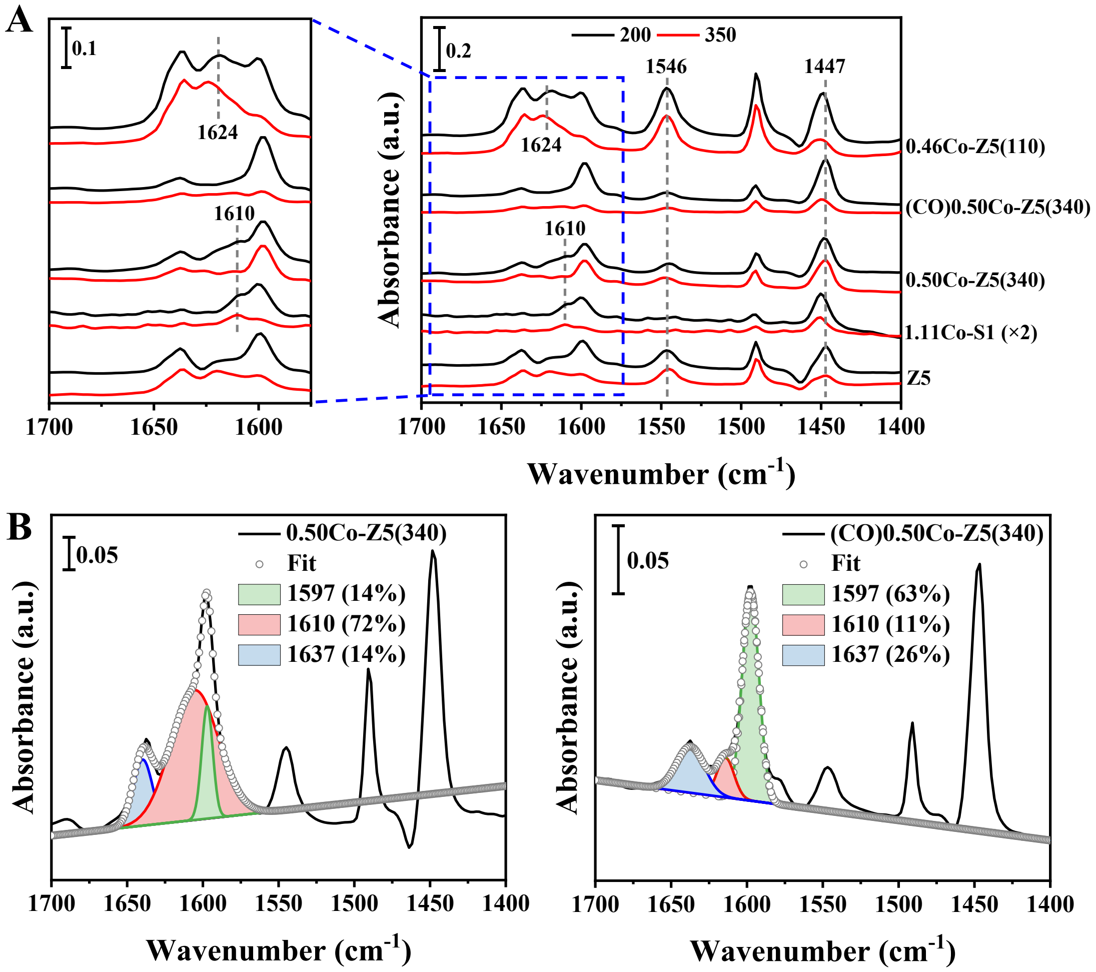

Figure S6. (A) Transmission FTIR spectra with pyridine and the enlarged picture within 1700-1575 cm^{-1}; (B) spectra deconvolution results within 1650-1675 cm^{-1} for 0.50Co-Z5(340) and (CO)0.50Co-Z5(340).

Note: In Figure S6A, the pyridine-FTIR bands at 1447 and 1610/1624 cm^{-1} belong to LAS, while the 1546 cm^{-1} bands were attributed to BAS.[8,11] As the 1610/1624 cm^{-1} bands are absent in· Z5 support, they are attributed to different Co sites in Co-MFI samples. The 1610 cm^{-1} bands can be assigned to isolated tetrahedral Co^{2+} species, likely locate at framework position.[8,11] In addition, the 1624 cm^{-1} bands were attributed to pyridine interacting with exchange Co^{2+} species. The spectra deconvolution results in Figure S6B indicate the relative area proportion of the characteristic 1610 cm^{-1} band (due to pyridine coordinated to framework Co^{2+}) decreases from 72% in 0.50Co-Z5(340) to 11% in (CO)0.50Co-Z5(340). This supports the transformation of framework Co^{2+} species during CO treatment, as confirmed by *in-situ* XAS analysis.

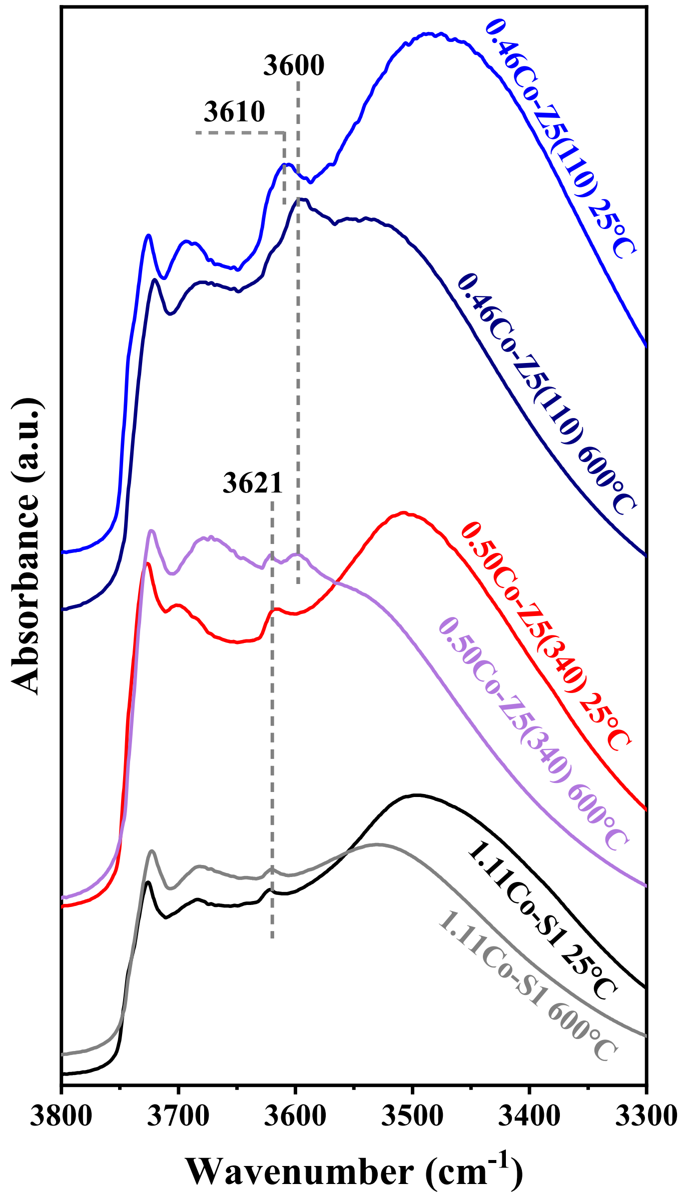

Figure S7. Transmission FTIR spectra within hydroxyl region of catalysts in N_2 atmosphere under different temperatures.

Note: In 0.46Co-Z5(110), the 3610 (at 25°C) and 3600 cm^{-1} (at 600°C) bands both correspond to Si–OH–Al species, while 1.11Co-S1 shows only Si–OH–Co bands at 3621 cm^{-1}. Therefore, the temperature-dependent evolution of Si–OH–Al bands in 0.50Co-Z5(340) was absent for 1.11Co-S1 and 0.46Co-Z5(110).

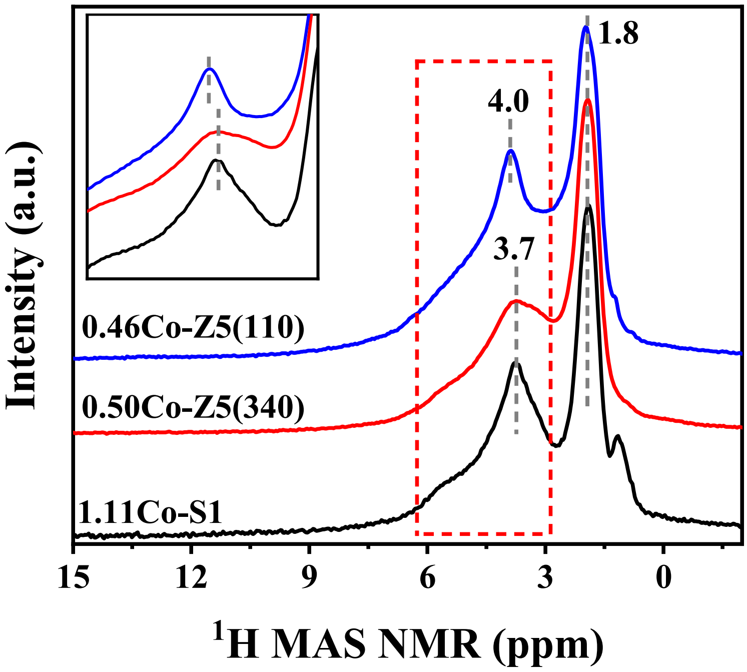

Figure S8. ^{1}H NMR spectra for various catalysts.

Note: The signals at 1.8 and 4.0 ppm correspond to protons of external Si–OH groups and Si–OH–Al groups, respectively.[12] The signals at 3.7 ppm can be assigned to geminal Si–$(OH)_2$ groups,[13] which may associated with the Si–$(OH)_2$–Co^{2+} species, that is framework Co^{2+} sites within MFI zeolite crystals.

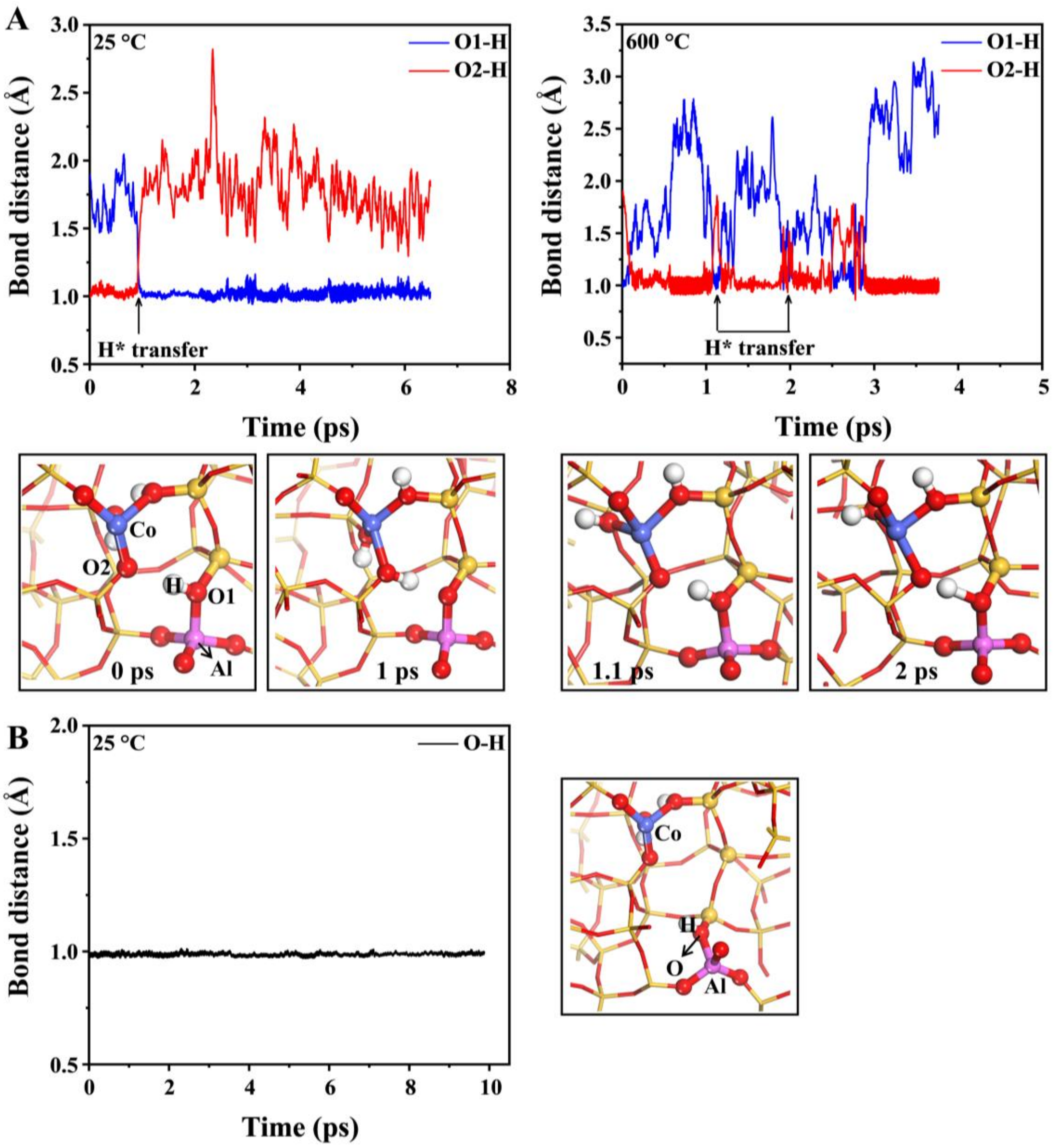

Figure S9. The variations of the bond distances for the inferred 0.50Co-Z5(340) models during AIMD simulations at different temperatures: (A) NNNN structure with Co–(O–Si)$_2$–O–Al linkage; (B) Co–(O–Si)$_3$–O–Al linkage; purple, red, yellow, pink and white balls represent Co, O, Si, Al and H atoms.

Note: The AIMD simulations based on configuration with Co–(O–Si)$_2$–O–Al (next-next-nearest neighbor) can enable temperature-dependent proton transfer as reflected by bond distance evolution, but it is less stable than Co–O–Si–O–Al structure (see below Figure S10). For comparison, the Co–(O–Si)$_3$–O–Al model shows no proton transfer.

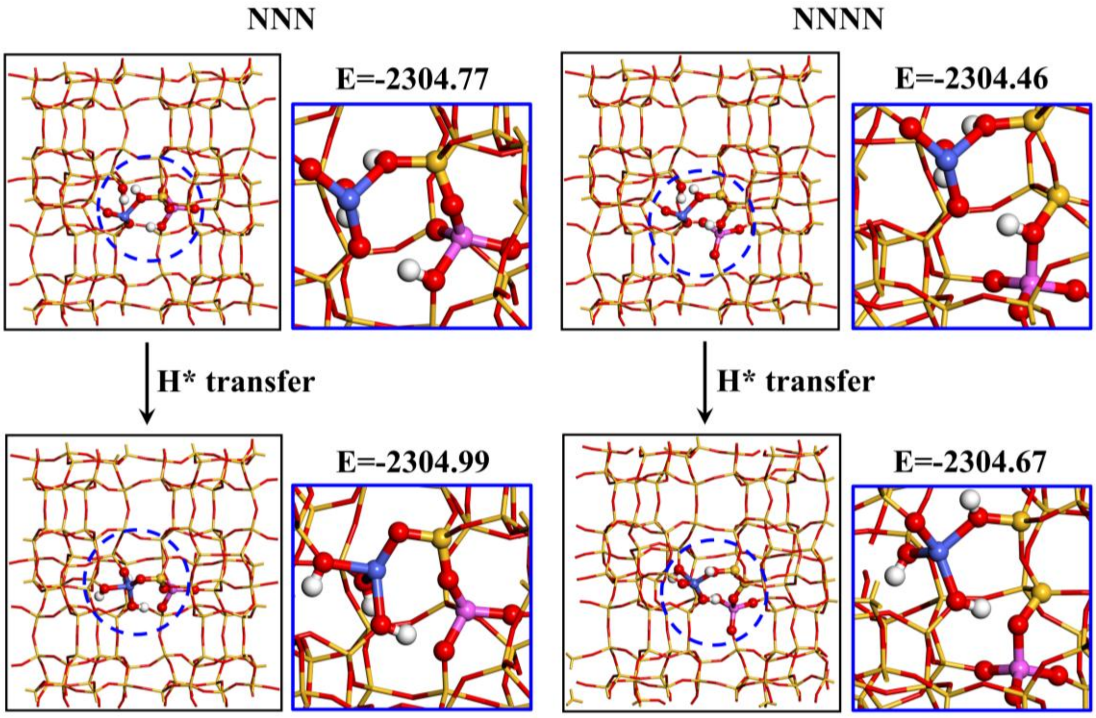

Figure S10. The DFT-calculated energies for possible 0.50Co-Z5(340) structure with NNN or NNNN coordination; purple, pink, yellow, red, grey, white balls represent Co, Al, Si, O, C, H atoms.

Note: The configuration of framework Co^{2+} with NNN-BAS configuration (Co–O–Si–O–Al linkage) is energetically favorable compared to the counterpart with NNNN-BAS (Co–$(O–Si)_2$–O–Al linkage).

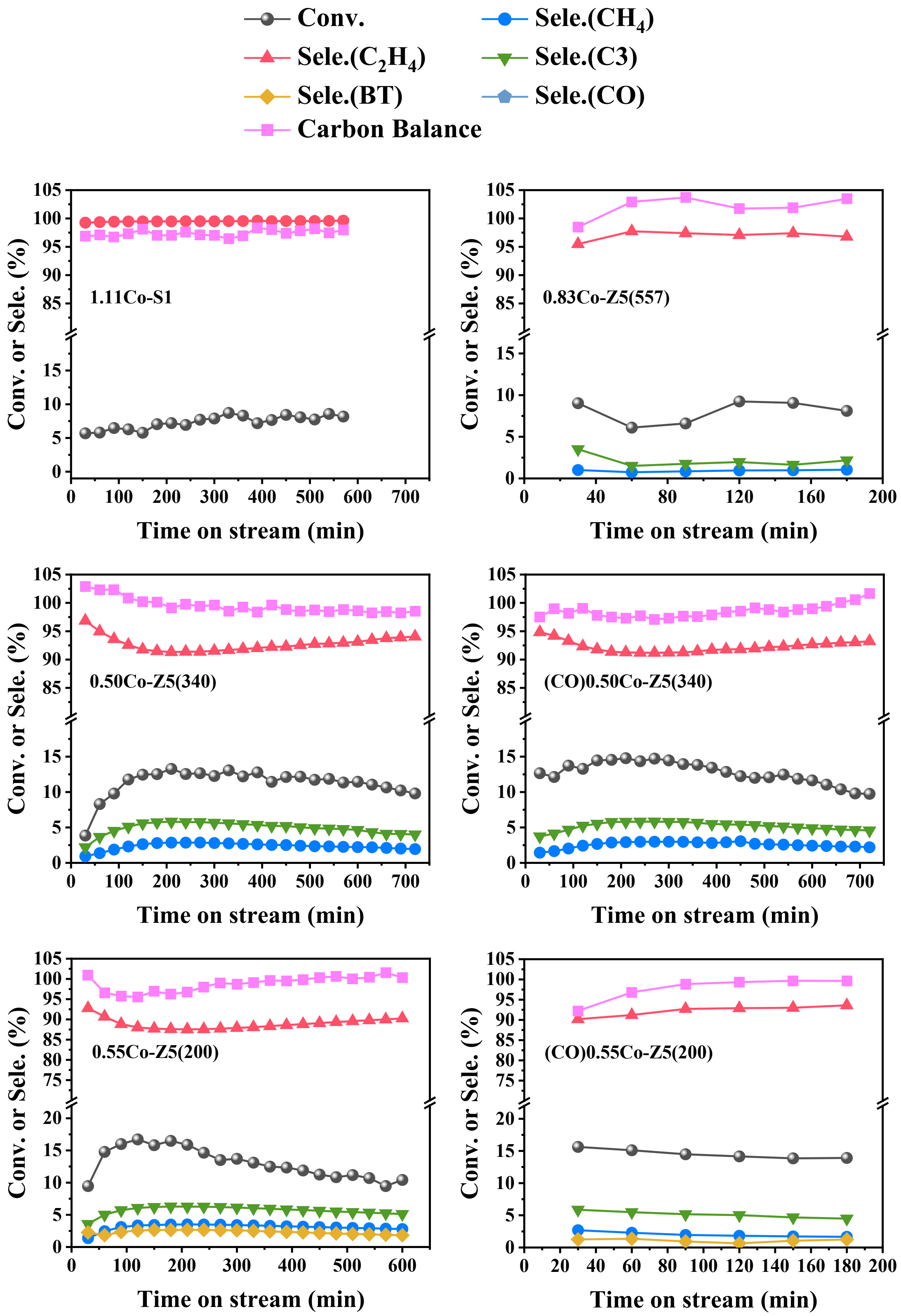

Figure S11. Catalytic EDH performance over various Co-MFI samples; reaction conditions are 0.2 g catalyst, 600°C, WHSV=4.73 h^{-1}, 80 vol% C_2H_6.

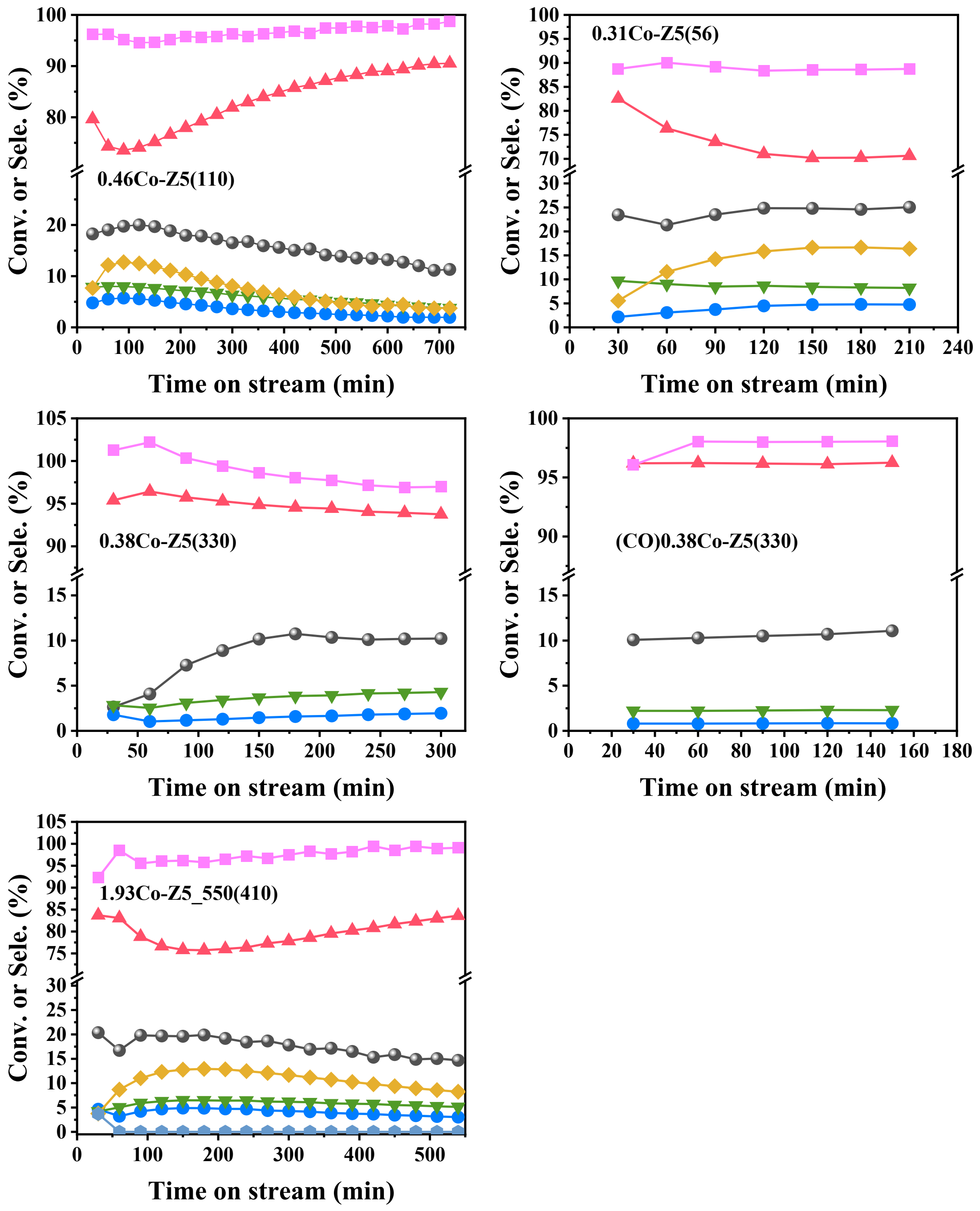

Continued Figure S11. Catalytic EDH performance over various Co-MFI samples; reaction conditions are 0.2 g catalyst, 600°C, WHSV=4.73 h^{-1}, 80 vol% C_2H_6.

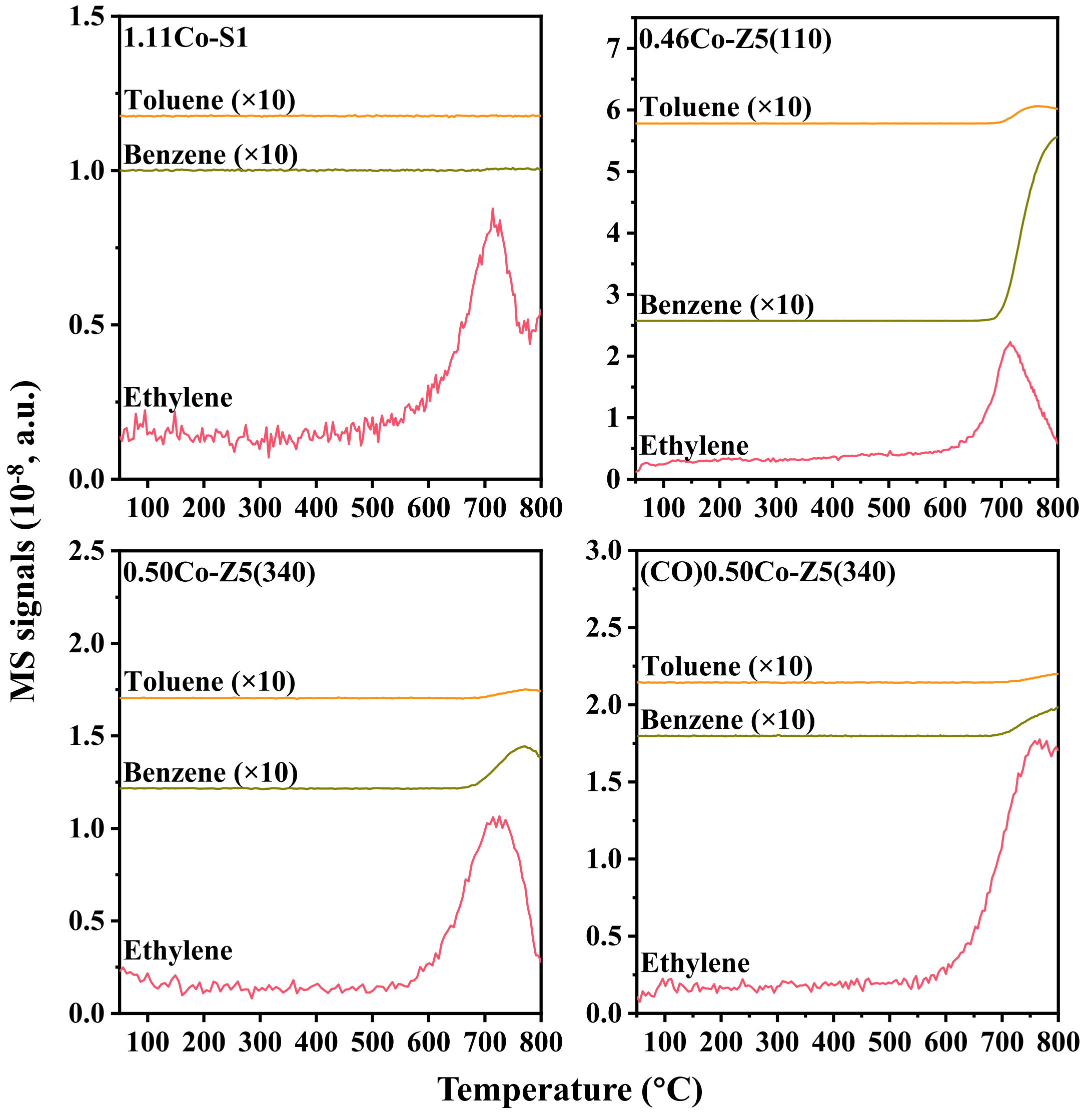

Figure S12. MS signals of ethylene, benzene and toluene formation during C_2H_6-TPSR measurements.

Note: 0.46Co-Z5(110) shows stronger benzene formation signals compared to other tested catalysts, which is consistent with its higher selectivity to aromatics products (compared to 1.11Co-S1 and 0.50Co-Z5(340)) in activity evaluation.

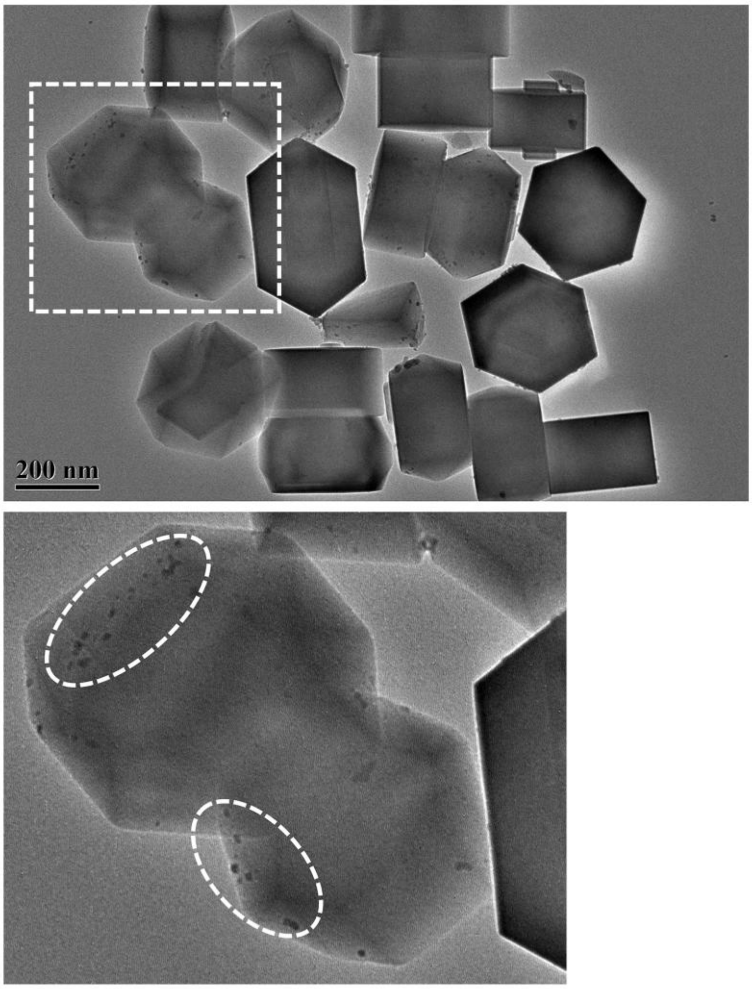

Figure S13. TEM images of impregnated 0.5Co/Z5(340)-imp catalyst, below shows the enlarged image of white boxed region in upper image.

Note: The white circles in below image indicate there are CoO_x nanoparticles (about 3~5 nm) in 0.5Co/Z5(340)-imp catalyst. These CoO_x nanoparticles were absent for catalysts synthesized via one-pot hydrothermal method (Figure S3).

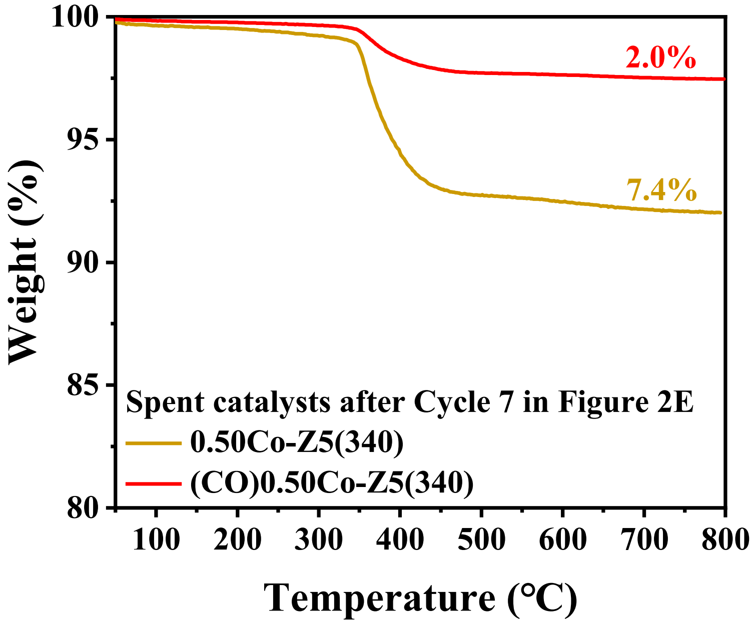

Figure S14. TG profiles for spent catalysts after Cycle 7 in Figure 2E.

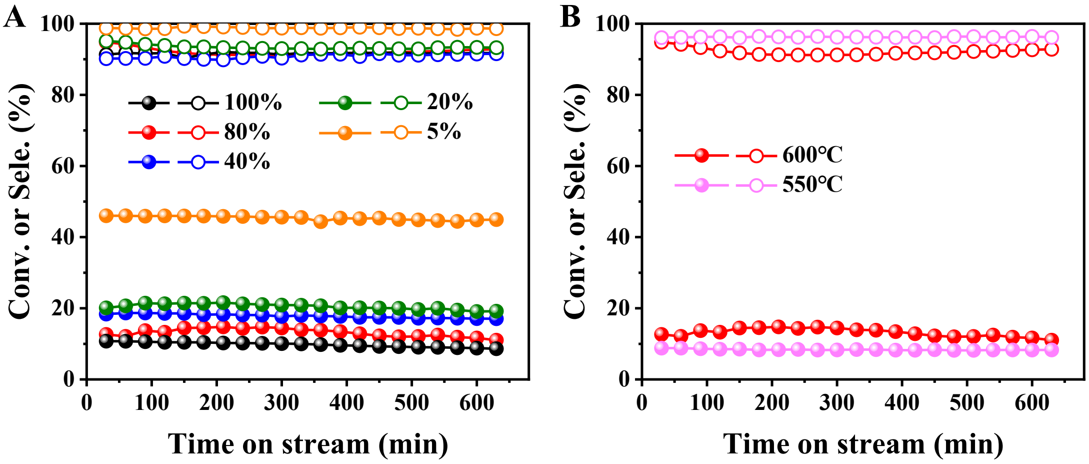

Figure S15. Catalytic performance of (CO)0.50Co-Z5(340) under different feeds or temperatures; the detailed reaction conditions and activity data are summarized in Table S3.

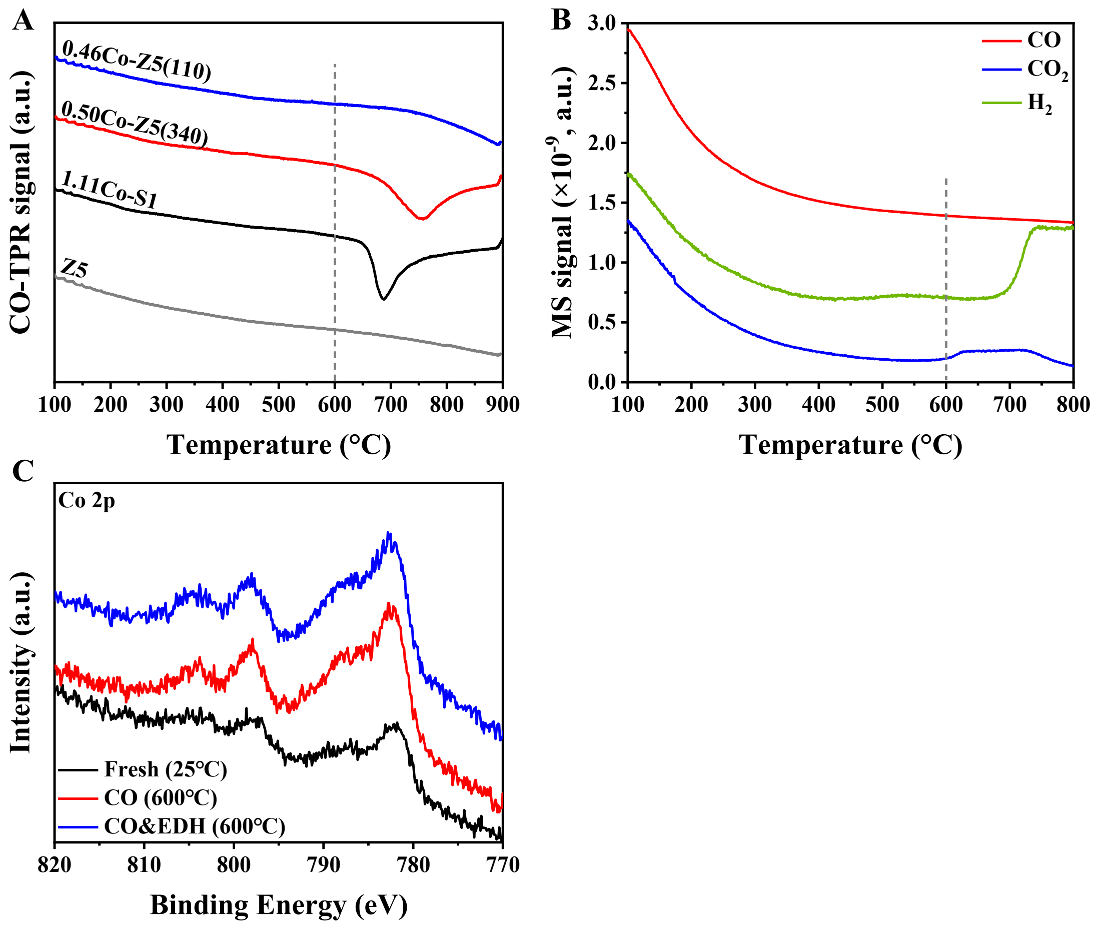

Figure S16. (a) CO-TPR profiles for various samples; (b) CO-TPSR MS profiles of 0.50Co-Z5(340); (c) *in-situ* Co 2p XPS spectra for 0.50Co-Z5(340) during various treatment.

Note: Figure S16A, B show the negiable CO-TPR and CO_2/H_2 evolution MS signaols below 600°C, indicating the cobalt species cannot be reduced below 600°C.

Figure S16C shows the *in-situ* Co 2p XPS spectra. Upon CO treatement (CO catalyst), and subsequent EDH reaction (CO&EDH case), there is no 2p3/2 XPS peaks associated with metallic Co (about 778.0 eV) and cobalt carbide (about 776.6 eV),[14] again confirming the retention of cobalt oxidation state in (CO)0.50Co-Z5(340).

If there is reverse water gas shift reaction between Si–OH–Co species and CO (e.g., Si–OH–Co + CO → Si–Co + CO_2 + $0.5H_2$), one can expect the evolution of CO_2/H_2 signals together with the reduction of cobalt sites, which is not consistent with the observations in 0.50Co-Z5(340).

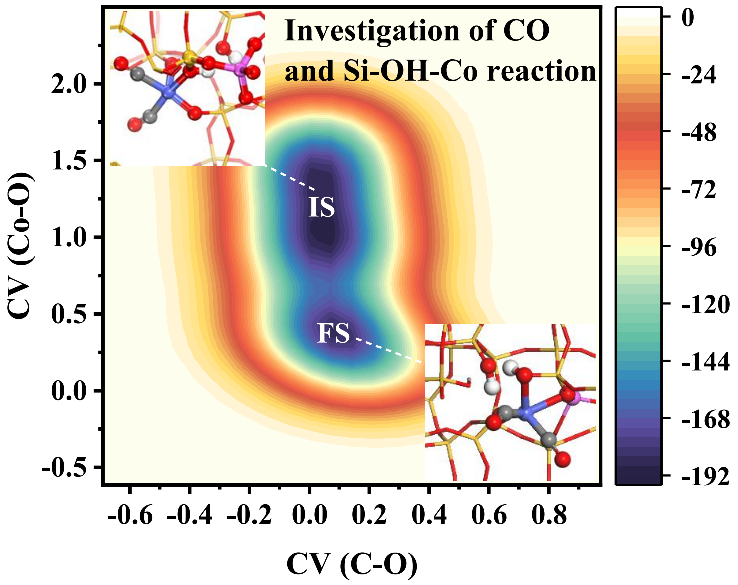

Figure S17. MTD simulations of reverse water gas shift reaction between Si-OH-Co and CO molecules; purple, pink, yellow, red, grey, white balls represent Co, Al, Si, O, C, H atoms.

Note: In this case, we set coordination number of C-O (CV(C-O), C from CO molecule and O from Si-O(H)-Co species) and coordination number of Co-O (CV, Co atom and its coordinated O atoms) as collective variables. If there is reverse water gas shift reaction between Si-OH-Co and CO, one can expect the increase in CV (C-O) (i.e., formation CO_2) together with decrease in CV (Co-O). For this case, the CV (C-O) maintain almost unchanged and the reverse water gas shift reaction did not occur throughout the MTD trajectories.

In contrast, we observed the approach of cobalt towards nearby Al site (final state (FS) in inset of Figure S17), which inspires us to conduct the further MTD simulations as shown in Figure 3E.

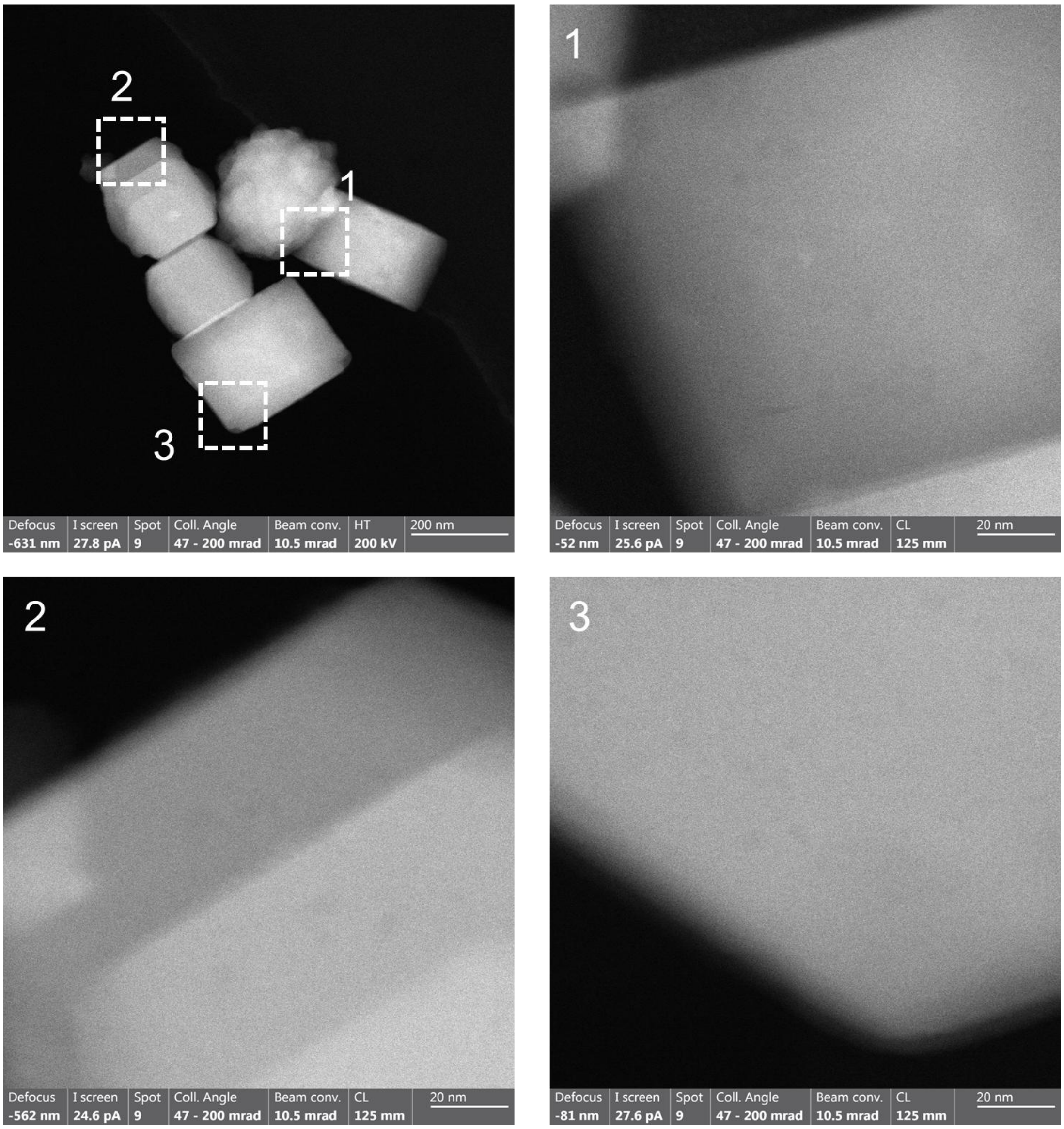

Figure S18. STEM images of the spent (CO)0.50Co-Z5(340).

Note: There are no detectable CoO_x nanoparticles in regenerated (CO)0.50Co-Z5(340) (i.e., after CO&EDH treatment and air regeneration), which rule out the sintering of cobalt species. Also, this supports the absence of reduction of cobalt sites towards metallic Co clusters during CO treatment.

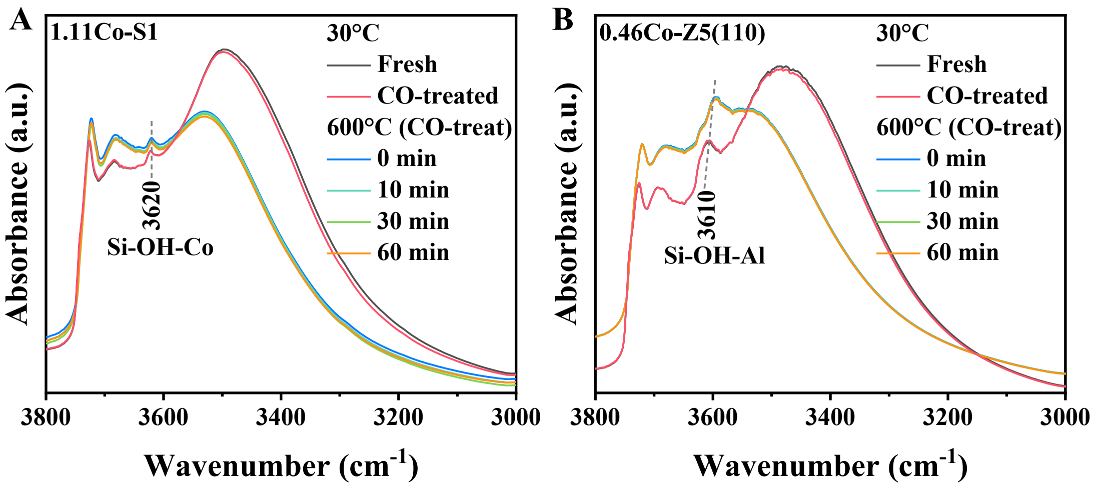

Figure S19. *In-situ* transmission FTIR spectra within hydroxyl region during CO treatment for (A) 1.11Co-S1 and (B) 0.46Co-Z5(110).

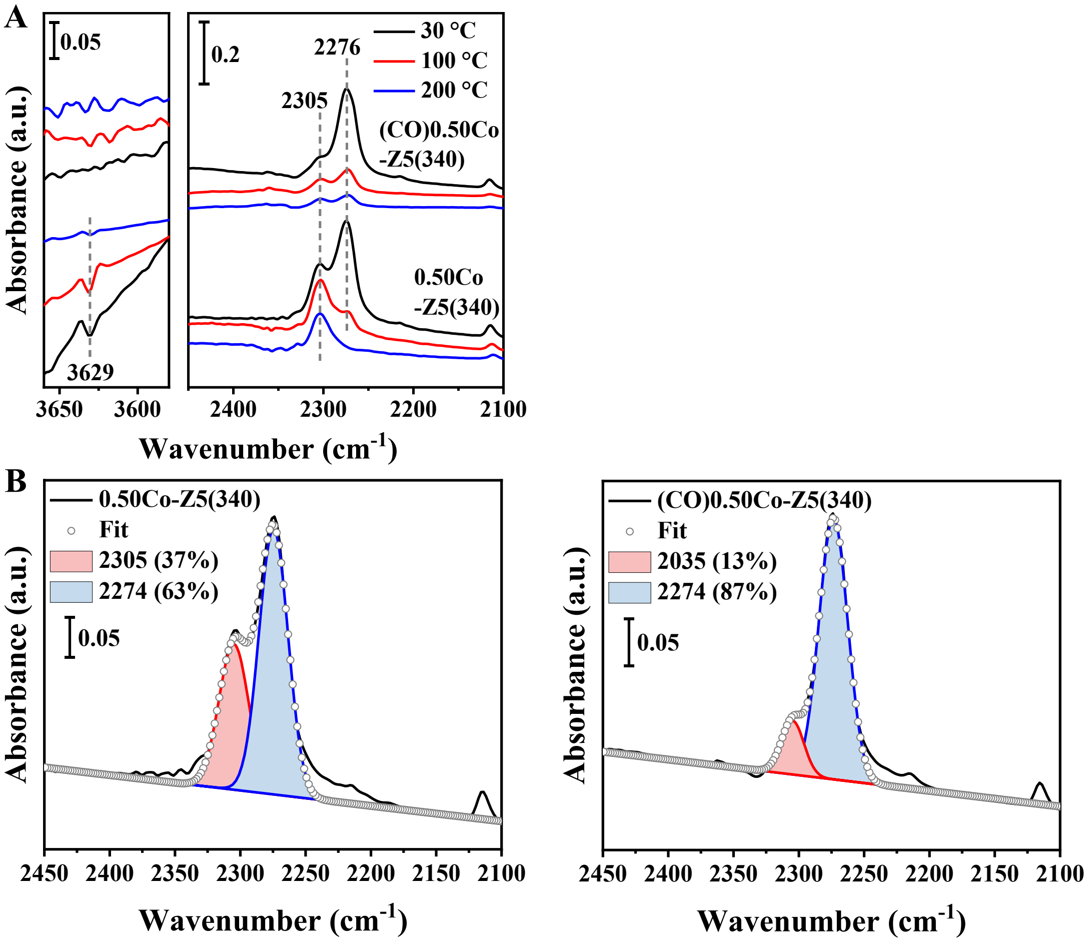

Figure S20. (A) Transmission FTIR spectra with CD_3CN of fresh and CO pre-treated 0.50Co-Z5(340) samples; (B) spectra deconvolution results within 1650-1675 cm^{-1}.

Note: The 2305 cm^{-1} band, assigning to CD_3CN coordinated to framework Co^{2+}, decreases in (CO)0.50Co-Z5(340) compared to fresh one. Moreover, the spectra deconvolution results in Figure S20B indicate the relative area proportion of the characteristic 2305 cm^{-1} band decreases from 37% in 0.50Co-Z5(340) to 13% in (CO)0.50Co-Z5(340). This again suggests the CO treatment results in transformation of framework Co^{2+} species

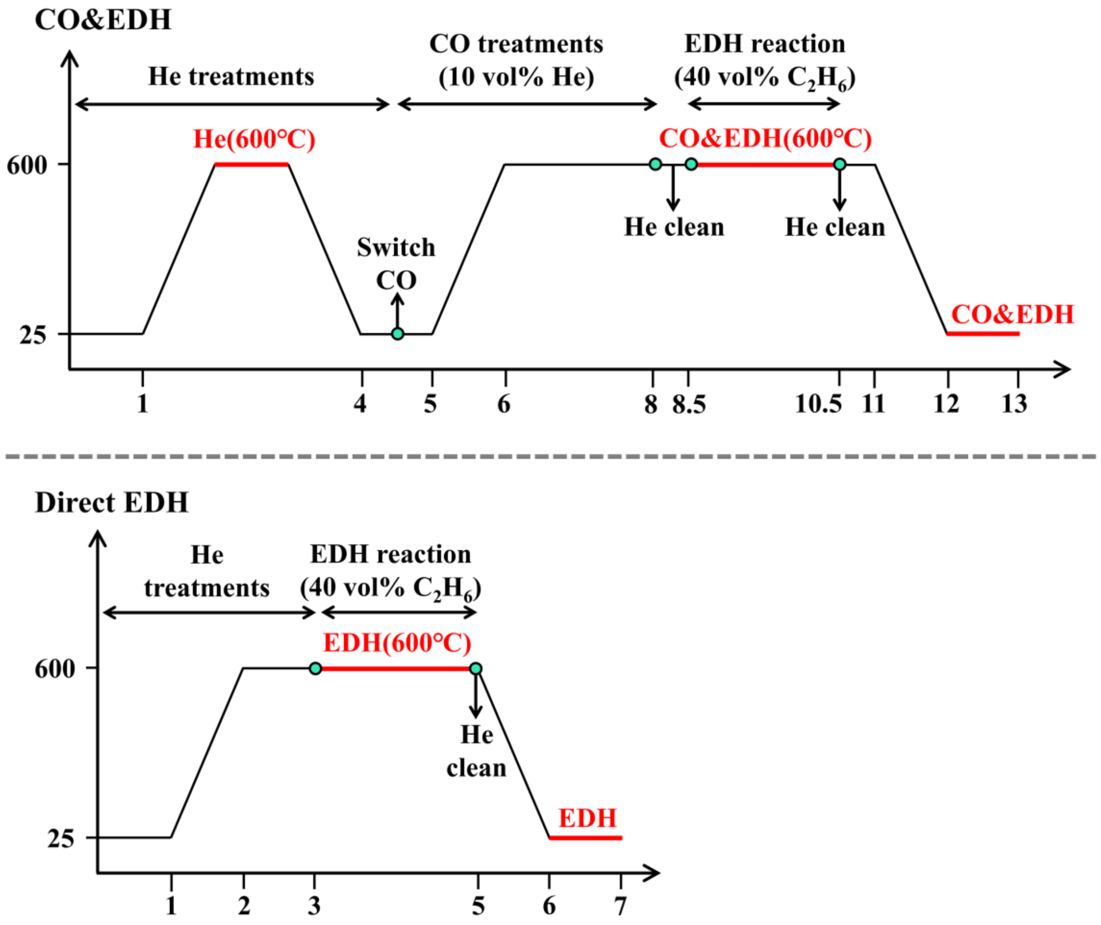

Figure S21. Schematic diagram for collecting the *in-situ* XAS spectra during various treatment; the red text indicate different spectra in Figure 3 & S22.

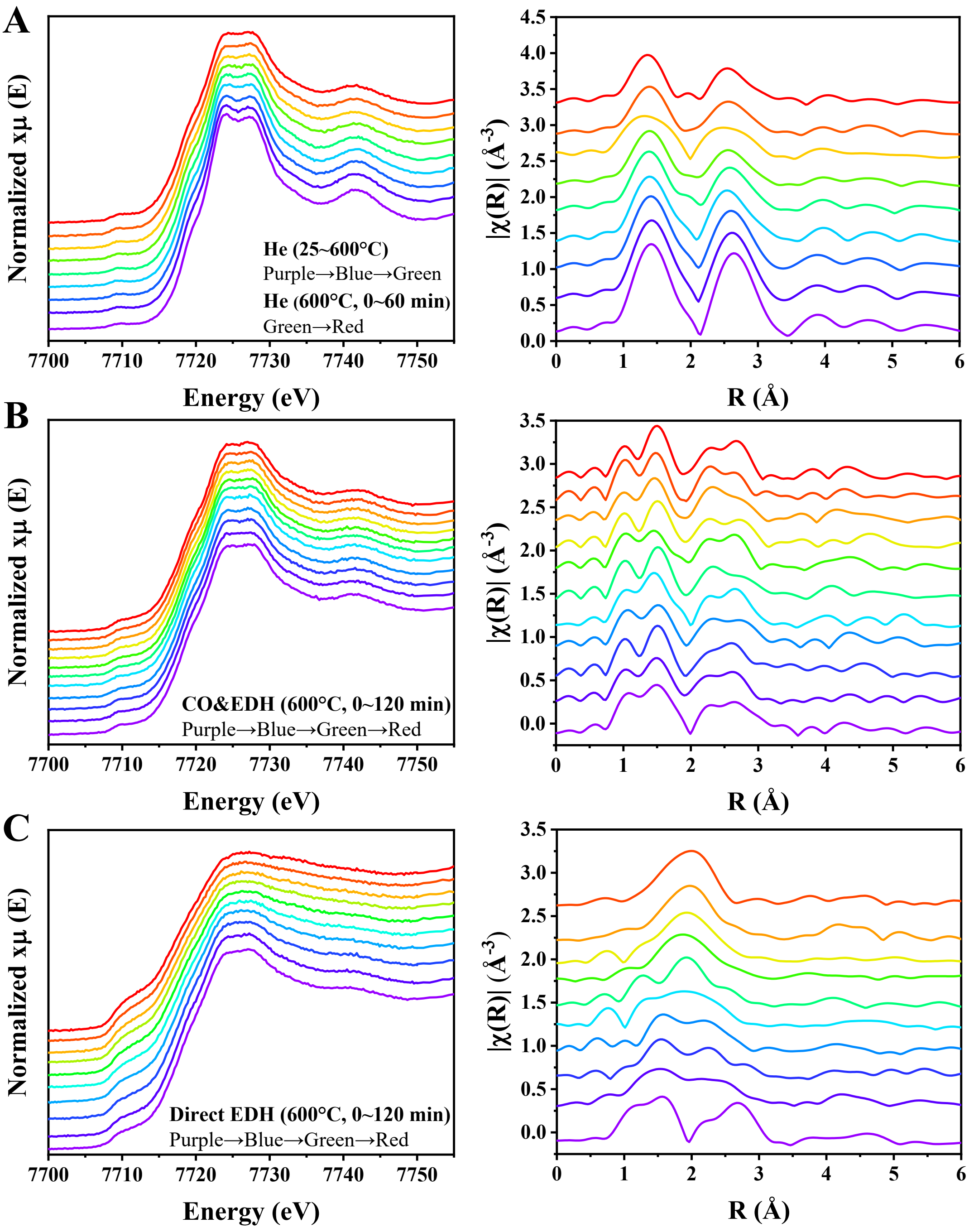

Figure S22. *In-situ* Co K-edge XANES and corresponding FT-EXAFS spectra of 0.50Co-Z5(340) during (A) He tratement; (B) CO&EDH treatment and (C) direct EDH treatment.

Note: The EXAFS spectra in Figure S22A indicates a Co–Al coordination at about 2.73 Å (with phase shift correction) for CO&EDH catalyst at 600°C. These individual ten scans were merged and calibrated to obtain the XAS spectrum of CO&EDH (600°C) in Figure 3C, which was used for further EXAFS fitting.

In contrast, there is predominant Co-Co scattering peaks at about 2.51 Å (with phase shift correction) for direct EDH at 600°C, which is consistent with the results after colling down to room temperature (EDH spectrum in Figure 3C).

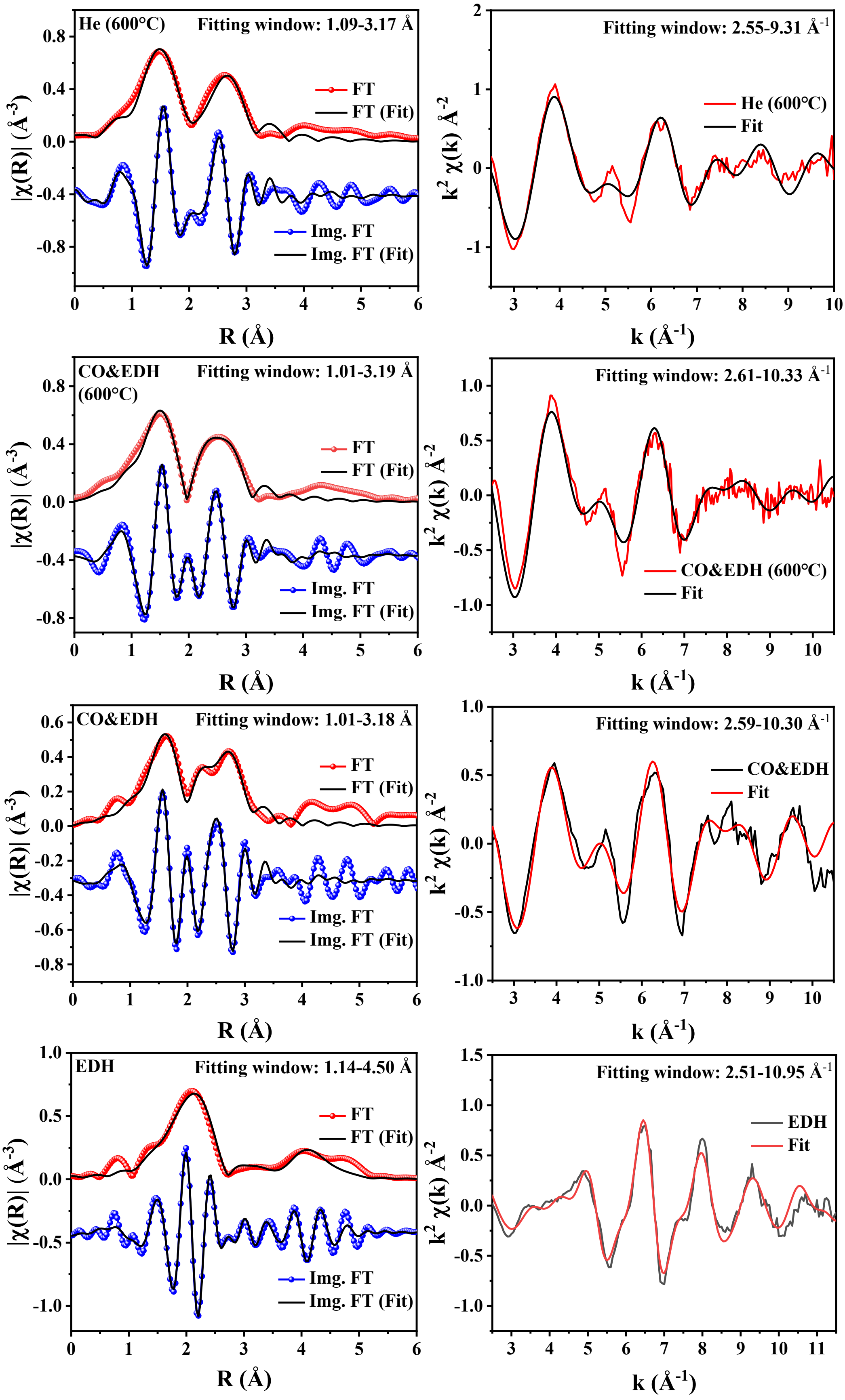

Figure S23. EXAFS fitting results of (CO)0.50Co-Z5(340) under different treatment; see fitting parameters in Table 1.

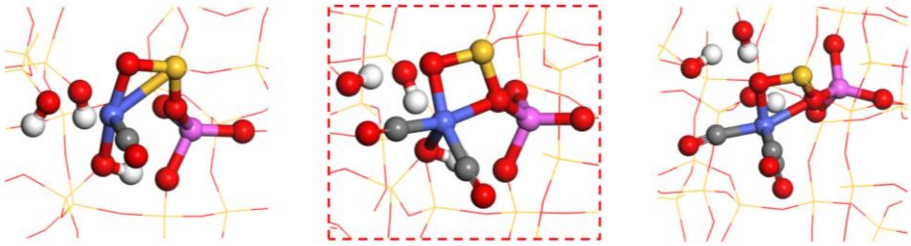

Structure	Co(CO)	$Co(CO)_2$	$Co(CO)_3$
ΔE_{ads} (kJ/mol)	-82.6	-131.5	-42.2
ΔG_{ads} (at 800 K, kJ/mol)	26.9	-12.5	65.3

Figure S24. The electronic (*ΔE*) and Gibbs free energies (*ΔG*) for adsorbing different CO molecules on the Co-Z5 structure; purple, pink, yellow, red, grey, white balls represent Co, Al, Si, O, C, H atoms, respectively.

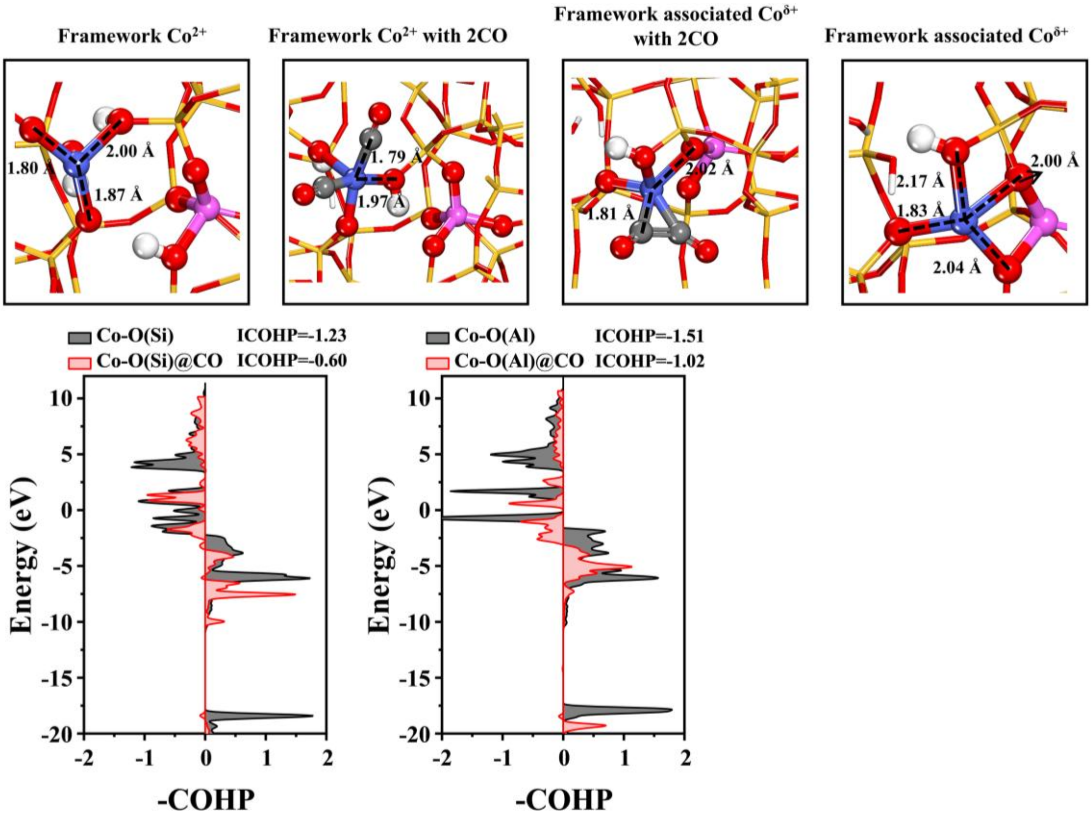

Figure S25. Structural modesl of 0.50Co-Z5(340) and (CO)0.50Co-Z5(340), and the COHP profiles of Co–O interactions (with comparable bond distance of 2.00/1.97/2.02 Å) between Co atoms and zeolite framework; purple, pink, yellow, red, grey, white balls represent Co, Al, Si, O, C, H atoms.

Note: For framework Co^{2+} sites, the CO adsorbates weakens Co–O interactions as reflected by that the ICOHP valuese decrease from -1.23 to -0.60. On framework-associated $Co^{\delta+}$ sites, the larger ICOHP values (-1.51 *vs.* -1.23, and -1.02 *vs.* -0.60 with CO adsorbates) suggest the AlO_4 can strengthen the Co–O interactions compared to that with SiO_4.

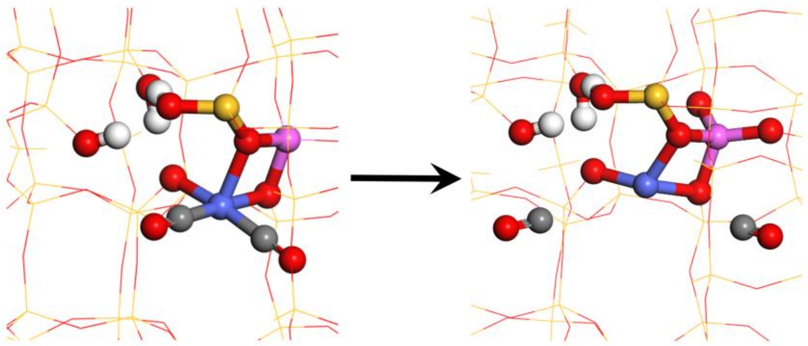

Structure	Adsorption → Desorption
ΔE_{des} (kJ/mol)	206.4
ΔG_{des} (at 800 K, kJ/mol)	13.9

Figure S26. CO desorption energy on (CO)0.50Co-Z5(340) model in which purple, pink, yellow, red, grey, white balls represent Co, Al, Si, O, C, H atoms;

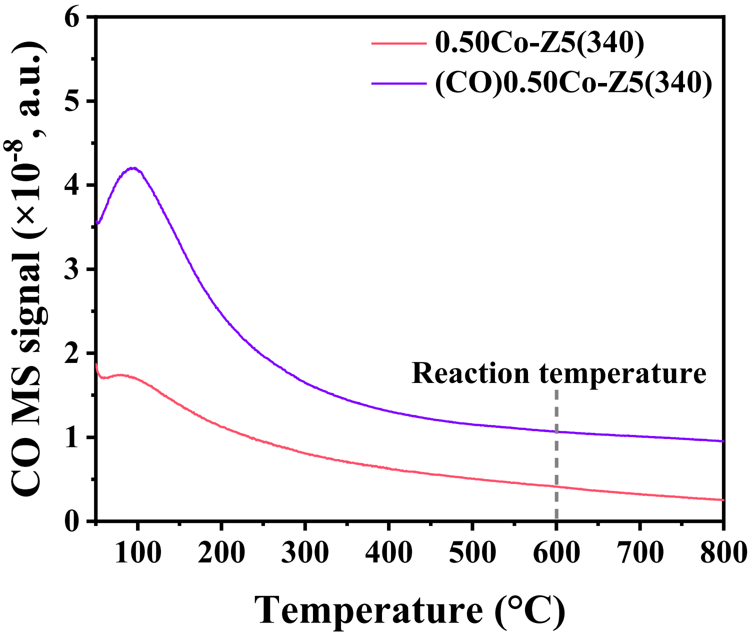

Figure S27. CO-TPD profiles for fresh and CO pre-treated 0.50Co-Z5(340).

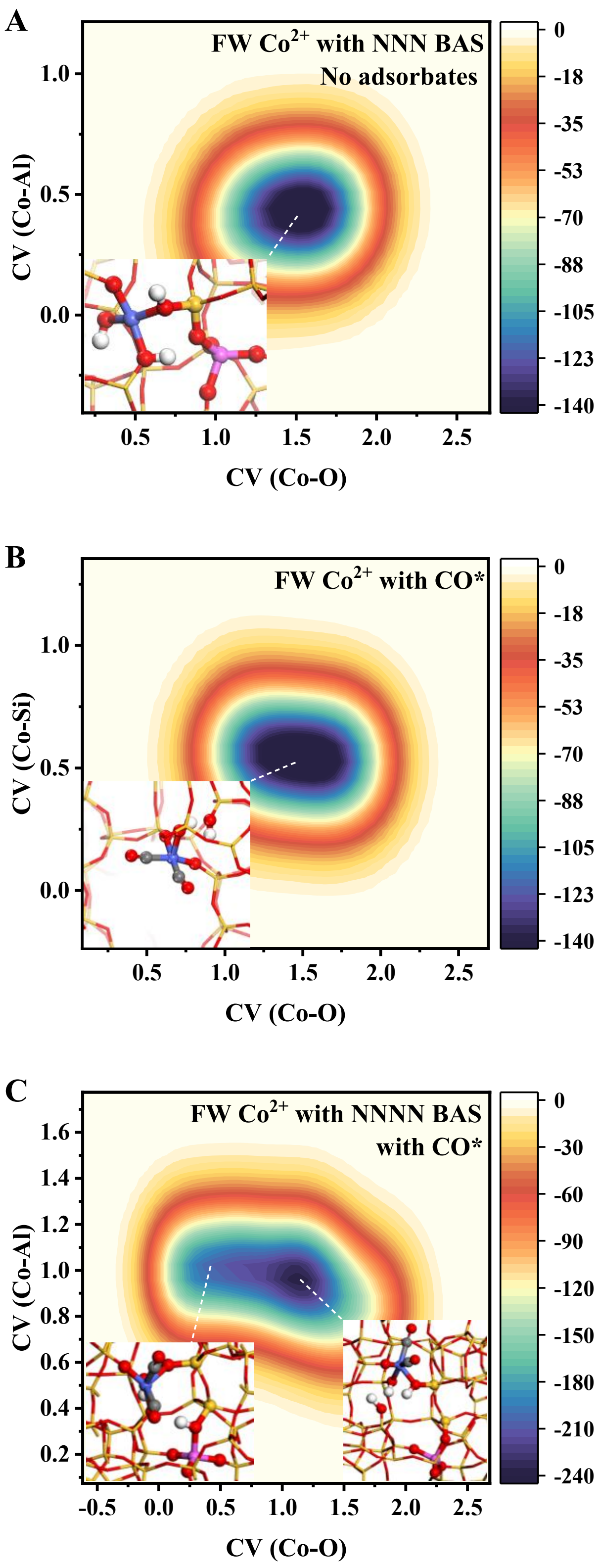

Figure S28. MTD simulations of dynamic cobalt transformation on (A) framework Co^{2+} with NNN-BAS but without CO* adsorbates; (B) framework Co^{2+} with adsorbed CO*; (C) framework Co^{2+} with NNNN BAS and CO* adsorbates; purple, pink, yellow, red, grey, white balls represent Co, Al, Si, O, C, H atoms.

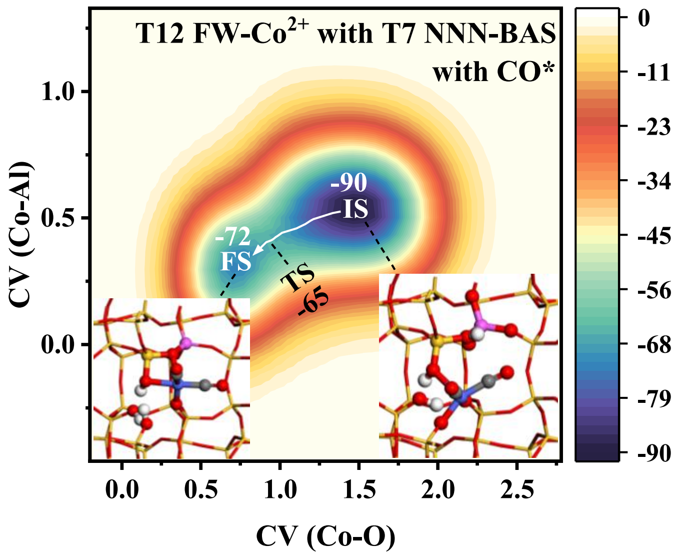

Figure S29. MTD simulations of alternatire Co-Z5 model comprising of T12 framework Co^{2+} and T7 NNN-BAS with CO adsorbates at 600°C; purple, pink, yellow, red, grey, white balls represent Co, Al, Si, O, C, H atoms.

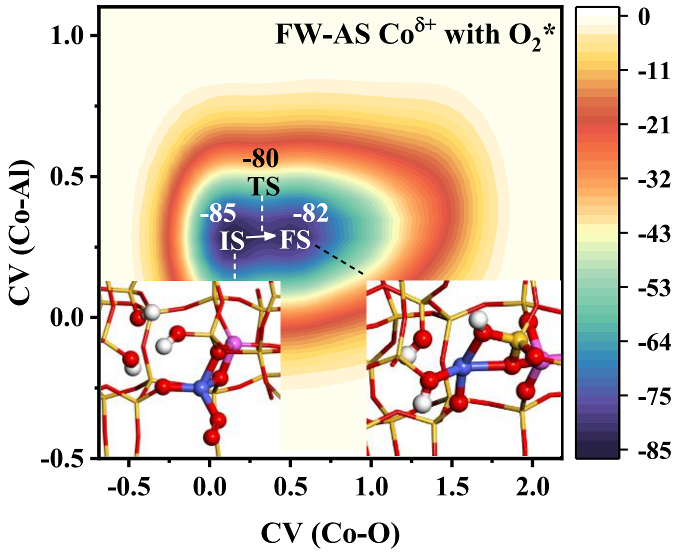

Figure S30. MTD simulations of (CO)0.50Co-Z5(340) with adsorbed O_2* at 25°C; purple, pink, yellow, red, grey, white balls represent Co, Al, Si, O, C, H atoms.

Note: The MTD results, upon O_2 interactions at 25°C, show increased CV(Co–O) values together with slight decrease in CV (Co–Al) values, which suggest the $Co^{\delta+}$ species would transform to the initial framework position upon air exposure.

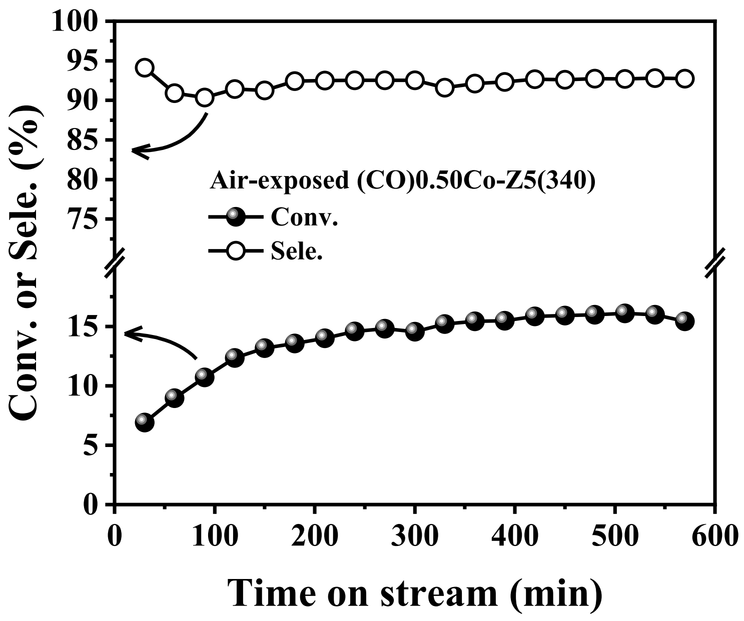

Figure S31. Ethane conversion and ethylene selectivty of 0.50Co-Z5(340) after CO pre-treatment and air exposure for 2 days.

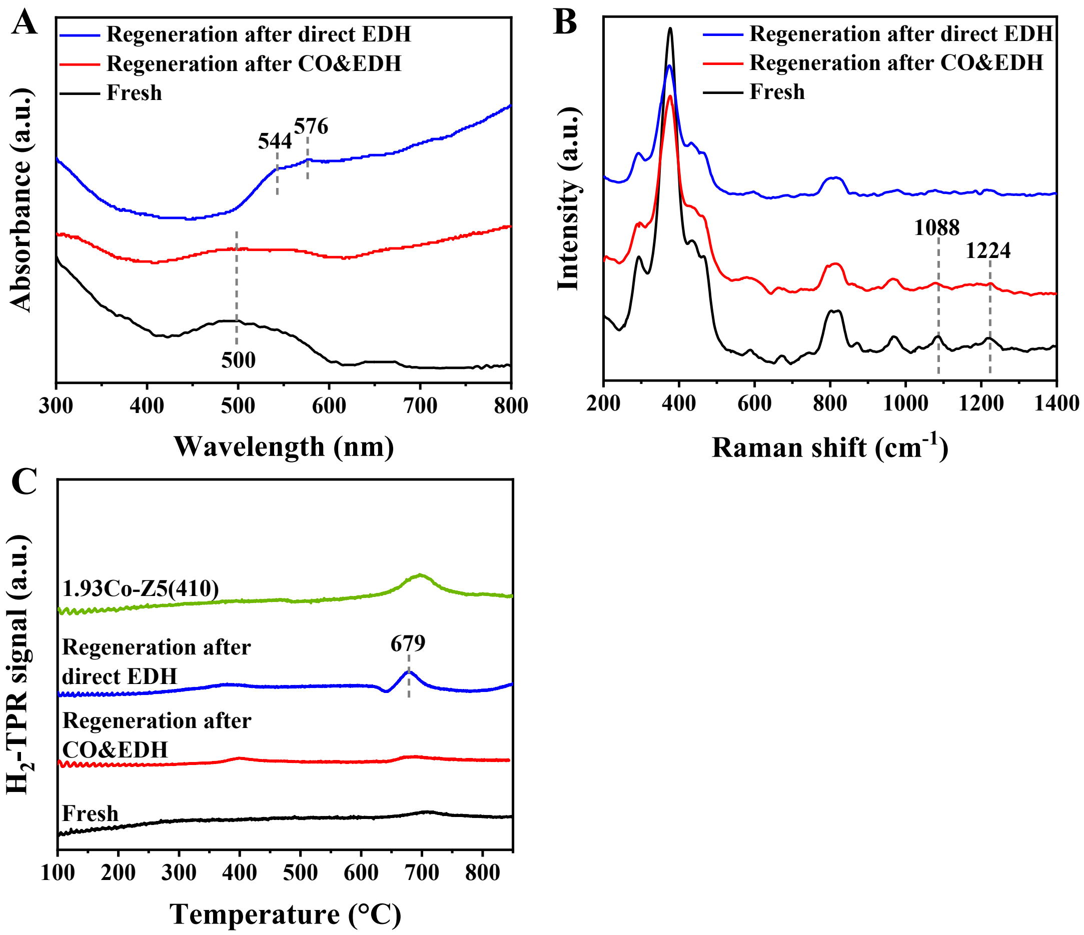

Figure S32. (A) UV-Vis DR; (B) Raman spectra; and (C) H_2-TPR profiles of 0.50Co-Z5(340) upon different treatment.

Note: The regenrated (CO)0.50Co-Z5(340) show comparable UV-Vis, Raman and H_2-TPR profiles compared to fresh sample. In contrast, the regnerated 0.50Co-Z5(340) without CO pre-treatment show UV-Vis signals at higher wavelength (544/576 nm) and H_2-TPR peaks around 679°C (similar as 4Co-Z5, due to encapsulated CoO clusters). And meantime, Raman singals due to Si-O-Co linkages (ref. [10]) disappear in regenrated 0.50Co-Z5(340). These results support the sintering of Co species for no activated 0.50Co-Z5(340), which are line line with *in-situ* XAS observations.

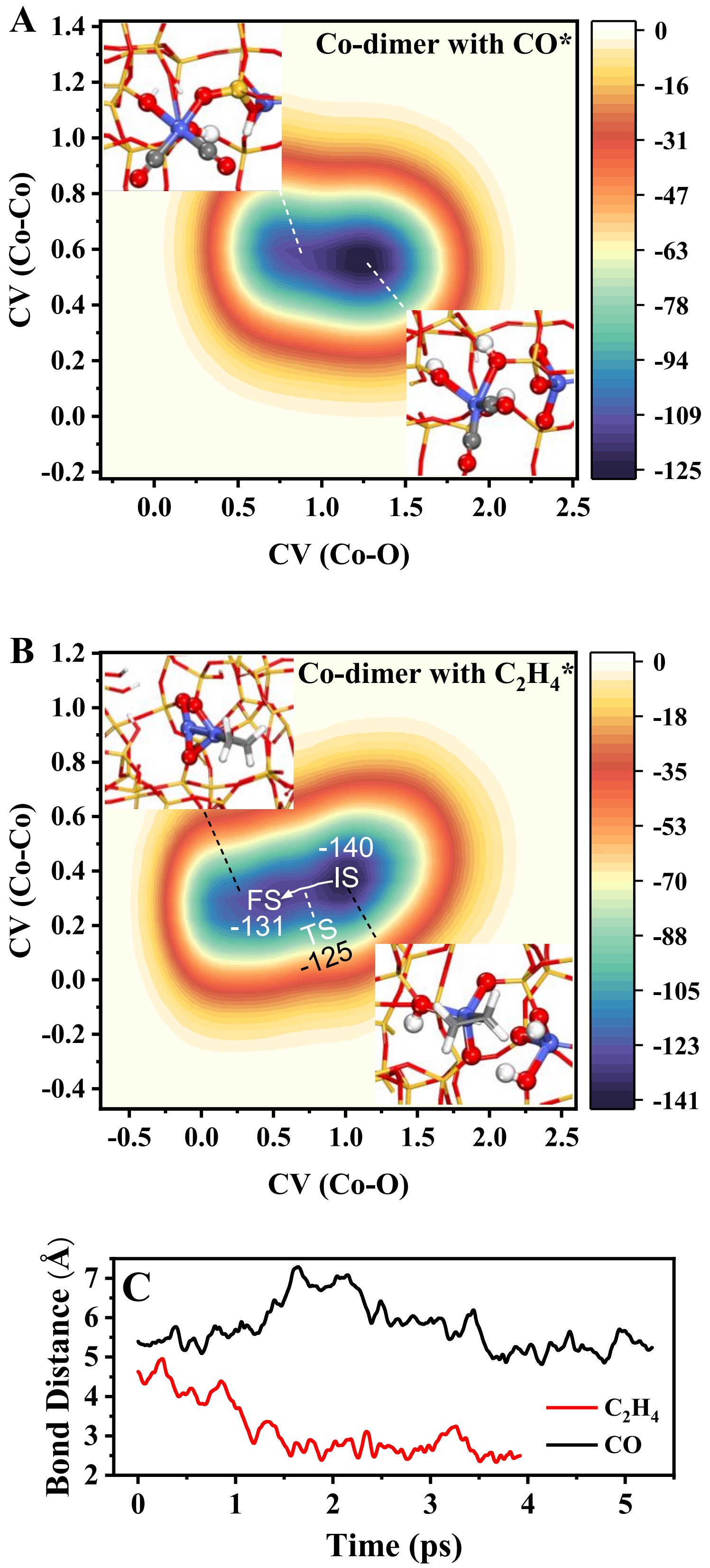

Figure S33. MTD simulations of (A) Co-dimer with adsorbed CO* at 600°C; (B) Co-dimer with adsorbed C_2H_4* at 600°C; (C) bond distance evolution between two cobalt

atoms in Co-dimer model with CO or C_2H_4* adsorbates, Insets show the typical structures; purple, pink, yellow, red, grey, white balls represent Co, Al, Si, O, C, H atoms, respectively.

Note: We constructed Co-dimer model which has the Co^{2+}–O–Si–O–Co^{2+} linkage within MFI framework. MTD simulations were used to explore the sintering of two next-nearest-neighbor cobalt atoms towards Co-dimer species. The results indicate that CO* adsorbates cannot induce the Co-dimer species, at least after applying 125 kJ/mol energy (Figure S33A). In comparison, cobalt sintering and the formation of Co-dimer can occur with C_2H_4* adsorbates with a ΔG barrier of ~29 kJ/mol (Figure S33B). The easier Co-dimer formation with C_2H_4* adsorbates was also indicated by the bond distance evolution during MTD simulations (Figure S33C). Therefore, the results indicate that C_2H_4* can promote the approaching of two cobalt species, which provide a rational and possible explanation for cobalt sintering in direct EDH sample, as reflected by *in-situ* XAS observations.

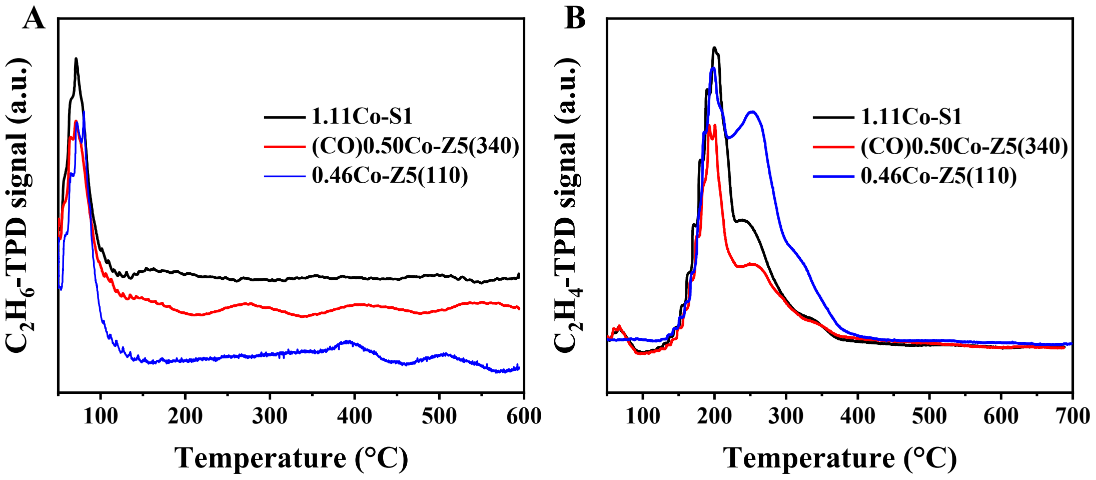

Figure S34. (A) C_2H_6 and (B) C_2H_4-TPD profiles on various catalysts.

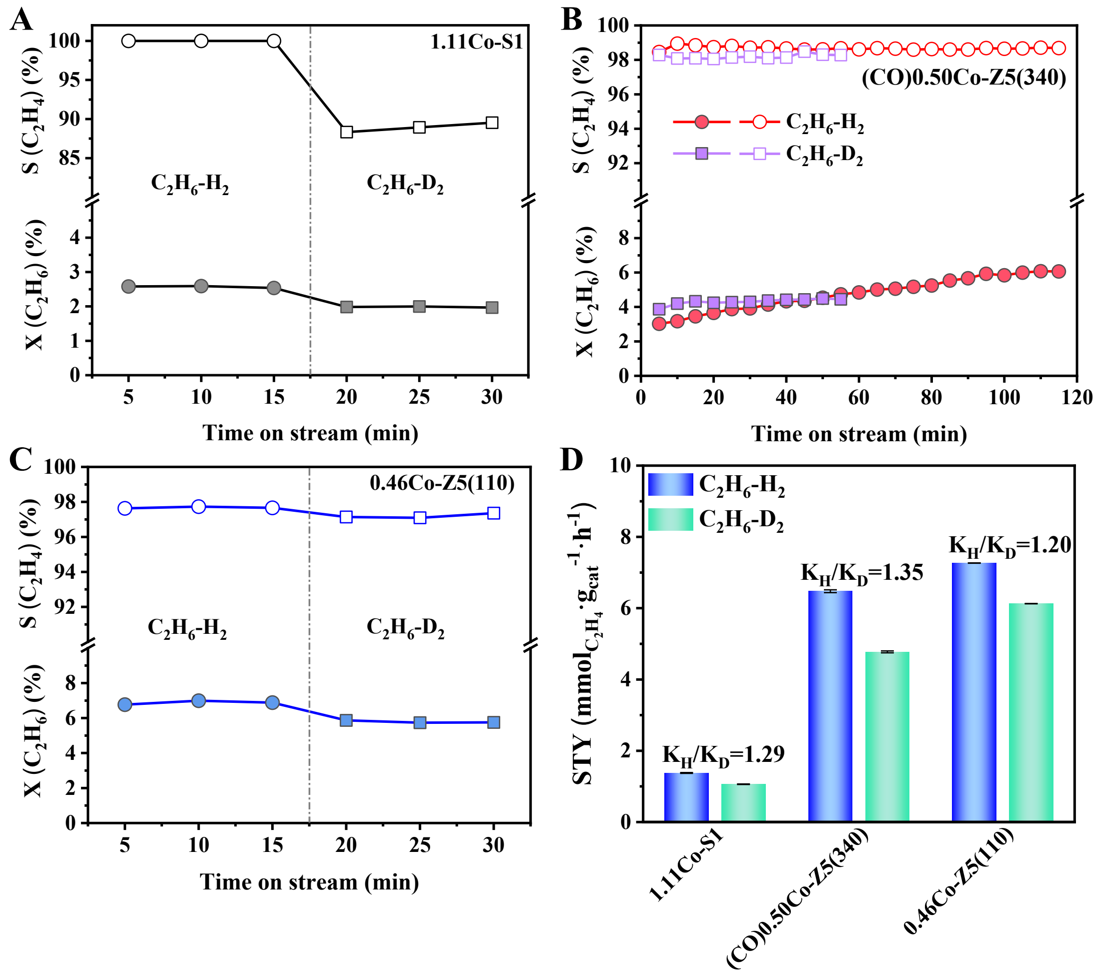

Figure S35. Kinetic studies under C_2H_6-H_2 or C_2H_6-D_2 feeds for various catalysts; reaction conditions: 0.1 g 1.11Co-S1 or 0.05 g for 0.50Co-Z5(340) and 0.46Co-Z5(110); 600°C; WHSV=2.4 h^{-1}; C_2H_6: H_2 or D_2: N_2=40 vol%: 20 vol%: 60 vol%.

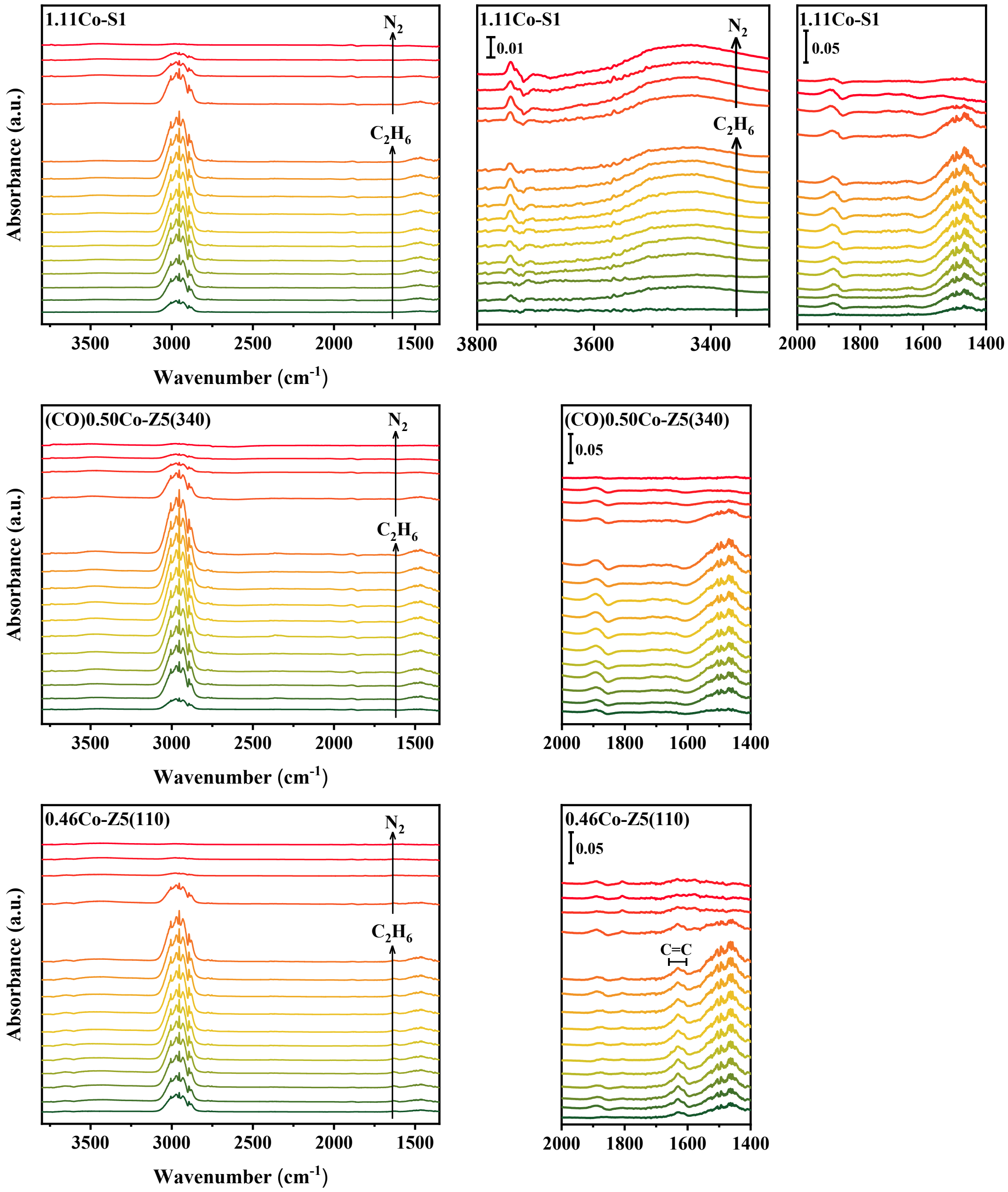

Figure S36. *In-situ* transmission FTIR spectra with C_2H_6 flow for various catalysts.

Note: Figure S36 and the results in Figure 4B,C confirm that 0.46Co-Z5(110) show gradually increased hydroxyl bands compared to 1.11Co-S1 and 0.50Co-Z5(340). In addition, the enlarged spectra within 2000-1400 cm^{-1} indicate obvious 1620 bands for 0.46Co-Z5(110), which can be assigned to C=C bonds in ethylene or aromatics.[15]

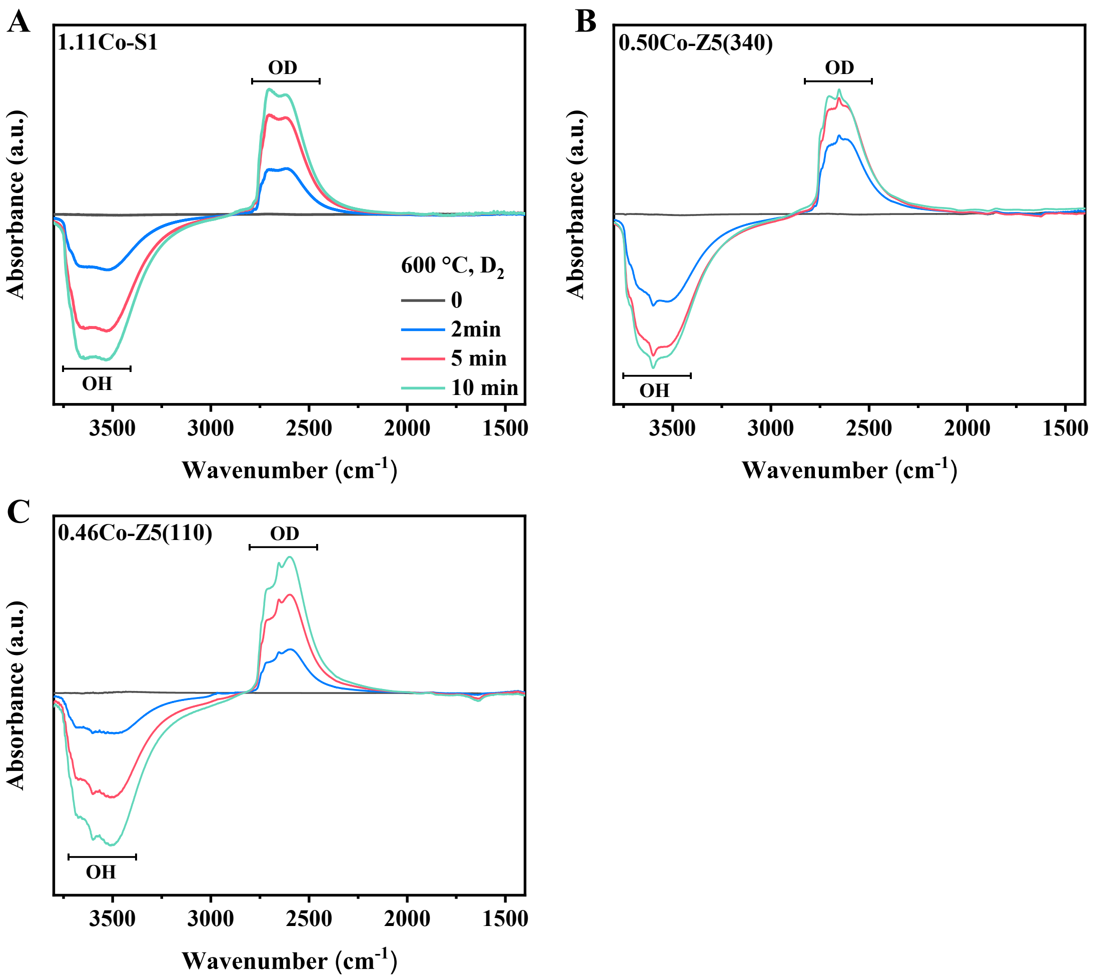

Figure S37. *In-situ* transmission FTIR spectra upon 10 vol% D_2 treatment at 600°C.

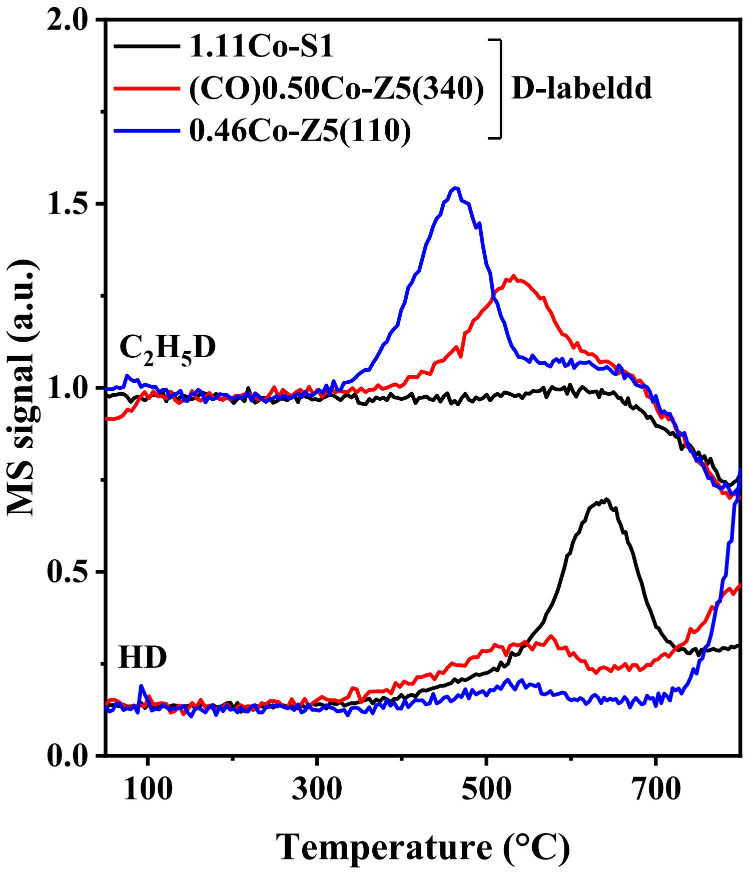

Figure S38. The comparison of C_2H_5D and HD evolution signals C_2H_6-TPSR profiles on different D-labeled catalysts.

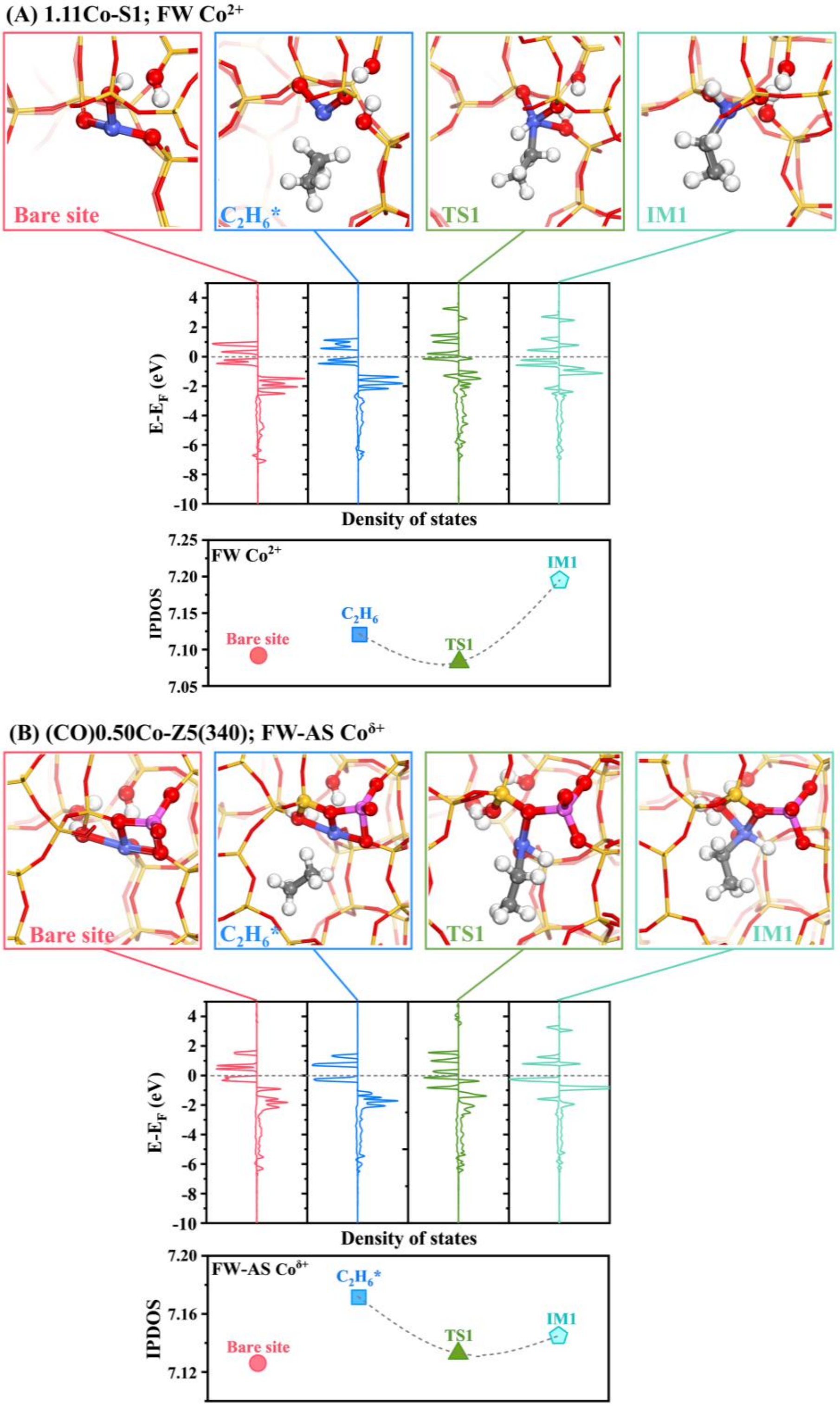

Figure S39. The structure models during ethane C–H bond cleavage and the corresponding variations in the integral density of states analysis for cobalt sites; purple, pink, yellow, red, grey, white balls represent Co, Al, Si, O, C, H atoms, respectively.

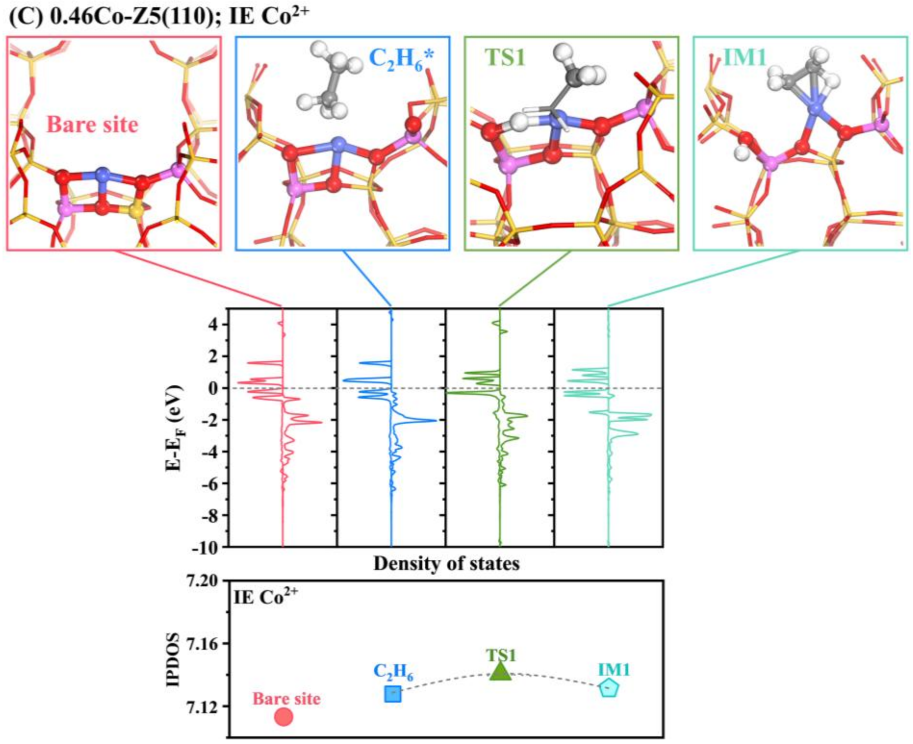

Continued Figure S39. The structure models during ethane C–H bond cleavage and the corresponding variations in the integral density of states analysis for cobalt sites; purple, pink, yellow, red, grey, white balls of models represent Co, Al, Si, O, C, H atoms, respectively.

Note: The integral density of states analysis can reflect the electron density on cobalt sites. In this sense, the results also suggest the electron depletion on Co sites during ethane activation for framework (FW) Co^{2+} and framework-associated (FW-AS) $Co^{\delta+}$ sites, while there is opposite trend on exchange (IE) Co^{2+} sites. The results are consistent with the findings from Bader charge analysis, which suggest the distinct electron transfer behavior for various Co sites catalyzed ethane activation.

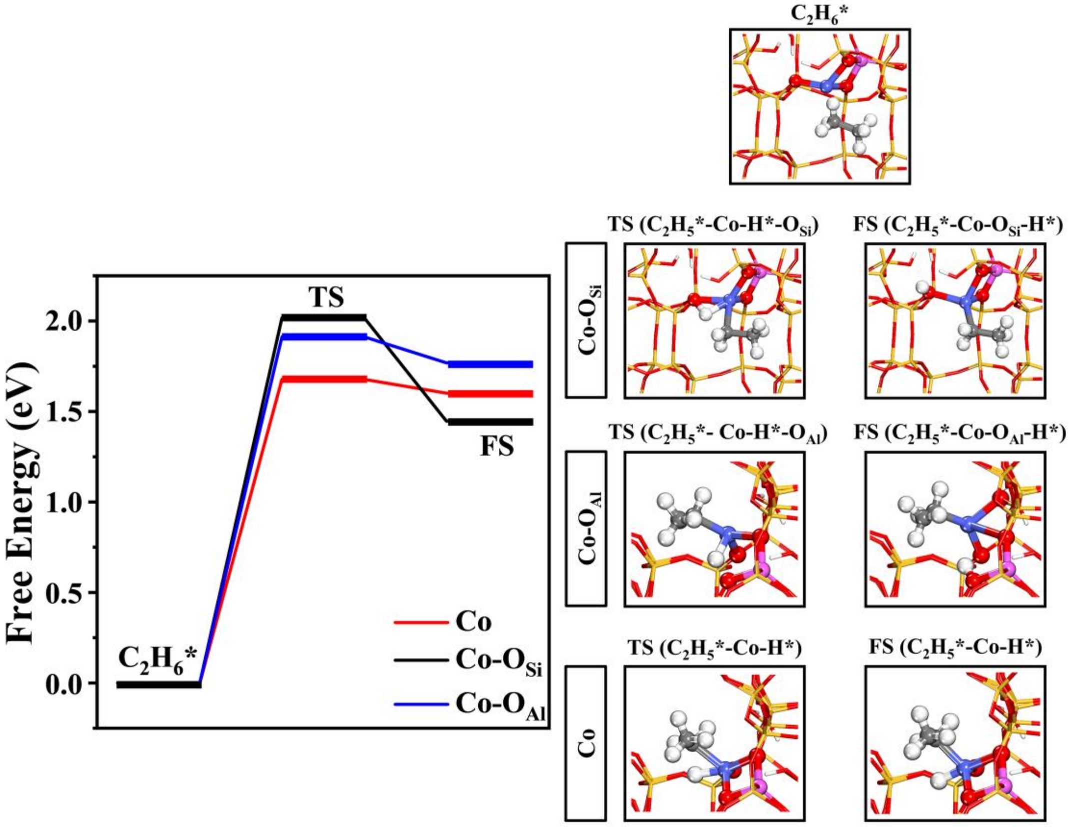

Figure S40. Reaction energy profiles and corresponding structures on $Co^{\delta+}$ sites with different reaction centers, TS state was searched by CI-NEB method, Co–O_{Si} and Co–O_{Al} indicate the Co–O sites in Co–O–Si and Co–O–Al linkages, purple, pink, yellow, red, grey, white balls represent Co, Al, Si, O, C, H atoms, respectively.

Note: We performed three independently static transition state search using CI-NEB method, with pre-setting different reaction centers. The results demonstrated that the ethane C–H bond cleavage on Co site requires a lower energy barrier compared to Co–O pairs. Therefore, the static transition state search also supports the oxidative addition mechanism is energetically for C–H bond activation on $Co^{\delta+}$ sites, with forming a three-center C_2H_5*–Co–H* transition state. The results indicate that MTD simulations do efficiently screen the energetically favorable reaction centers and catalytic mechanisms.

The TS and FS states on Co-O_{Si} center was used for further Bader charge analysis, which investigate the charge evoltion of cobalt sites during σ-bond metathesis C–H bond activation on $Co^{\delta+}$ sites, for comparative studies with oxidative mechanism searched via MTD simulations.

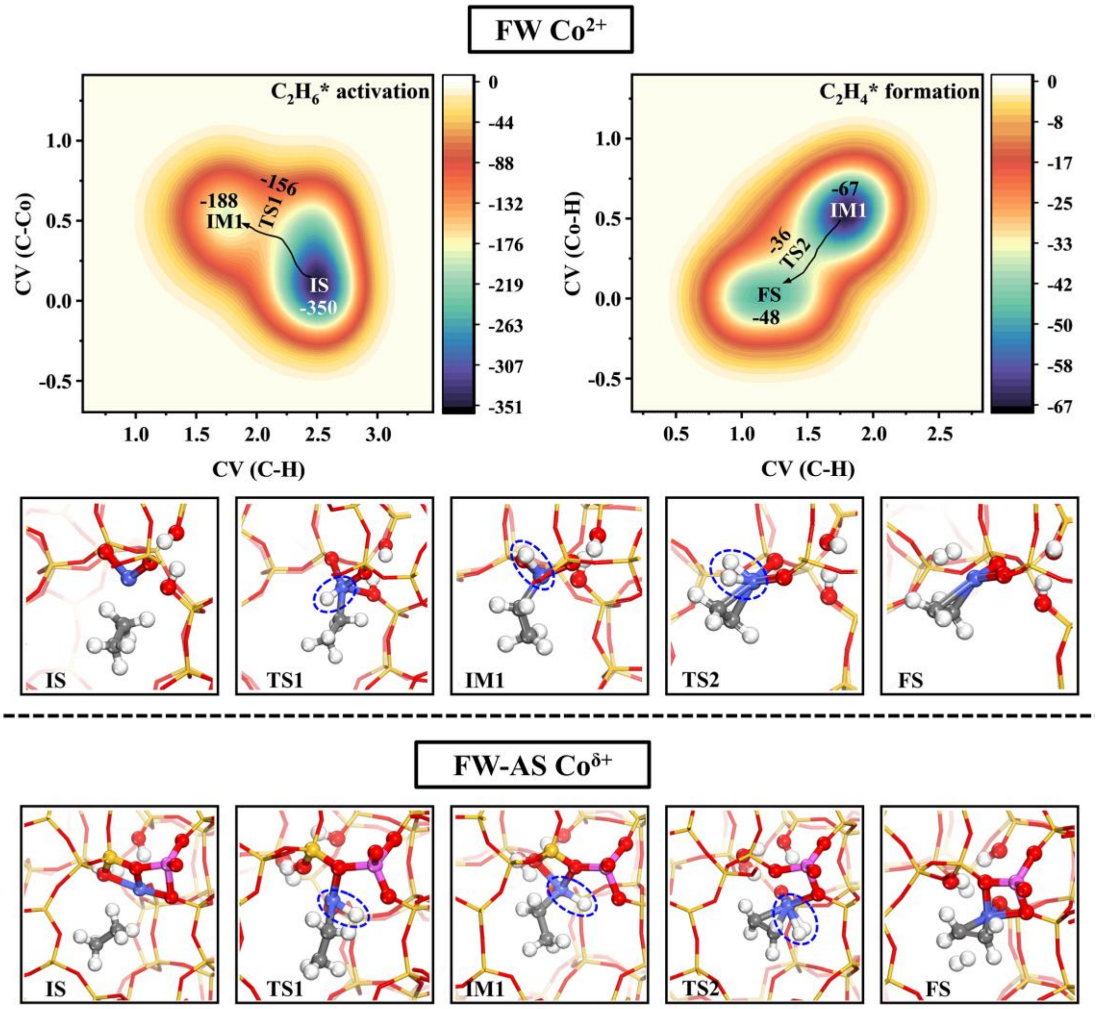

Figure S41. Free energy surface of MTD simulations of EDH reaction and the structural models of key intermediates on the 1.11Co-S1 and (CO)0.50Co-Z5(340) models; purple, pink, yellow, red, grey, white balls represent Co, Al, Si, O, C, H atoms, respectively.

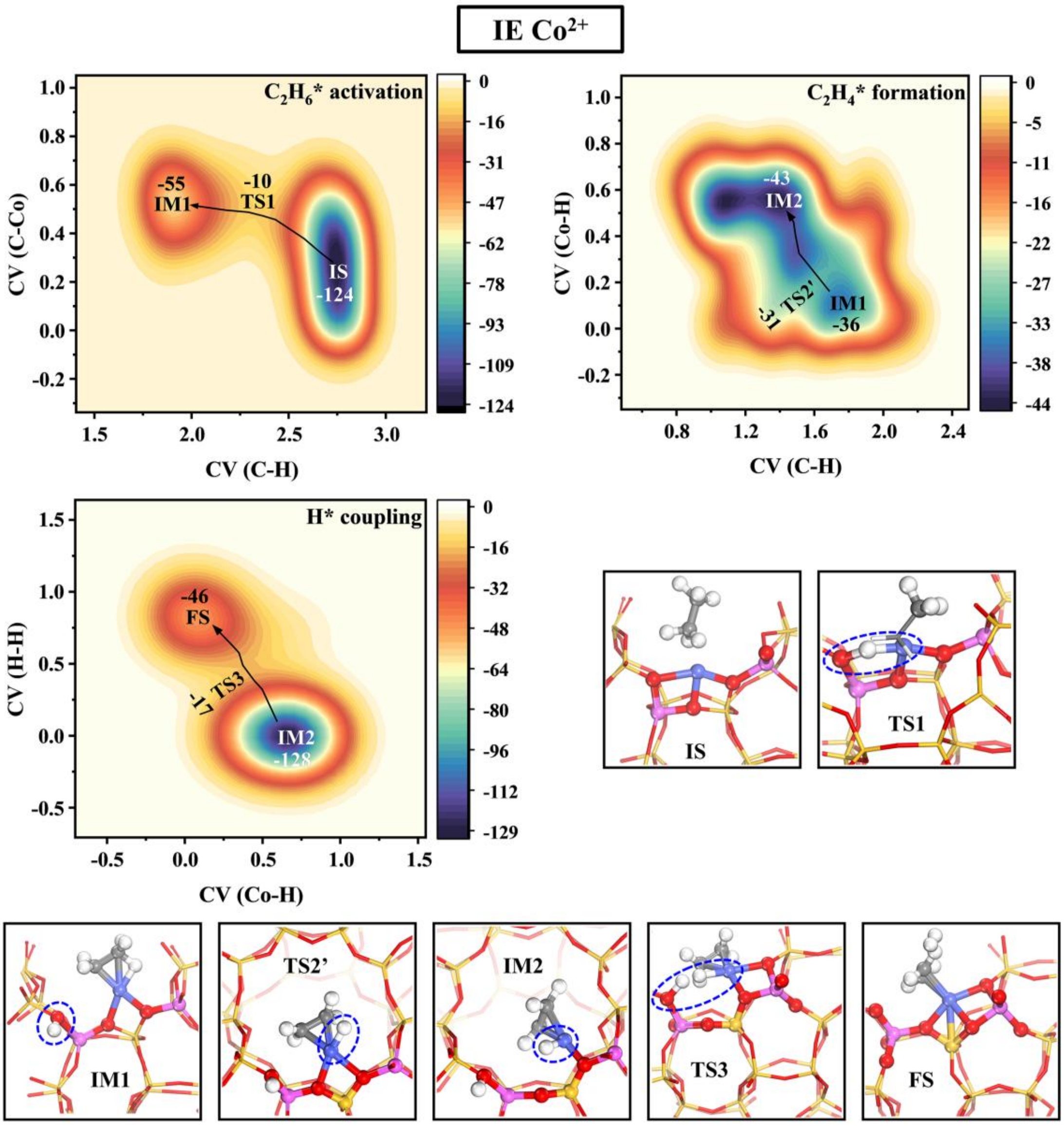

Continued Figure S41. Free energy surface of MTD simulations of EDH reaction and the structural models of key intermediates on the 0.46Co-Z5(110) model; purple, pink, yellow, red, grey, white balls represent Co, Al, Si, O, C, H atoms, respectively.

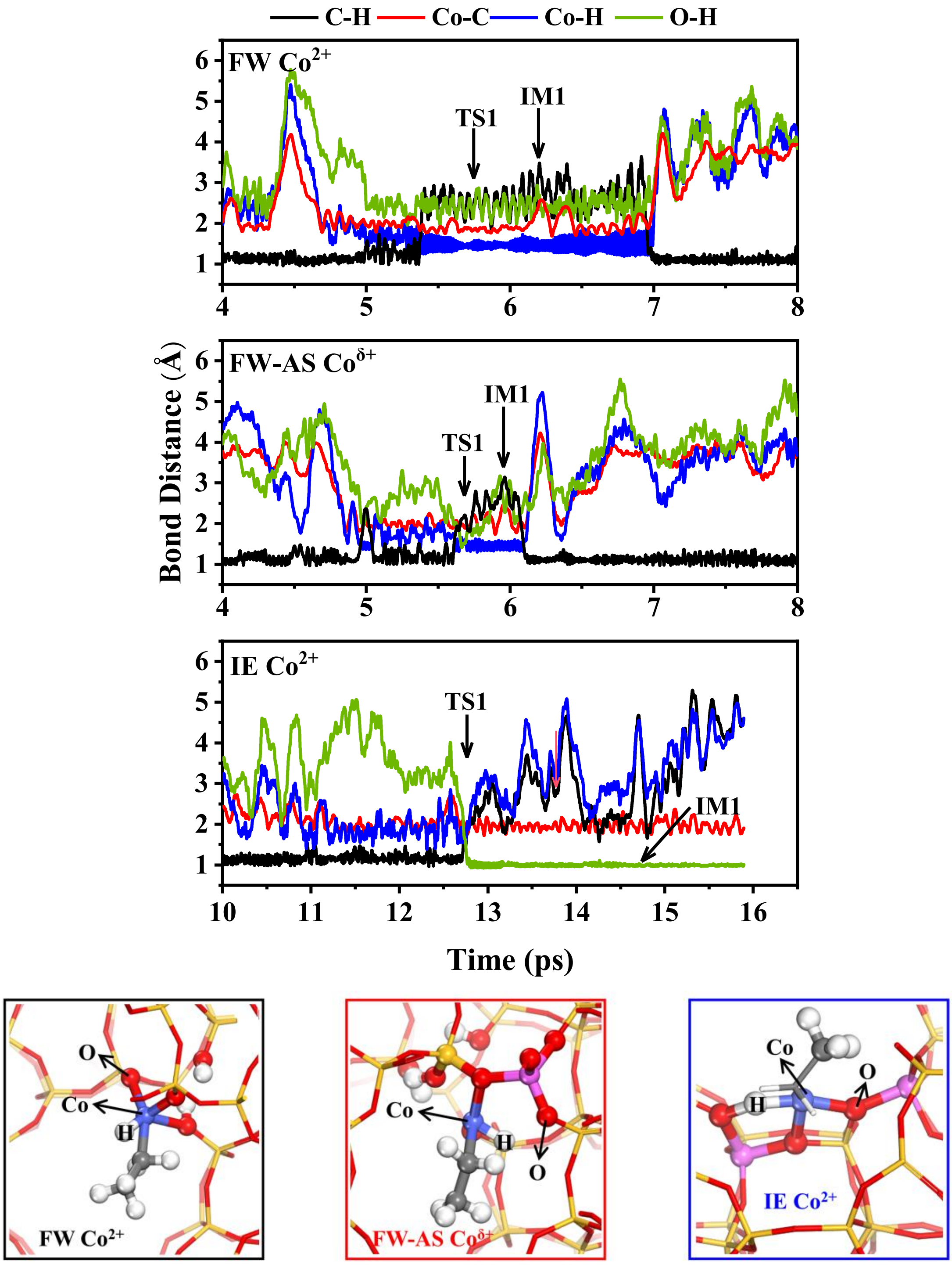

Figure S42. Variations in the distance of C–H, Co–H and O–H bonds and the corresponding strutctures to present the atom indentifier.

Note: Based on bond distance evolution from MTD trajectories, C–H bond clevage produces direct O–H bonds (bond distance of ~1.0 Å) on exchange Co^{2+} sites (O–H bond distance in zeolite hydroxyl is about 0.96 Å)[16], which belong to four center [C_2H_5*–Co–H–O*] transition state. In contrast, the framework Co^{2+} and framework-

associated $Co^{\delta+}$ sites show longer O–H bond distance than 1.5 Å at TS1/IM1 states. And the below structural models indicate the detached H* species are mainly stabilized by Co sites. Therefore, TS1 states belong to three center [C_2H_5*–Co–O*] coordination on framework Co^{2+} and framework-associated $Co^{\delta+}$ sites.

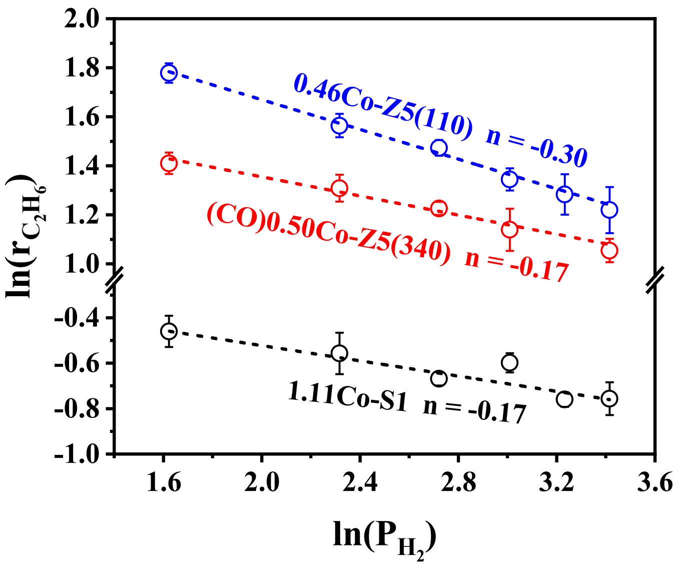

Figure S43. Reaction order of hydrogen on ethane conversion.

Note: Firstly, the elementary steps for EDH reaction are:

$C_2H_6(g) + * \overset{k1}{\leftrightarrow} C_2H_6*$ Step 1: C_2H_6 adsorption

$C_2H_6* + * \overset{k2}{\leftrightarrow} C_2H_5* + H*$ Step 2: C_2H_6* activation

$C_2H_5* + * \overset{k3}{\leftrightarrow} C_2H_4* + H*$ Step 3: C_2H_5* activation

$C_2H_4* \overset{k4}{\leftrightarrow} C_2H_4(g) + *$ Step 4: C_2H_4* desorption

$2H* \overset{k5}{\leftrightarrow} H_2(g) + 2*$ Step 5: H* removal

The Step 1, that is C_2H_6 adsorption, is rate-determining-step considering this step is most energy demanding in MTD simulations (Figure 5B), as well as the H-D kinetic isotope effects (k_H/k_D) are close to 1 (Figure S35). Therefore, the ethane conversion rate (r) can be calculated as

$$r = k1 \cdot k2 \cdot P_{C_2H_6} \cdot \theta_*^{\ 2}$$

The reaction equilibrium for all elementary steps can be described as:

$$k1 \cdot P_{C_2H_6} \cdot \theta^* = \theta_{C_2H_6}{}^*$$

$$k3 \cdot \theta_{C_2H_5}{}^* \cdot \theta^* = \theta_{C_2H_4}{}^* \cdot \theta_H{}^*$$

$$k4 \cdot \theta_{C_2H_4}{}^* = P_{C_2H_4} \cdot \theta^*$$

$$k5 \cdot (\theta_H{}^*)^2 = P_{H_2} \cdot (\theta^*)^2$$

The site coverage of surface species is:

$$\theta_{C_2H_6}{}^* = k1 \cdot P_{C_2H_6} \cdot \theta^*$$

$$\theta_{C_2H_4}{}^* = k4^{-1} \cdot P_{C_2H_4} \cdot \theta^*$$

$$\theta_H{}^* = k5^{-0.5} \cdot (P_{H_2})^{0.5} \cdot \theta^*$$

$$\theta_{C_2H_5}{}^* = k3^{-1} \cdot \theta_{C_2H_4}{}^* \cdot \theta_H{}^* \cdot \theta^{*-1} = k3^{-1} \cdot k4^{-1} \cdot k5^{-0.5} \cdot$$

$$P_{C_2H_4} \cdot \theta^* \cdot (P_{H_2})^{0.5}$$

The site balance of surface species is:

$$\theta^* = \frac{1}{1+k1 \cdot P_{C_2H_6}+k4^{-1} \cdot P_{C_2H_4}+k5^{-0.5} \cdot (P_{H_2})^{0.5}+k3^{-1} \cdot k4^{-1} \cdot k5^{-0.5} \cdot P_{C_2H_4} \cdot (P_{H_2})^{0.5}}$$

Therefore, the reaction rate *r* can be calculated as:

$$r = \frac{k1 \cdot k2 \cdot P_{C_2H_6}}{(1+k1 \cdot P_{C_2H_6}+k4^{-1} \cdot P_{C_2H_4}+k5^{-0.5} \cdot (P_{H_2})^{0.5}+k3^{-1} \cdot k4^{-1} \cdot k5^{-0.5} \cdot P_{C_2H_4} \cdot (P_{H_2})^{0.5})^2}$$

Considering the low ethane conversion for measuring hydrogen reaction order, the $\theta_{C_2H_5}{}^*$ and $\theta_{C_2H_4}{}^*$ can be ignored. Assuming the hydrogen is the most abundant intermediates, the reaction rate r can be simplified as:

$$r = \frac{k1 \cdot k2 \cdot P_{C_2H_6}}{(1+k5^{-0.5} \cdot (P_{H_2})^{0.5})^2}$$

So, we can obtain:

$$\ln (r) = \ln (k1) + \ln (k2) + \ln (P_{C_2H_6}) - 2\ln (1+k5^{-0.5} \cdot (P_{H_2})^{0.5})$$

The hydrogen reaction order, n (H_2), with respect to ethane conversion is:

$$n (H_2) = \partial \ln(r) \;/\; \partial \, ln_{P_{H_2}} = - \frac{k5^{-0.5} \cdot (P_{H_2})^{0.5}}{1+k5^{-0.5} \cdot (P_{H_2})^{0.5}} = -\theta_H{}^*$$

Accordingly, the more sluggish H_2* formation kinetic corresponds more negative hydrogen reaction order, which is consistent with our case for 0.46Co-Z5(110) catalyst.

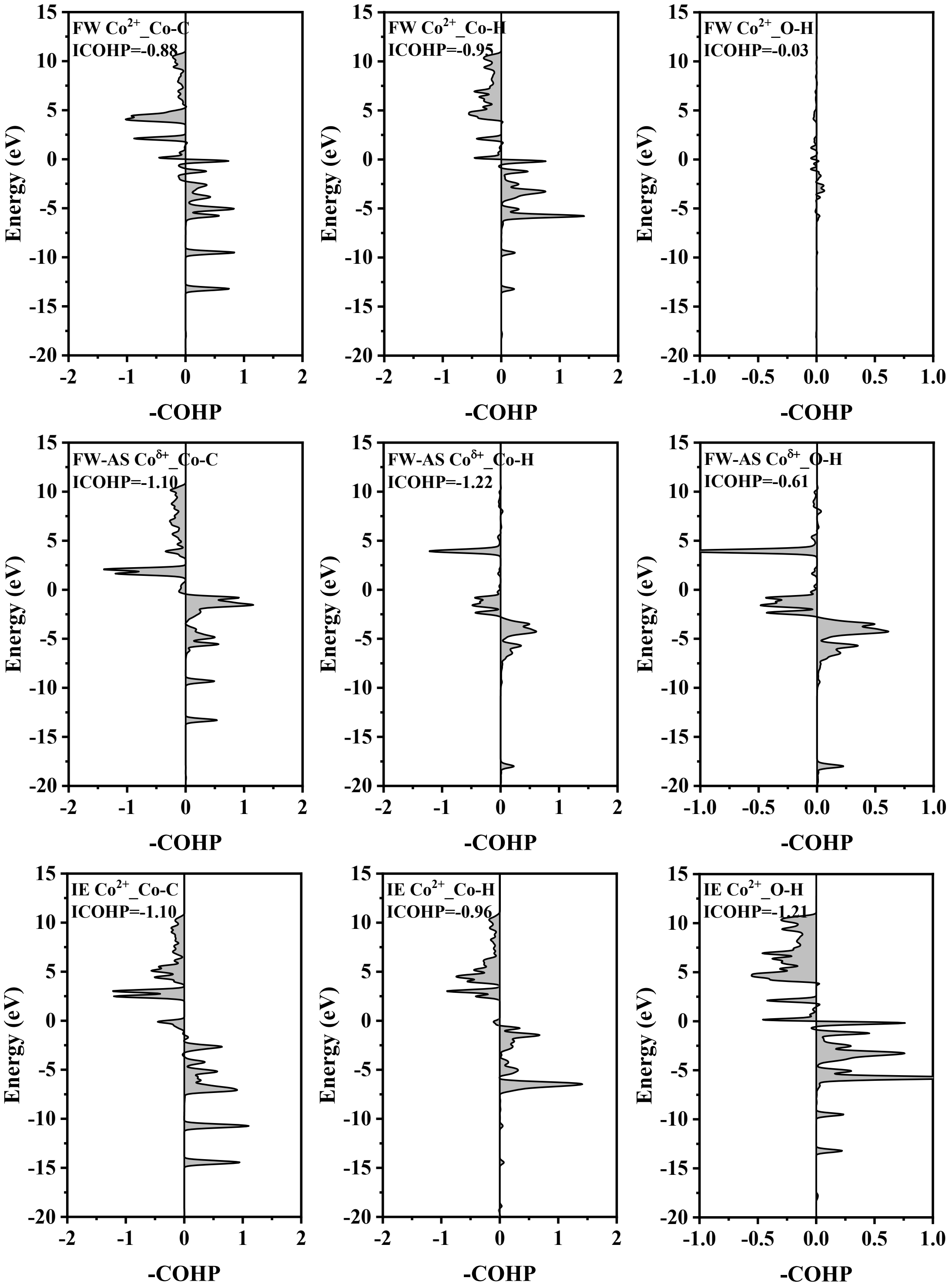

Figure S44. COHP profiles of Co–C, Co–H and O–H interactions between Co/O atoms and C_2H_5*/H* intermediates.

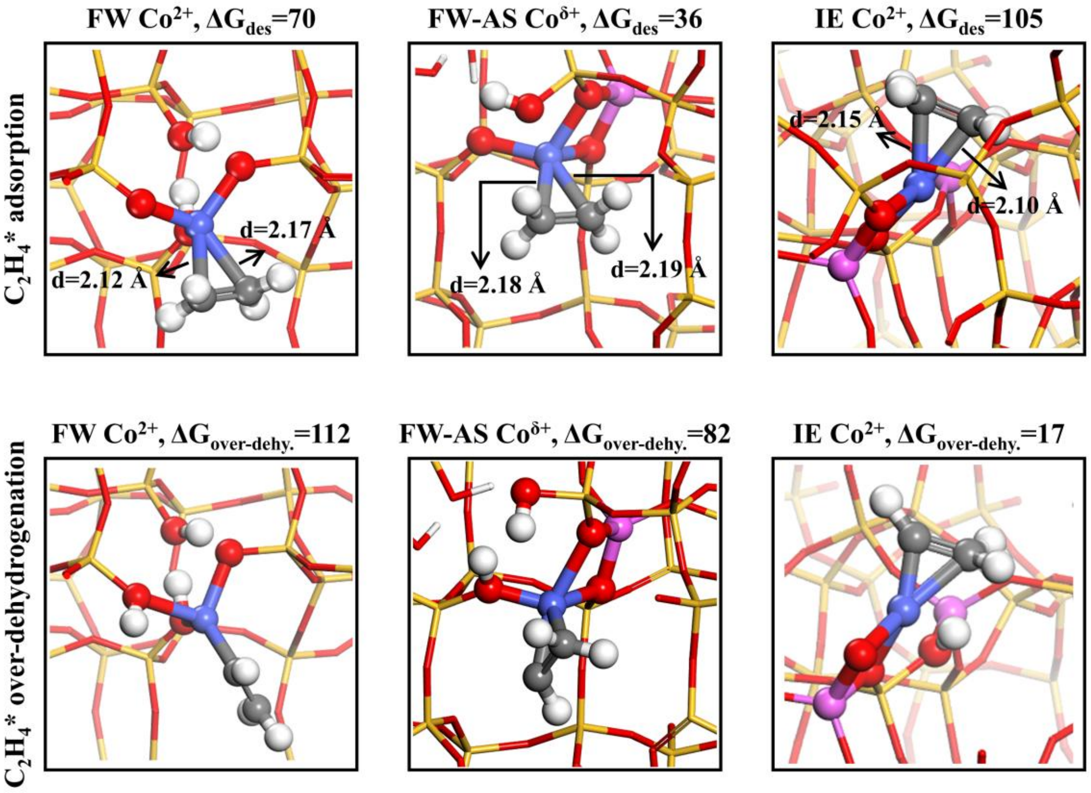

Figure S45. Gibbs free energy for C_2H_4* desorption and over-dehydrogenation at various Co sites; units are kJ/mol; purple, pink, yellow, red, grey, white balls represent Co, Al, Si, O, C, H atoms, respectively.

Supporting Tables

Table S1. The texture property of various catalysts

Catalyst	S_{BET} (m^2/g)	V_{micro} (cm^3/g)	Co wt%		SiO_2/Al_2O_3		Co/Al [c]
			Theoretical[a]	Actual[b]	Theoretical[a]	Actual[b]	
1.11Co-S1	368.5	0.165	1.0	1.11	/	/	/
0.50Co-Z5(340)	372.5	0.163	1.0	0.50	550	340	0.86
0.46Co-Z5(110)	354.7	0.144	1.0	0.46	200	110	0.25
0.83Co-Z5(557)	/	/	1.0	0.83	800	557	2.35
0.55Co-Z5(200)			1.0	0.55	300	200	0.58
0.31Co-Z5(56)			1.0	0.31	100	56	0.08
0.38Co-Z5(330)			0.5	0.38	550	330	0.64
1.93Co-Z5(410)			2.0	1.93	550	410	4.02

Note: [a] Theoretical indicates the theoretical cobalt content and SiO_2/Al_2O_3 molar ratios in synthesis gel; [b] Actual indicates the actual values determined by ICP-OES; [c] Co/Al represents the actual Co/Al molar ratio from ICP-OES.

Table S2. EXAFS fitting parameters for various fresh catalysts; see fitting curves in Figure S4.

Catalysts	Shell	CN [a]	R/ Å [b]	$\Delta\sigma^2$ / Å^2 [c]	E_0 /eV [d]	Fitting Window
**1.11Co-S1**	Co–O	2.2	2.07	0.006	2.3	k: 2.59-10.95 Å^{-1}; R: 1.01-3.19 Å
	Co–Si	2.7	3.25	0.0008	-2.7	
**0.50Co-Z5(340)**	Co–O	2.3	2.07	0.005	2.1	k: 2.57-11.42 Å^{-1}; R: 0.94-3.10 Å
	Co–Si	2.8	3.24	0.0002	-2.8	
**0.46Co-Z5(110)**	Co–O	2.4	2.08	0.006	1.4	k: 2.59-10.98 Å^{-1}; R: 1.01-3.28 Å
	Co–Si	2.7	3.26	0.0003	3.1	

Note: [a] CN, coordination number; [b] R, bonding distance; [c] $\Delta\sigma^2$, Debye-Waller, [d] E_0, inner potential shift; average standard error for above parameters is R, 0.01 Å; CN, 9.2 %; $\Delta\sigma^2$, 10.5 %; E_o 0.4 eV.

Table S3. Activity comparison between our described (CO)0.50Co-Z5(340) catalyst and the previously reported non-Pt EDH materials.

Catalyst	M_{metal} (wt%)	T (°C)	Ethane WHSV(h^{-1})	Feed (vol%)	Conv. (%)	Sele. (%)	STY($mol_{C_2H_4}\cdot g_{metal}^{-1}\cdot h^{-1}$)	K_d (h^{-1}) [a]	Ref.
CO-0.50Co-Z5(340)	0.50	600	5.91	Pure C_2H_6	10.75	91.3	2.63	0.024	This work
Co/S1-aw	0.45	590	6.50	Pure C_2H_6	10.0	97.0	2.94	~0	10
Co/SAPO-34-IE	0.45	600	5.32	C_2H_6: N_2 = 90: 10	7.0	96.5	1.80	0.012	17
Co/HZSM-5-IE	1.42	600	5.23	C_2H_6: N_2 = 90: 10	21.5	40.1	0.73	0.067	18
Co@MFI	6.4	600	10.65	C_2H_6: N_2 = 90: 10	16.2	99.0	0.64	/	19
Co@MCM-41	3.1	600	5.32	C_2H_6: N_2 = 90: 10	12.5	99.0	0.48	/	20
CO-0.50Co-Z5(340)	0.50	600	4.73	C_2H_6: N_2 = 80: 20	14.5	92.6	2.87	0.036	This work
CO-0.50Co-Z5(340)	0.50	600	2.37	C_2H_6: N_2 = 40: 60	18.7	90.9	1.82	0.055	This work
Zn/Na-SSZ-13	1.03	650	3.55	C_2H_6: CO_2 = 50: 50	30	95	2.22	/	21
Co/S1	1.0	650	4.73	C_2H_6: CO_2=1: 1	17.5	95	1.78	/	22
FeS1-EDTA	0.8	600	1.18	C_2H_6: Ar = 30: 70	26.3	97.5	0.86	~0	23

CO-0.50Co-Z5(340)	0.50	600	2.37	C_2H_6: N_2 = 20: 80	21.6	95.1	2.20	0.048	This work
0.4Cr@MFI	0.4	650	2.4	C_2H_6: Ar = 20: 80	18	99	2.42	~0	24
Ga-CHA	3.2	660	1.18	C_2H_6: He = 10: 90	57.4	76.1	0.36	~0	25
In-CHA	3.2	660	1.18	C_2H_6: He = 10: 90	25.9	96.1	0.21	~0	25
CO-0.50Co-Z5(340)	0.50	600	0.59	C_2H_6: N_2 = 5: 95	46.0	98.7	1.21	0.009	This work
Co_2P-E/SBA-15	5.3	600	370	C_2H_6: H_2: N_2 = 5: 5 90	1.1	90	1.56	/	26
Co/SiO_2	1.18	625	1.77	C_2H_6: N_2 = 4: 96	35.0	97.0	1.15	0.209	11
Co/DeAl-MOR	3.0	650	0.52	C_2H_6: N_2 = 3: 97	53.4	92.1	0.19	0.211	27
Co/HZSM-5	3.0	650	0.53	C_2H_6: N_2 = 3: 97	50.6	88.6	0.18	0.190	28
Co/HZSM-5	0.44	600	0.29	C_2H_6: N_2 = 5: 95	18.2	89.3	0.13	0.024	29
CO-0.50Co-Z5(340)	0.50	550	4.73	C_2H_6: N_2 = 80: 20	8.8	96.3	1.81	0.007	This work
Zn@ZSM-5	2.79	550	3.55	C_2H_6: N_2 = 90: 10	14.3	42.8	0.18	/	30

Note: [a] K_d represents the deactivation rate constant during EDH reaction, which was calculated from the equation[31]: $K_d = (\ln\left[\frac{(1-Conv._{final})}{Conv._{final}}\right] - \ln\left[\frac{(1-Conv._{initial})}{Conv._{initial}}\right])/t$; where $Conv._{final}$ and $Conv._{initial}$ represent the ethane conversions at final and initial reaction, and t is the reaction time.

Table S4. The collective variables applied in various MTD simulations.

Description	Collective variables
Cobalt evolution with CO* adsorbates in 0.50Co-Z5(340)	CV(Co–O): average coordination number of cobalt and four nearby oxygen atoms<br>CV(Co–Al): distance between Co and Al atoms
Cobalt evolution without CO* adsorbates in 0.50Co-Z5(340)	CV(Co–O): average coordination number of cobalt and four nearby oxygen atoms<br>CV(Co–Al): distance between Co and Al atoms
RWGS reaction between Si-OH-Co and CO molecules	CV(Co–O): average coordination number of cobalt and four nearby oxygen atoms<br>CV(C–O): average coordination number of carbon and four oxygen atoms (nearby Co)
Cobalt evolution with CO* adsorbates in 1.11Co-S1	CV(Co–O): average coordination number of cobalt and four nearby oxygen atoms<br>CV(Co–Si): distance between Co and nearby Si atoms
Cobalt evolution with O_2* adsorbates in (CO)0.50Co-Z5(340)	CV(Co–O): average coordination number of cobalt and four nearby oxygen atoms<br>CV(Co–Al): distance between Co and Al atoms
Cobalt evolution with CO* adsorbates in Co-dimer	CV(Co–O): average coordination number of cobalt and four nearby oxygen atoms<br>CV(Co–Co): distance between Co and Co atoms
Cobalt evolution with C_2H_4* adsorbates in Co-dimer	CV(Co–O): average coordination number of cobalt and four nearby oxygen atoms<br>CV(Co–Co): distance between Co and Co atoms
C_2H_6 activation on 1.11Co-S1	CV(C–H): average coordination number of carbon and three H atoms in CH_3 moiety (of C_2H_6*)<br>CV(C–Co): coordination number of Co and C atoms (from ethane)

C_2H_4 formation on 1.11Co-S1	CV(C–H): average coordination number of carbon and three H atoms in CH_3 moiety (of C_2H_5*)<br>CV(Co–H): coordination number of cobalt and hydrogen (from ethane activation) atoms
C_2H_6 activation on (CO)0.50Co-Z5(340)	CV(C–H): average coordination number of carbon and three H atoms in CH_3 moiety (of C_2H_6*)<br>CV(C–Co): coordination number of Co and C atoms (from ethane)
C_2H_4 formation on (CO)0.50Co-Z5(340)	CV(C–H): average coordination number of carbon and three H atoms in CH_3 moiety (of C_2H_5*)<br>CV(Co–H): coordination number of cobalt and hydrogen (from ethane activation) atoms
C_2H_6 activation on 0.46Co-Z5(110)	CV(C–H): average coordination number of carbon and three H atoms in CH_3 moiety (of C_2H_6*)<br>CV(C–Co): coordination number of Co and C atoms (from ethane)
C_2H_4 formation on 0.46Co-Z5(110)	CV(C–H): average coordination number of carbon and three H atoms in CH_3 moiety (of C_2H_5*)<br>CV(Co–H): coordination number of cobalt and hydrogen (from ethane activation) atoms
H coupling on 0.46Co-Z5(110)	CV(H–H): coordination number of two detached H atoms (from ethane C–H bond cleavage)<br>CV(Co–H): coordination number of Co and H atoms

Suppporting References

(1) Ravel, B.; Newville, M. ATHENA, ARTEMIS, HEPHAESTUS: data analysis for X-ray absorption spectroscopy using IFEFFIT. *Synchrotron Radiation* **2005**, *12*, 537-541.

(2) Lee, P. A.; Citrin, P.; Eisenberger, P.; Kincaid, B. Extended X-ray absorption fine structure-its strengths and limitations as a structural tool. *Rev. Mod. Phys.* **1981**, *53*, 769-806.

(3) Chantler, C.; Boscherini, F.; Bunker, B. International tables for crystallography volume I: X-ray absorption spectroscopy and related techniques. **2024**, https://doi.org/10.1107/97809553602060000116.

(4) Rehr, J.; Albers, R. Theoretical approaches to X-ray absorption fine structure. *Rev. Mod. Phys.* **2000**, *72*, 621-654.

(5) Henkelman, G.; Jónsson, H. Improved tangent estimate in the nudged elastic band method for finding minimum energy paths and saddle points. *J. Chem. Phys.* **2000**, *113*, 9978-9985.

(6) Heyden, A.; Bell, A.; Keil, F. Efficient methods for finding transition states in chemical reactions: Comparison of improved dimer method and partitioned rational function optimization method. *J. Chem. Phys.* **2005**, *123*, 224101.

(7) Nelson, R.; Ertural, C.; George, J.; Deringer, V.; Hautier, G.; Dronskowski, R. LOBSTER: Local orbital projections, atomic charges, and chemical-bonding analysis from projector-augmented-wave-based density-functional theory. *J. Comput. Chem.* **2020**, *41*, 1931-1940.

(8) Hu, Z.; Qin, G.; Han, J.; Zhang, W.; Wang, N.; Zheng, Y.; Jiang, Q.; Ji, T.; Yuan, Z.; Xiao, J.; Wei, Y.; Liu, Z. Atomic insight into the local structure and microenvironment of isolated Co-motifs in MFI zeolite frameworks for propane dehydrogenation. *J. Am. Chem. Soc.* **2022**, *144*, 12127-12137.

(9) Estes, D.; Siddiqi, G.; Allouche, F.; Kovtunov, K.; Safonova, O.; Trigub, A.; Koptyug, I.; Copéret, C. C–H activation on Co, O sites: Isolated surface sites versus molecular analogs. *J. Am. Chem. Soc.* **2016**, *138*, 14987-14997.

(10) Liu, L.; Li, H.; Zhou, H.; Chu, S.; Liu, L.; Feng, Z.; Qin, X.; Qi, J.; Hou, J.; Wu, Q.; Li, H.; Liu, X.; Chen, L.; Xiao, J.; Wang, L.; Xiao, F. Rivet of cobalt in siliceous zeolite for catalytic ethane dehydrogenation. *Chem* **2023**, *9*, 637-649.

(11) Yu, K.; Srinivas, S.; Wang, C.; Chen, W.; Ma, L.; Ehrlich, S.; Marinkovic, N.; Kumar, P.; Stach, E.; Caratzoulas, S. High-temperature pretreatment effect on Co/SiO_2 active sites and ethane dehydrogenation. *ACS Catal.* **2022**, *12*, 11749-11760.

(12) Pavlov, V.; Bruter, D.; Konnov, S.; Ivanova, I. Effect of silica source on zeolite MFI crystallization in fluoride media and its physicochemical and catalytic properties. *Micropore. Mesopore. Mat.* **2022**, *341*, 112088.

(13) Dijkstra, T.; Duchateau, R.; van Santen, R.; Meetsma, A.; Yap, G. Silsesquioxane models for geminal silica surface silanol sites: A Spectroscopic investigation of different types of silanols. *J. Am. Chem. Soc.* **2002**, *124*, 9856-9864.

(14) Rodriguez-Gomez, A.; Holgado, J.; Caballero, A. Cobalt Carbide Identified as Catalytic Site for the Dehydrogenation of Ethanol to Acetaldehyde. *ACS Catal.* **2017**, *7*, 5243-5247.

(15) Allotta, P.; Stair, P. Time-resolved studies of ethylene and propylene reactions in zeolite H-MFI by *in-situ* fast IR heating and UV Raman spectroscopy. *ACS Catal.* **2012**, *2*, 2424-2432.

(16) Khan, S.; Godahewa, S.; Wimalasiri, P.; Thompson, W.; Scott, S.; Peters, B. Modeling the structural heterogeneity of vicinal silanols and its effects on $TiCl_4$ grafting onto amorphous silica. *Chem. Mater.* **2022**, *34*, 3920-3930.

(17) Xu, Y.; Yu, W.; Zhang, H.; Xin, J.; He, X.; Liu, B.; Jiang, F.; Liu, X. Suppressing C–C bond dissociation for efficient ethane dehydrogenation over the isolated Co(II) sites in SAPO-34. *ACS Catal.* **2021**, *11*, 13001-13019.

(18) Hu, W.; Xu, Y.; Xin, J.; Liu, B.; Jiang, F.; Liu, X. Stable co-production of olefins and aromatics from ethane over Co^{2+}-exchanged HZSM-5 zeolite. *Catal. Sci. Technol.* **2022**, *12*, 3716-3726.

(19) Xu, Y.; Hu, W.; Li, Y.; Su, H.; Liang, W.; Liu, B.; Gong, J.; Liu, Z.; Liu, X. Manipulating the cobalt species states to break the conversion-selectivity trade-off relationship for stable ethane dehydrogenation over ligand-free-synthesized Co@MFI catalysts. *ACS Catal.* **2023**, *13*, 1830-1847.

(20) Wang, X.; Li Y.; Wu, W.; Xu, Y.; Liu, X. Mesoporous Co@MCM-41 catalyst for stable ethane dehydrogenation. *Carbon Resources Conversion* **2025**, *8*, 100244.

(21) Liu, J.; He, N.; Zhang, Z.; Yang, J.; Jiang, X.; Zhang, Z.; Su, J.; Shu, M.; Si, R.; Xion, G.; Xie, H.; Vilé, G. Highly-dispersed zinc species on zeolites for the continuous and selective dehydrogenation of ethane with CO_2 as a soft oxidant. *ACS Catal.* **2021**, *11*, 2819-2830.

(22) Zheng, Y.; Li, J.; Zhang, X.; Li, S.; An, J.; Chen, F.; Li, X.; Zhu, X. Evolution of Co species in CO_2-assisted ethane dehydrogenation: competing cleavage of C–H and C–C bonds. *ACS Catal.* **2024**, *14*, 4749-4759.

(23) Yang, Z.; Li, H.; Zhou, H.; Wang, L.; Wang, L.; Zhu, Q.; Xiao, J.; Meng, X.; Chen, J.; Xiao, F. Coking-resistant iron catalyst in ethane dehydrogenation achieved through siliceous zeolite modulation. *J. Am. Chem. Soc.* **2020**, *142*, 16429-16436.

(24) De, S.; Ould-Chikh, S.; Aguilar, A.; Hazemann, J.; Zitolo, A.; Ramirez, A.; Telalovic, S.; Gascon, J. Stable Cr-MFI catalysts for the nonoxidative dehydrogenation of ethane: Catalytic performance and nature of the active sites. *ACS Catal.* **2021**, *11*, 3988-3995.

(25) Maeno, Z.; Yasumura, S.; Wu, X.; Huang, M.; Liu, C.; Toyao, T.; Shimizu, K. Isolated indium hydrides in CHA zeolites: Speciation and catalysis for nonoxidative dehydrogenation of ethane. *J. Am. Chem. Soc.* **2020**, *142*, 4820-4832.

(26) Muhlenkamp, J.; LiBretto, N.; Miller, J.; Hicks, J. Ethane dehydrogenation performance and high temperature stability of silica supported cobalt phosphide nanoparticles. *Catal. Sci. Technol.* **2022**, *12*, 976-985.

(27) He, H.; Miao, C.; Guo, H.; Hua, W.; Yue, Y.; Gao, Z. Ethane dehydrogenation over Co-based MOR zeolites. *Reac. Kinet. Mech. Cat.* **2022**, *135*, 2045-2058.

(28) Guo, H.; Miao, C.; Guo, H.; Hua, W.; Yue, Y.; Gao, Z. Cobaltous oxide supported on MFI zeolite as an efficient ethane dehydrogenation catalyst. *Micropore. Mesopore. Mat.* **2021**, *312*, 110791.

(29) Wu, L.; Fu, Z.; Wei, J.; Deng, H.; Zhang, Y.; Tang, Y.; Tan, L. The investigation into the dehydroaromatization of ethane over cobalt-modified ZSM-5 catalyst. *Micropore. Mesopore. Mat.* **2022**, *343*, 112159.

(30) Qiu, B.; Zhang, Y.; Zhang, Y. A Stable zinc zeolite catalyst for dehydrogenation of ethane to aromatics and ethylene. *Catal. Lett.* **2022**, *152*, 1372-1385.

(31) Chen, S.; Chang, X.; Sun, G.; Zhang, T.; Xu, Y.; Wang, Y.; Pei, C.; Gong, J. Propane dehydrogenation: Catalyst development, new Chemistry, and emerging technologies. *Chem. Soc. Rev.* **2021**, *50*, 3315-3354.
